# Transcriptional identification of genes light-interacting in the extraretinal photoreceptors of the crayfish *Procambarusclarkii*

**DOI:** 10.3897/zookeys.1072.73075

**Published:** 2021-11-19

**Authors:** Gabina Calderón-Rosete, Juan Antonio González-Barrios, Celia Piña-Leyva, Hayde Nallely Moreno-Sandoval, Manuel Lara-Lozano, Leonardo Rodríguez-Sosa

**Affiliations:** 1 Departamento de Fisiología, Facultad de Medicina, Universidad Nacional Autónoma de México, Ciudad Universitaria, C. P. 04510, México Universidad Nacional Autónoma de México Ciudad de México Mexico; 2 Laboratorio de Medicina Genómica, Hospital Regional “Primero de Octubre” ISSSTE, 07300, México Laboratorio de Medicina Genómica Ciudad de México Mexico; 3 Departamento de Fisiología, Biofísica y Neurociencias, Centro de Investigación y Estudios Avanzados, 07360, México Centro de Investigación y Estudios Avanzados Ciudad de México Mexico

**Keywords:** Caudal photoreceptor, opsins, photoresponse, phototransduction, pleonal nerve cord

## Abstract

Crayfish serve as a model for studying the effect of environmental lighting on locomotor activity and neuroendocrine functions. The effects of light on this organism are mediated differentially by retinal and extraretinal photoreceptors located in the cerebroid ganglion and the pleonal nerve cord. However, some molecular aspects of the phototransduction cascade in the pleonal extraretinal photoreceptors remain unknown. In this study, transcriptome data from the pleonal nerve cord of the crayfish *Procambarusclarkii* (Girard,1852) were analyzed to identify transcripts that potentially interact with phototransduction process. The Illumina MiSeq System and the pipeline Phylogenetically Informed Annotation (PIA) were employed, which places uncharacterized genes into pre-calculated phylogenies of gene families. Here, for the first time 62 transcripts identified from the pleonal nerve cord that are related to light-interacting pathways are reported; they can be classified into the following 11 sets: 1) retinoid pathway in vertebrates and invertebrates, 2) photoreceptor specification, 3) rhabdomeric phototransduction, 4) opsins 5) ciliary phototransduction, 6) melanin synthesis, 7) pterin synthesis, 8) ommochrome synthesis, 9) heme synthesis, 10) diurnal clock, and 11) crystallins. Moreover, this analysis comparing the sequences located on the pleonal nerve cord to eyestalk sequences reported in other studies reveals 94–100% similarity between the 55 common proteins identified. These results show that both retinal and pleonal non-visual photoreceptors in the crayfish equally expressed the transcripts involved in light detection. Moreover, they suggest that the genes related to ocular and extraocular light perception in the crayfish *P.clarkii* use biosynthesis pathways and phototransduction cascades commons.

## Introduction

The freshwater crayfish is a model for studying locomotor behavioral and neurohormonal responses to light, which are mediated by retinal and extraretinal photoreceptors. The crayfish’s pleonal nerve cord (PNC), which consists of six ganglia, also responds to photostimulation previous studies have reported motor neuron activation (Edwards 1984; Simon and Edwards 1990). As early studies postulated, light-induced reflex activity results from integrating luminous sensory information from an interplay between the transmissions of retinal and caudal photoreceptors (CPRs) (Rodríguez-Sosa et al. 2008).

In the invertebrate phototransduction mechanism, light initiates a signaling cascade that induces a depolarization of the cell membrane. One CPR is present in each half of the sixth pleonal ganglion (6^th^ PG), with their axons coursing rostrally from the 6^th^ PG to the brain. CPRs respond to a light stimulus with a high-frequency burst. In addition, these neurons respond trans-synaptically to mechanical stimuli. The CPR has been well-studied through electrophysiological recordings, along with analyses of the locomotor activity induced when sensing light (Welsh 1934; Wilkens and Larimer 1972; Edwards 1984; Fernández de Miguel and Aréchiga 1992). Serotonin and dopamine regulate the firing rate from these CPRs, and serotonin modulates the circadian rhythm for both spontaneous and light-induced CPR activities (Rodríguez-Sosa et al. 2006, 2007, 2011).

The CPRs are “simple” photoreceptors due to their lack of specialized structures such as the microvilli or cilia that characterize the retinal photoreceptor (Gotow and Nishi 2008). These structural differences between ocular and extraocular receptors suggest differences in the molecular cascades involved in photoreception. However, a recent study shows that two opsins are found in both the retina and the PNC of the crayfish *P.clarkii* (Kingston and Cronin 2015). This study also finds that the transcripts of both opsins are expressed in each ganglion of the PNC and in the retina with identical sequences, suggesting that CPRs use these two proteins in the phototransduction pathway, as observed in the retina by Hariyama et al. (1994).

The opsins identified include one that is sensitive to short-wavelength light (λmax = 440 nm, SWS, blue) and another sensitive to long-wavelength light (λmax = 530 nm, LWS, green). Other studies show that these simple photoreceptors have a spectral sensitivity peak at 500 nm, suggesting that they contain a rhodopsin-like photopigment (Bruno and Kennedy 1962; Larimer et al. 1966; Cronin and Goldsmith 1982;). In addition, the left and right crayfish caudal photoreceptors show asymmetry in the spontaneous action potentials discharged in darkness and in their responses to white light and blue or green monochromatic light pulses (Sánchez-Hernández et al. 2018; Pacheco-Ortiz et al. 2018).

Furthermore, a study seeking to identify the molecular mechanism of CPR transduction finds that the injection of inositol 1,4,5-trisphosphate (IP3), calcium, and guanosine nucleotide (GTP) mimics the light response (Kruszewska and Larimer 1993). However, for crustaceans, little genomic information is available, and few sequences have been annotated in databases regarding the components involved in phototransduction cascades in extraretinal photoreceptors (Hariyama et al. 1993; Kingston and Cronin 2015; Porter et al. 2017).

In this study, we obtain and analyze the pleonal nerve cord transcriptome to identify potential light-interacting genes from the extraretinal photoreceptors of the freshwater crayfish *P.clarkii*. We also compare the encoded protein to the sequences of the eyestalk transcriptome reported in a study by Manfrin et al. (2015). All sequencing data reported here have been deposited in the GenBank database.

## Materials and methods

We used four adult crayfish (*P.clarkii*) two males and two females in their intermolt stage. The animals were acquired from a local provider in the autumn and kept in the laboratory in aerated water containers for two weeks before the experiments, with a program of 12:12 h light-dark cycles; they were fed with carrots and dried fish. The care and handling of the animals during the experimental procedures was carried out according to the policies established by the Ethics Commission. This study was approved by Research of the Faculty of Medicine, UNAM (code FM/DI/128/2019).

The pleonal nerve cords were dissected and immediately placed in the Eppendorf tube with precooled TRIzol. The tissue was preserved at -80°C prior to extraction, the tissue was homogenized manually with a precooled mortar and pestle. Total RNA was extracted from the pleonal nerve cord using TRIzol reagent following the manufacturer’s protocol (Catalog number 15596018, Invitrogen Co., Carlsbad, CA, USA). TRIzol solubilizes the biological material after the addition of chloroform (Catalog number P3803, Sigma-Aldrich, St. Louis, MO, USA), producing three phases: the upper aqueous phase containing RNA, the interphase with DNA, and the organic phase containing proteins. The aqueous phase was transferred to a new tube; the RNA was precipitated with isopropanol (Catalog number I9516, Sigma-Aldrich, St. Louis, MO, USA) and collected via centrifugation; the pellet was then washed with 75% ethanol (Catalog number E7023, Sigma Aldrich Co., St. Louis, MO, USA). The ethanol was then removed, and the pellet was resuspended in RNase-free H_2_O and stored at -80°C. We used 5μg of total RNA to obtain the cDNA libraries, according to the manufacturer’s protocol for the Illumina TruSeq RNA Library Preparation Kit v2 (Catalog number RS-122-2001, Illumina, San Diego, CA, USA). We performed Illumina paired-end protocol 150 bp sequencing. The library obtained was sequenced using the MiSeq Reagent kit v3 system (Catalog number MS-102-3001) according to the manufacturer’s protocol, to obtain the PNC transcriptome.

The raw data from the Illumina system were uploaded to the Galaxy Web Portal to execute a *de novo* assembly process, using Trinity software (Grabherr et al. 2011; Haas et al. 2013; Afgan et al. 2016). The reads had quality scores higher than 30, so we did not conduct any procedure to eliminate low-quality sequences. The adapter sequences were trimmed, and we performed the de novo transcriptome assembly using Trinity software, obtaining sequences in FASTA files in the Galaxy platform. Their translation was executed automatically via the OSIRIS pipeline (Oakley et al. 2014).

The resulting sequences were processed via the “Get ORFs” program. Any sequences shorter than 100 amino acids were ignored, to produce the protein sequences to be analyzed (Rice et al. 2000; Blankenberg et al. 2007). Next, we used the “Phylogenetically informed annotation” (PIA) pipeline to analyze the transcriptomic sequences from the PNC to search genes involved in light detection (Speiser et al. 2014), this pipeline is available on the Galaxy bioinformatics platform https://galaxyproject.org/use/pia/.

The PIA pipeline uses tools to generate maximum-likelihood phylogenetic trees for 109 genes from a Light Interaction Toolkit (LIT), a gene collection regarding light-interacting structures and their functions and development in metazoans, including those in phototransduction, eye development, pigment synthesis, circadian cycles, and other light-interacting pathways; these genes are distributed across 13 functional gene sets. This bioinformatics program places uncharacterized genes into a gene family based in pre-calculated phylogeny in a secure and accessible web server. We used the e-value 1e^-20^ for a BLAST search of the cutoff.

The analysis with PIA generates two results files based on the functional set of genes that are selected for analyzing the amino acid sequences. One file contains the number and sequence with all the hit proteins retrieved by the initial BLAST search, while the other file contains all selected genes placed onto their corresponding gene trees. All PIA pipeline filtered transcripts were manually analyzed to determine which sequences correspond to the possible genes implicated within the photoreception process. This procedure facilitates the elimination of duplicates and fragments and the identification of overlapping sequence sections to integrate longer sequences. For protein sequence identification, we used the Prosite database to verify the preserved domain profiles; we correlated them with functions, using the Pfam or UniProt databases (https://pfam.xfam.org/search; https://www.uniprot.org). The amino acid sequences listed in the Suppl. material 1 identified as ‘mmc3’ in the Manfrin’s study (2015), were used to assess the similarity of sequences identified in the PNC and in the eyestalk, using alignments with the Clustal Omega program (https://www.ebi.ac.uk/Tools/msa/clustalo/).

This procedure facilitates the verification of sequence identities obtained via the PIA analysis; these sequence data have been submitted to the GenBank databases under the accession number indicated in the fourth column of Tables 1–7.

## Results

The Illumina system displayed 40,867,860 raw data reads; with the Novo assembler Trinity software available on the Galaxy website, we obtained 53,967 assembled nucleotides sequences in FASTA files. The PIA phylogenetic analysis was carried out using 36,558 deduced amino acid sequences with open reading frames and a minimum length of 100 amino acids. The sequence translations were done automatically in the OSIRIS platform available on the Galaxy site. The PIA analysis generated 109 maximum-likelihood trees distributed across thirteen functional gene sets, using the metazoan Light Interaction Toolkit; with the software, we obtained results for all sets from the PNC transcriptome.

We combined the genes identified in the functional gene sets “Retinoid pathway vertebrate” and “Retinoid pathway invertebrate” into Set 1. Set 2 includes the functional gene set “Photoreceptor specification and retinal determination network”; thus, we present a total of 11 gene sets in 7 Tables. This filter identified 256 sequences with potential homology with some functional gene sets from the PIA pipeline. After the analysis for each sequence, we eliminated duplicate sequences; we obtained longer consensus sequences when the ends of shorter sequences overlapped correctly. Finally, we integrated a total of 62 different transcripts from the pre-calculated phylogenetic trees. The BLAST analyses for each of the amino acid sequences identified in *P.clarkii* show a high conservation grade (≥ 90 %) with some other crustacean species, especially the Pacific white shrimp *Penaeusvannamei*.

In addition, we compared the sequences that we identified in the transcriptome of the PNC to the sequences from the transcriptome of the eyestalk. As mentioned previously, the sequences used for this comparison were obtained directly from Table mmc3, included as Suppl. material 1 by Manfrin et al. (2015). In our study, all comparisons with the eyestalk refer to this study. To ensure positive results, we performed a search in Table mmc3 with the Excel search tool, using both the name of the identified protein and the sequence itself.

The Tables show the names of the sequences we identified in the PNC, the number of amino acids (as deduced from the nucleotides), and the accession number in GenBank, as well as a comparison with previously reported sequences in the eyestalk. The last column shows the identity percentage between both sequences. We identified 62 genes from the PNC 55 of these were also expressed in the eyestalk transcriptome, while 38 were 100% identical to their corresponding transcripts in the PNC; 19 sequences had 94–99% similarity, while two transcripts presented a similarity of 24–41% with the transcript of the same name from the eyestalk. Only five PNC identified genes were not found in the eyestalk transcriptome.

The first functional gene set in Table 1 contains eight elements that participate in the synthesis and metabolism of visual chromophores from dietary carotenoid precursors. This group includes the genes identified in two functional sets by PIA (namely, the retinoid pathways of vertebrates and invertebrates). Almost all identified sequences perform an enzymatic function, except for the sequence with a match for the type-B scavenger receptor, which has been reported to mediate the cellular capture of carotenoids in *Drosophila* (Kiefer et al. 2002; Von Lintig et al. 2005). In this set, out of the eight sequences identified in the abdominal nerve cord, only 6 were also identified in the eyestalk. The two sequences not identified in the eyestalk were retinol dehydrogenase 13 and cellular retinoic acid-binding protein 1.

**Table 1.** Transcripts identified from PNC through PIA pipeline compared with crayfish eyestalk transcriptome data.

	**Pleonal nerve cord (Current study)**			**Eyestalk (Manfrin et al. 2015)**		
**Gene**	**Top BLAST hit-Protein**	**aa**	**Access number**	**Contig ID (Procl_ES)**	**aa**	**Homology percentage**
**Set 1. Components of the retinoid pathway in vertebrates and invertebrates**						
*Ralbp*	Retinal-binding protein	159	MN110026	5420_1	431	100
*Rdh11*	Retinol dehydrogenase 11	346	MT601680	12053_0	350	100
*Rdh13*	Retinol dehydrogenase 13	149	MT601681	WCS	–	–
*Dhrs4*	Dehydrogenase/reductase SDR family member 4-like	289	MT601679	888_7	282	100
*Sdr16c5*	Epidermal retinol dehydrogenase 2-like isoform X2	122	MT601682	5911_0	309	98
*Crabp1*	Cellular retinoic acid-binding protein 1-like	115	MT601683	WCS	–	–
*ninaB*	Carotenoid oxygenase (RPE65)	108	MT601684	4243_0	523	41
*ninaD*	Class B scavenger receptor	111	MT942649	2476_0	515	100

PNC= Pleonal nerve cord; aa= amino acids; WCS= whithout comparable sequence in the eyestalk as in all tables.

The eyestalk transcriptome contains two sequences denominated as retinol dehydrogenase 13 (Procl_ES_4929_1 and Procl_ES_29212_0), although they showed similarities of 46% and 44%, respectively, with the sequence that we identified in the PNC.

The sequence identified as Cellular retinoic acid binding protein 1 (CRABP) contains the domain that corresponds to the Lipocalin/cytosolic fatty-acid-binding protein family. Lipocalins are transporters for small hydrophobic molecules, such as lipids, steroid hormones, bilins, and retinoids. Cytosolic CRABPs may regulate the access of retinoic acid to the nuclear retinoic acid receptors (www.uniprot.org/uniprot/P40220).

Notably, in this set, we found a low identity grade (41%) between PNC and eyestalk sequences for the protein encoded by the *ninaB* gene, the carotenoid oxygenase. Carotenoid oxygenases are a family of enzymes involved in carotenoid cleavage to produce retinol, commonly known as vitamin A. There are five sequences reported in the eyestalk transcriptome (Procl_ES_659_0; 4243_0: 11203_0; 30934_0: 1244_0). All of them, including the PNC sequences, contain the RPE65 superfamily conserved domain. However, they have very low similarity among themselves (see https://doi.org/10.5061/dryad.pg4f4qrqp).

Set 2 in Table 2 includes the PIA identified genes for two functional sets: Photoreceptor specification and Retinal determination network. Ten genes are identified: all are putatively implicated in developmental processes such as axon morphogenesis (*Glass*), eye formation via regulation of the initial specification of retinal cells (*Pph*; *En*), and development or differentiation (*Notch*). The Hedgehog protein is believed to play an important role in one of the fundamental signal transduction pathways; its homeodomain contains sequence-specific DNA-binding proteins that act as regulators of transcription (Wang et al. 2020). During embryogenesis, morphogenic pathways such as WNT and Hedgehog are constitutively active; however, the activity of these pathways decreases in adulthood. Interestingly we identified both morphogenes and the genes of proteins involved in their pathways (Frizzled was identified in a manual analysis (GenBank: MZ383818 and En) in the PNC. Notably, the Hedgehog and engrailed-1 sequences identified in the PNC were not identified in the eyestalk.

**Table 2.** Transcripts identified from the PNC through PIA pipeline compared with the crayfish eyestalk data.

	Pleonal nerve cord (Current study)			Eyestalk (Manfrin et al. 2015)		
Gene	Top BLAST hit-Protein	aa	Access number	Contig ID (Procl_ES)	aa	Homology percentage
Set 2. Elements of photoreceptor specification and retinal determination network.						
*Egfr*	Tyrosine-protein kinase Fer	873	KY974273	3891_0	914	100
*Pph*	Putative retinal homeobox protein Rx2-like	414	MN110016	1058	422	100
*Glass*	Krueppel homolog 1-like	608	MN110021	652_0	608	100
*En*	Homeobox protein engrailed-1-like isoform X1	184	MN110023	WCS	–	–
*notch*	Neurogenic locus Notch protein	1210	MN110012	9959	2464	100
*Hh*	Protein hedgehog-like	190	MN110017	WCS	–	–
*dlx2b*	Homeobox protein DLX2b-like	299	MT942642	33351_0	162	94
*Dlx6*	Homeobox protein DLX-6-like	305	MT942643	18254_0	337	100
*Zag-1*	Zinc finger E-box-binding homeobox protein zag-1-like	204	MT942647	12586_0	831	99
*Zfhx3*	Zinc finger homeobox protein 3-like	768	MT942648	6525_0	2596	99

Set 3 of the genes, corresponding to the rhabdomeric phototransduction pathway associated with invertebrate eyes, had the highest number of PIA-identified genes, totaling 16 transcripts (Set 3, Table 3). Opsins are light receptors that activate G-protein pathways through cAMP, IP3, and DAG. This pathway is important for inducing depolarization in invertebrate photoreceptors. We identified the codified region of the alpha subunit of several types of G-proteins (including Gq), as well as phospholipase C (PLC), which is important for processing diacylglycerol (DAG) from PIP2. We also identified guanine nucleotide-binding protein subunit beta 5, which is involved in the termination of signaling initiated by the G protein-coupled receptors, as well as beta arrestin-1, an important regulatory element in the phototransduction pathway. This protein participates in receptor desensitization and resensitization processes. In this set, the PIA analysis also identified the gene nonA, which encodes a putative RNA-binding protein in *Drosophila*; its absence has been associated with an electroretinogram defect and reduced visual acuity in fly mutants (Jones and Rubins 1990; Rendahl et al. 1996). In this set, 15 sequences were common to both structures, with a high identity of 96–100%. The *P.clarkii* eyestalk transcriptome has 19 sequences identified as Arrestin; however, none was similar to the beta-arrestin-1 that we identified in the PNC.

**Table 3.** Transcripts identified from PNC throught PIA pipeline compared with crayfish eyestalk transcriptome data.

	**Pleonal nerve cord (current study)**			**Eyestalk (Manfrin et al. 2015)**		
**Gene**	**Top BLAST hit-Protein**	**aa**	**Access number**	**Contig ID (Procl_ES)**	**aa**	**Homology (Percentage)**
**Set 3. Elements of the rhabdomeric phototransduction pathway**						
*Rdgc*	Serine/threonine protein phosphatase 1	329	MN110024	983	329	100
*Ppp2cb*	Serine/threonine-protein phosphatase 2A catalytic subunit beta isoform	309	MN110029	1697	309	100
*G alpha*	Guanine nucleotide-binding protein G(q) subunit alpha	353	MF279133	1935_0	353	94
	Guanine nucleotide-binding protein G(s) subunit alpha	379	MN110031	1880_0	285	100
	Guanine nucleotide-binding protein G(i) subunit alpha	355	MN110025	6610_0	355	100
	Guanine nucleotide-binding protein G(o) subunit alpha	262	MN110018	2664_0	354	100
*G beta*	Guanine nucleotide-binding protein subunit beta-5-like	189	MN110034	5560_0	354	98
*Gnb1*	Guanine nucleotide-binding protein G(I)/G(S)/G(T) subunit beta-1	340	KY974308.1	1098_0	340	100
*Ggamma1*	Guanine nucleotide-binding protein subunit gamma-1	100	MT601685	3444_0	102	100
*nonA*	Protein no-on-transient A	467	MN110015	–	–	–
*Dagk*	Eye-specific diacylglycerol kinase isoform X3	902	MF279134	1599_0	467	100
*Plc*	1-Phosphatidylinositol 4,5-bisphosphate	733	MN110020	3323_0	1005	95
	Phosphodiesterase delta-4-like			2268_0	904	96
*Pkc*	cAMP-dependent protein kinase catalytic subunit 1	352	MN110019	2373	507	96
	Protein kinase C	602	MN110035	5727_0	747	100
*Arr*	Beta-arrestin 1	263	MN110013	WCS	–	–
*rdgB*	Phosphatidylinositol transfer protein beta isoform-like	270	MN110014	2227_0	270	100
**Set 4. Opsins**						
*moody*	Putative G-protein coupled receptor moody-like	504	MT601688	13547_0	739	100
*moody*	G-protein coupled receptor moody-like isoform X2	407	MT601689	6096_0	411	99
	Short wavelength-sensitive opsin	391	ALJ26468	11143_0	391	99
	Long wavelength-sensitive opsin	377	ALJ26467	23_0	377	100

In Set 4, the PIA pipeline identified two transcripts (Table 3); these sequences were two isoforms of the G protein-coupled receptor moody-like. We decided to keep these sequences in Table 3 because the conserved domains in these proteins are characteristic of the G protein-coupled receptors. This family contains several opsin family members that are typical rhodopsin superfamily members. This set also contains two opsin sequences previously reported by other authors; although we did not identify them in the current analysis carried out with the PIA analysis, we consider it convenient to include them here because their expression in the PNC has already been reported (Kingston and Cronin 2015).

Set 5 includes genes identified by PIA analysis in the phylogenetic family of signaling cascades in ciliary photoreceptors (Table 4). The ciliary photoreceptors are traditionally associated with vertebrate eyes; however, several transcripts included in the phylogenetic tree from PIA for this type of photoreceptor were identified in the PNC transcriptome. Potential’s regulators of G-protein signaling predominate in this group; the neurocalcin homolog has 96% identity with *Drosophilamelanogaster*’s reported sequence and with neuronal calcium sensor 2-like protein (alignments not shown), which is another regulator of G protein-coupled receptors that act in a calcium-dependent manner. In this set, all sequences were also identified in the eyestalk, with 99–100% identity.

**Table 4.** Transcripts identified from PNC through PIA pipeline compared with crayfish eyestalk transcriptome data

	**Pleonal nerve cord (current study)**			**Eyestalk (Manfrin et al. 2015)**		
**Gene**	**Top BLAST hit-Protein**	**aa**	**Access number**	**Contig ID (Procl_ES)**	**aa**	**Homology percentage**
**Set 5. Components of ciliary phototransduction**						
*Rcvrn*	Neurocalcin homolog isoform X2	192	MN110027	2966_0	192	99
*ncs-2*	Neuronal calcium sensor 2-like	188	MN110022	3948_0	188	100
*Rgs9*	Regulator of G-protein signaling 9-like	170	MN110033	5623_0	962	100
	Regulator of G-protein signaling 7-like	125	MN110036	3602_1	486	99
	Putative regulator of G protein signaling	255	MN110028	4872_0	1534	100

Sets 6-9 contain enzymes in several pigment biosynthesis pathways (Table 5). Prophenoloxidase activates the cascade to synthesize melanin, while cysteine sulfonic acid decarboxylase is part of the taurine biosynthesis pathway, which is related to various biological processes in response to cAMP. Sets 7 and 8 encompass enzymes that participate in the synthesis pathways of several pigments, such as brown ommochromes and red drosopterins. Both contribute to the typical eye color phenotype of *Drosophila* and serve as light-screening pigments; these are several types of pigments that have been reported in the integument underlying the exoskeleton and in the compound eyes of some arthropods (Ziegler 1961; Cerenius et al. 2008; Kim et al. 2013).

**Table 5.** Transcripts identified from PNC through PIA pipeline compared with crayfish eyestalk transcriptome data.

	Pleonal Nerve Cord (Current study)			Eyestalk (Manfrin et al. 2015)		
Gene	Top BLAST hit-Protein	aa	Access Number	Contig ID (Prcl_ES)	aa	Homology (Percentage)
Set 6. Elements of melanin synthesis pathway						
*Csad*	Cysteine sulfinic Acid Decarboxylase	417	MN110038	4782_0	603	100
*Ppo*	Prophenoloxidase	441	MH156427	2348_0	495	99
**Set 7. Elements of pterin synthesis pathway**						
*Xdh*	Aldehyde oxidase	435	MN110003	7559_0	1314	99
	Indole-3-acetaldehyde oxidase-like	536	MN110004	8366_0	1340	99
*Sepia*	Pyrimidodiazepine synthase	241	MN110006	5690_1	102	100
*Dhpr*	Dihydropteridine reductase-like	235	MN110005	1504_0	235	100
*Pcd*	Pterin-4-alpha-carbinolamine dehydratase-like	101	MN110007	2287_0	157	100
*Spr*	Sepiapterin reductase-like	185	MN110009	12527_0	274	100
**Set 8. Elements of ommochrome synthesis pathway**						
*Abcg1*	ATP-binding cassette sub-family G member 1-like	156	MN110008	6760_0	700	100
	ABC transporter, subfamily ABCB/MDR	270	MT942646	8046_0	1341	100
*Alad*	Delta-aminolevulinic acid dehydratase	280	MN110039	3984_0	338	100
*Alas2*	5-aminolevulinate synthase, Erythroid-specific, Mitochondrial-like isoform X5	215	MT942644	2230_0	534	99
*Uros*	Uroporphyrinogen-III synthase	252	MH156441	5238_0	345	99
*Urod*	Uroporphyrinogen decarboxylase	107	MN110037	4848_0	359	100

In the Ommochrome synthesis set, we recognized the scarlet-brown gene that encodes an ATP-binding domain of the ABC transporters family. This is a water-soluble domain of transmembrane ABC transporters; it uses the hydrolysis of ATP to translocate a variety of compounds across biological membranes and is also responsible for the transportation of guanine, tryptophan, and histamine precursors of eye pigments in planthopper (Jiang and Lin 2018), and *Drosophilamelanogaster* (Borycz et al. 2008).

Set 9 in Table 5 contains 4 enzymes related to the Heme B biosynthesis pathway, one of the best-known complexes of the porphyrin family. The porphyrinoid pigments play crucial roles in protection against UV light (Martins et al. 2019), and in the processes of circadian rhythm maintenance and metabolism (Carter et al. 2017).

Because light is the primary synchronizer in the regulation of circadian rhythms, the PIA pipeline facilitates identification of some transcripts related to the molecular pathway of the circadian clock. In Set 10 of Table 6, we identify a partial transcript of the Calcium-activated potassium channel transcript in crayfish. In *Drosophila*, this channel was sequenced by Atkinson et al. (1991). This potassium channel is activated by membrane depolarization and by increases in cytosolic Ca^2+^; it mediates the export of K^+^. We identified the partial sequence of a transcript that allowed us to deduce a 263-amino acid fragment. This fragment has 100% identity grade to the sequence from the eyestalk of crayfish (Procl ES_4724_0) and has similarity of 92% to the 1184-amino acid sequence from *Drosophilamelanogaster* (GenBank: AAA28902.1) (see https://doi.org/10.5061/dryad.pg4f4qrqp) .

**Table 6.** Transcripts identified from PNC though PIA pipeline compared with crayfish eyestalk transcriptome data

	Pleonal Nerve cord (current study)			Eyestalk (Manfrin et el. 2015)		
Gene	Top BLAST hit-Protein	aa	Access Number	Contig ID (Procl_ES)	aa	Homolog Percentage
Set 10. Elements identified in the set of circadian clock						
*Slo*	Calcium-activated potassium channel variant 4	263	QIA97593	4724_0	1172	100
*Lark*	RNA-binding protein lark	308	QIA97594	2543_0	308	100

The identified gene *lark* in PNC has 100% identity to the corresponding sequence identified in the eyestalk (Procl_ES_2543_0) of the crayfish; it is 52% similar to that found in *Drosophilamelanogaster* (GenBank: Q94901.1) (see https://doi.org/10.5061/dryad.pg4f4qrqp).

Set 11 contains transcripts related to soluble proteins called crystallins (Table 7). Crystallins are water-soluble proteins; in vertebrates, the refractive index of the lenses depends on the concentrations of these proteins. Previous research has proposed that, in vertebrates, crystallins have been recruited from stress-protective proteins as small heat-shock proteins (Tomarev and Piatygorsky 1996). In the PNC transcriptome of crayfish, we have identified the transcript of the alpha-crystallin A chain, as well as 2 enzymes related to crystallins identified in cephalopods (S-crystallins and Ω-crystallins). In this phylogenetic family, the PIA pipeline also allows us to identify the transcript that encodes the small heat-shock protein that contains the alpha-crystallin domain (ACD) of alpha-crystallin-type small heat-shock proteins (sHsps). sHsps are small stress-induced proteins. In this set, we also identify hypoxia-inducible factor 1 alpha, which contains the PAS domain. PAS domains have been found to bind ligands and act as sensors for light and oxygen in signal transduction (https://www.ncbi.nlm.nih.gov/Structure/cdd/cddsrv.cgi). These sequences were also identified in the eyestalk transcriptome, with 96–100% similarity; the alpha-crystallin A chain shows 24% similarity with the sequences in the eyestalk.

**Table 7:** . Transcripts identified from PNC through PIA pipeline compared with crayfish eyestalk transcriptome data

	**Pleonal Nerve Cord (Current study)**			**Eyestalk (Manfrin et al. 2015)**		
**Gene**	**Top BLAST hit-Protein**	**aa**	**Access Number**	**Contig ID (Procl_ES)**	**aa**	**Homology Percentage**
**Set 11. Elements associated with crystalline proteins**						
*GstS1*	Glutathione S-transferase theta	221	MH156430.1	5690_1	241	100
*Aldh*	Aldehyde dehydrogenase (omega-crystallin)	523	MN110030	2528_0	523	100
*Cryaa*	Alpha-crystallin A chain	139	MT601686	721_0	163	24
*ibpB*	Small heat shock protein,	184	MG910470	554_0	184	100
*hif1an*	Hypoxia inducible factor 1 alpha	1054	MW981273	2830_0	523	96

All genes identified here from the PNC were edited for annotation and submitted to the GenBank database of the National Center for Biotechnology Information (NCBI); the assigned accession number appears in the fourth column of each table. We have included Suppl. material 1 with all sequences available in the GenBank database (www.ncbi.nlm.nih.gov/genbank; see https://doi.org/10.5061/dryad.pg4f4qrqp).

## Discussion

Invertebrates preserve various organs to sense light. In addition to retinal photoreceptors, crayfish possess extraretinal photoreceptors in the cerebroid ganglion and the abdominal nerve cord. These photoreceptor groups contribute differentially to phototactic motor behaviors and the synchronization of circadian rhythms (Wilkens and Larimer 1972; Edwards 1984; Simon and Edwards 1990; Fernández-de-Miguel and Aréchiga 1992; Rodríguez‐Sosa et al. 2008; Sullivan et al. 2009; Rodríguez-Sosa et al. 2012).

We present in this study the putative molecular components of the extraocular phototransduction system identified from the transcriptome of the PNC of the crayfish *P.clarkii*. We identify 62 transcripts that encode proteins potentially involved in the development processes of photoreceptor structures, phototransduction cascades, pigment biosynthesis, crystalline structures, and circadian rhythms. This constitutes the first report on the comprehensive identification of genes with a putative functional identification in extraretinal phototransduction from the PNC of the crayfish *P.clarkii*.

The genetic information on the PNC in this study allows us to make comparisons to the eyestalk transcriptome of the same species, as reported by Manfrin et al. (2015). The comparison between the proteins deduced from transcriptomic sequences in the eyestalk and abdominal nerve cord shows a 100% identity grade in almost all sequences (Tables 1–7). We also note that, although some of the transcripts that we identified in the PNC transcriptome were partial sequences, in all cases it was nevertheless possible to identify characteristic conserved domains in the proteins translated. Our results confirm that most molecules of the transduction pathways are common to both retinal and extraretinal photoreceptors, as previously suggested by Gotow and Nishi (2008), and by Kingston and Cronin (2015).

However, we also found five transcripts in the PNC that we could not identify in the eyestalk transcriptome. These differences were in Sets 1, 2, and 3, suggesting some functional peculiarities between retinal and extraretinal photoreceptors. Set 1 (corresponding to the phylogenetic family of the retinoid pathway) contains the first 2 differences. One of these genes is Rdh13, which encodes retinol dehydrogenase 13; in humans, this enzyme participates in retinoid metabolism and oxidizes all-trans-retinol, although it seems to reduce all-trans-retinal with much greater efficiency (Belyaeva et el. 2008). The other gene that we do not identify in the eyestalk is Crabp1, which encodes the cellular retinoic acid-binding protein 1-like protein, which may regulate the access of retinoic acid to the nuclear retinoic acid receptors.

The second group of genes listed in Table 2 contains several genes associated with development processes; among these are several transcriptional regulators. In this phylogenetic family, the *En* and *Hh* genes were not found in the eyestalk; these encode the proteins homeobox protein engrailed-1 and hedgehog protein, respectively. These transcription factors are involved in the development, survival, and differentiation of neuronal photoreceptors (Altieri et al. 2016; Li et al. 2016).

Interestingly, in the same set, we identified the expression of the Pph gene in the PNC. The protein encoded by this gene is the putative retinal homeobox protein Rx2, which plays a critical role in eye formation by regulating the initial specification of retinal cells. This transcription factor is necessary for mushroom body development in the *Drosophila* brain and is conserved between vertebrates and flies (Kraft et al. 2016).

Generally, the rhabdomeric photoreceptors are associated with invertebrate eyes; functional Set 3 corresponds to elements of rhabdomeric phototransduction. From the identified transcripts, we can identify the 1-phosphatidylinositol 4,5-bisphosphate phosphodiesterase delta-4-like protein (plcd4), which hydrolyzes phosphatidylinositol 4,5-bisphosphate (PIP2) to generate two second messenger molecules: DAG and inositol 1,4,5-trisphosphate (IP3). This confirms a previous study by Krusewzka and Larimer (1993).

In this set, we have also identified four different transcripts that encode the α subunits of the heterotrimeric G proteins. These proteins can be identified by their α subunits, and they are grouped into four families based on their sequences and functionality. The four G-protein families are Gαs, Gαi, Gαq, and Gα_12_ (Syrovatkina et al. 2016). In the PNC, we have identified members of three of these families: from the Gαi family, we identified two members (Gαi and Gαo); the other two were Gαs and Gαq. Previous research has established that the Gαs and Gαi families of G proteins may regulate adenylyl cyclases, leading to increased or reduced intracellular levels of cAMP, respectively; another research also shows that the Gαs subunit and cAMP participates in phototransduction in jellyfish (Koyanagi et al. 2008). In the simple photoreceptors (Ip-2 or Ip-1) of the abdominal ganglion of *Onchidiumverruculatum*, phototransduction is triggered by a Go-type protein coupled to guanylate cyclase. This cGMP cascade contrasts with the phototransduction cGMP cascade mediated by the Gt-type G protein coupled to phosphodiesterase in vertebrate photoreceptors (Gotow and Nishi 2008). The heterotrimeric G protein also contains Gβϒ subunits, although we only identified a sequence of the Gβ5 subunit that is generally expressed in the brain (Syrovatkina et al. 2016). It would be interesting to study whether the various G proteins identified are probably those that facilitate the various photoresponsive characteristics of CPRs. As we have previously noted, the extraretinal photoreceptor presents spontaneous activity, as well as a rhythmic and differential photoresponse to monochromatic stimulation of blue and green light. Importantly, these photoreceptors are also modulated by serotonin and dopamine and are coupled to G proteins (Welsh 1934; Rodríguez-Sosa et al. 2003, 2006, 2007, 2011; Pacheco-Ortiz et al. 2018; Sánchez-Hernández et al. 2018).

**Figure. 1.**
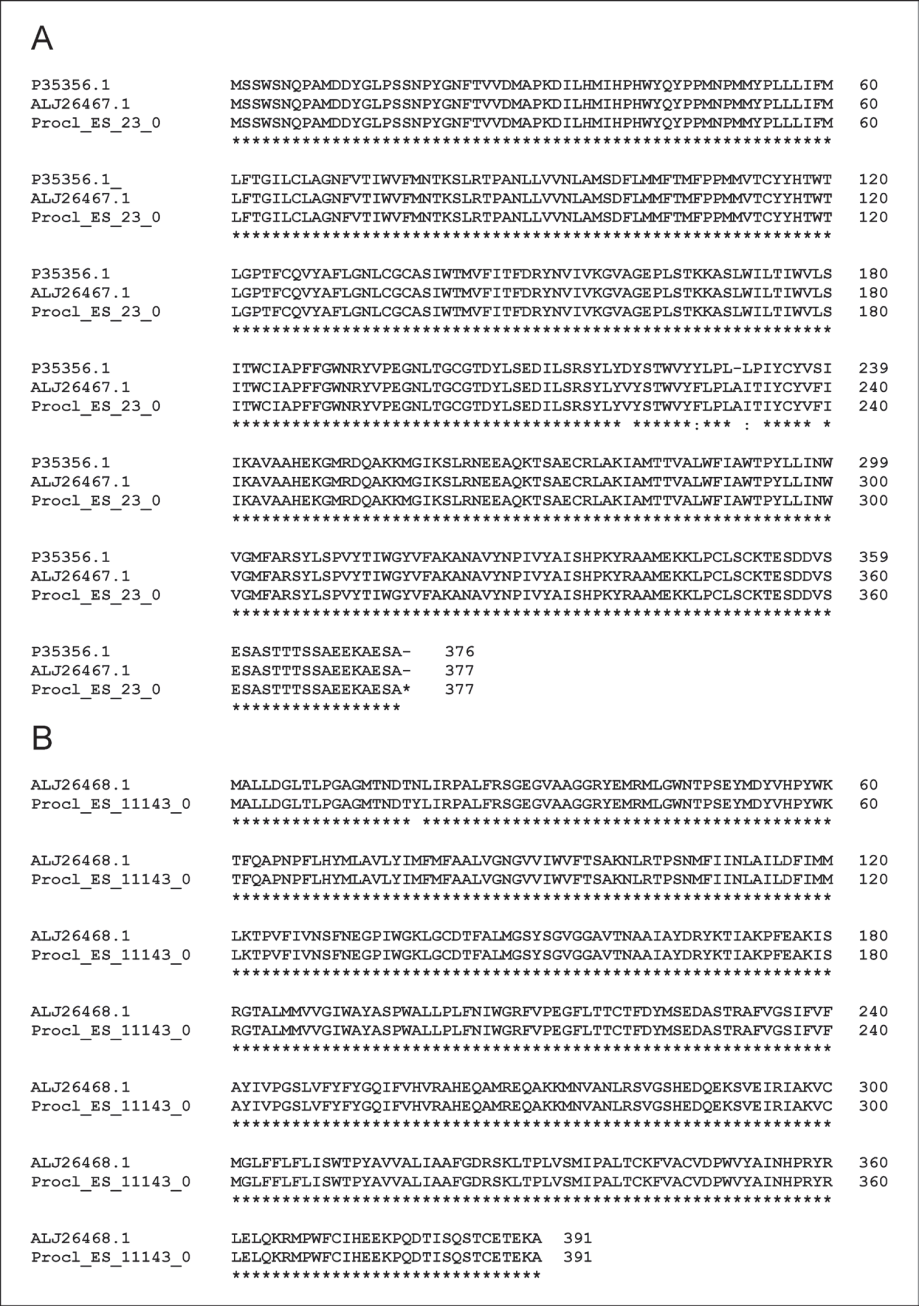
Comparative alignments of opsins reported in the crayfish *Procambarusclarkii***A** long-wavelength-sensitive opsin (UniProtKB/Swiss-Prot: P35356.1; Hariyama et al. 1993); (GenBank: ALJ26467 1; Kingston and Cronin 2015); (Procl_ES_23_0; Manfrin et al. 2015) **B** short-wavelength-sensitive opsin (GenBank: ALJ26468.1; Kingston and Cronin 2015); (Procl_ES_11143_0; Manfrin et al. 2015)

Notably, we did not identify any of the two opsins previously reported in both the eyestalk and the PNC of this species (Kingston and Cronin 2015), although it is possible that the PIA did not find a sufficient level of similarity to the sequences of the phylogenetic families that it uses for identification. However, in the eyestalk transcriptome reported by Manfrin et al. (2015), these two opsins are expressed. The sequence of the long-wavelength-sensitive opsin in the crayfish *P.clarkii* has been reported in three different studies (Hariyama et al. 1993; Kingston and Cronin 2015; Manfrin et al. 2015). A comparison between these sequences shows a similarity of 98–100% (Figure 1A). The short-wavelength-sensitive opsin recently reported in the eyestalk and PNC by Kingston and Cronin (2015) is also found in the list of transcriptomes identified in the eyestalk (Manfrin et al. 2015)^[20]^. These two sequences are 100% identical (Figure 1B).

While the eyestalk transcriptome contains 19 sequences identified or related to the protein beta-arrestin, none were like the beta-arrestin-1 identified in the PNC. This protein participates in the deactivation of the ciliary and rhabdomeric cascades and is regenerated by retinal binding proteins (Peterson et al. 2017). This particularity merits further exploration in future studies, since beta-arrestin-1 may be a determining element in the characteristics of retinal and extraretinal photoresponsiveness in this crustacean.

Because ciliary photoreceptors are generally associated with vertebrate eyes, we did not expect to identify genes of both phototransduction cascades in this structure with simple photoreceptors. This finding suggests that these light-mediated biochemical processes are highly conserved and coexist in various invertebrate species, as previous studies have shown (Arendt et al. 2004; Gotow and Nishi 2008; Verasztó et al. 2018).

The physical appearance of the nervous tissue in the crayfish is of a whitish color; the presence of numerous enzymes that participate in the synthesis pathways of various pigments is remarkable. The pigment expression in this structure suggests that the pigments are associated with various functions. For example, one of the functional gene sets is related to the melanin synthesis pathway; melanin is a unique pigment with several functions and is found in all biological kingdoms (Eisenman and Casadevall 2012). It plays a major role in skin homeostasis by conferring photoprotection and is also involved in neutralizing free radicals and reactive oxygen species, promoting fitness and cell survival, and encapsulating harmful metabolites; it is synthesized in response to microbial infections in invertebrates (Casadevall et al. 2017; Maranduca et al. 2019; Zhang et al. 2019).

Similarly, pterin is a member of the group of compounds called pteridines. Some microorganisms utilize cyanide and heavy metals for the efficient production of pterin compounds, and the antimicrobial activity of pterin has been studied and substantiated by antagonistic activity against *Escherichiacoli* and *Pseudomonasaeruginosa*. Furthermore, the pterin compound has been proven to inhibit the formation of biofilm. The extracted pterin compounds may function as antioxidants or antimicrobials (Mahendran et al. 2018) in various organisms such as *P.clarkii.*

We also identify four enzymes that participate in the biosynthesis of the heme group, a cofactor involved in multiple cellular processes. One of the best known of these is the binding of oxygen to hemoglobin and myoglobin, although it has also been established that heme can interact with transcription factors that regulate genes participating in the maintenance of circadian rhythms (Carter et al. 2017; Martins et al. 2019).

In the PNC transcriptome, we have identified two transcripts that encode proteins involved in diurnal rhythms (Table 6). The gene *lark* encodes an RNA-binding protein that may be required in *Drosophila* for circadian repression of eclosion (www.uniprot.org/uniprot/Q94901), as well as for the calcium-activated potassium channel *slowpoke* (GenBank: Q03720 and AAA28902.1). A study on *Drosophila* has recently reported that this potassium channel functions in central clock cells, in addition to multiple components of the circadian circuits; these authors suggest that it contributes to generating rhythms of daily neuronal activity and facilitates the propagation of circadian information through output circuits (Ruiz et al. 2021). While Sullivan et al. (2009) report that the CPRs do not originate the circadian rhythm from the locomotor activity in crayfish, the CPRs are essential for maintaining synchronization of this circadian rhythmicity in crayfish within the 24-h light-dark cycle (Rodríguez-Sosa et al. 2012). It would be interesting to study the participation of this calcium-activated potassium channel in the expression of the circadian spontaneous response of the CPRs in the PNC (Rodríguez-Sosa et al. 2008).

Finally, crystallins are proteins that contribute to the transparency and refractive index of the lens in vertebrates. However, their expression in the PNC is probably associated with other functions that have been described for crystallins outside of the lens; primarily, they have been linked to protective functions against some stressors and the maintenance of cytoplasmic order (Tomarev and Piatygorsky 1996; Slingsby and Wistow 2014).

Although retinal and extraretinal photoreceptors in crayfish show significant morphological differences regarding structure, the phototransduction pathways at the molecular level have common pathways, as we show in this study. Interestingly, these very different cell types share molecular components of photoreception and other associated metabolic pathways.

We believe that the knowledge of the molecular components involved in the phototransduction of the caudal photoreceptors and other associated metabolic pathways which we present in this study can serve as an essential primary resource for future research while also facilitating the comparative analysis of photoreception processes with other species of decapod crustaceans.

## Conclusions

Unlike the image-forming function in the eyes, extraretinal photoreception has not been deeply studied, particularly at the molecular level. In this study, we have described 62 transcripts from the PNC of the crayfish *Procambarusclarkii*, using a bioinformatics tool that identifies phylogenetic families of light-interacting transcripts. We compared these results to the crayfish eyestalk transcriptome described by other researchers (Kingston and Cronin 2015; Manfrin et al. 2015), finding that the high similarity in both transcriptomic sequences structures suggests that extraretinal and retinal photoreceptors share common mechanisms of phototransduction.

The molecular components described here potentially underlie photoreceptor development, pigment synthesis, phototransduction, and the regulation of circadian rhythm from the pleonal nerve cord of this species. We identify 5 transcripts that are expressed only in the transcriptome of the PNC. Furthermore, phototransduction in the extraretinal photoreceptors presents differences that merit further elucidation in future studies.

All these sequences are available in the GenBank database. We hope that the availability of these sequences will facilitate access for other researchers performing molecular-level studies and comparative analyses on these processes in future studies on decapod crustaceans.

## Supplementary materials

Supplementary material 1 Appendix S1, S2 Data type Text Explanation note We have additional supporting information available online in the supporting tab for this article. **Appendix S1.** Some sequence alignments allow us to appreciate the degree of similarity among *Drosophilamelanogaster*, the crayfish (*P.clarkii*) pleonal nerve cord, and the eyestalk. **Appendix S2.** Nucleotide sequence list referred from Tables 1–7. The supplementary materials are availible also from Dryad (https://doi.org/10.5061/dryad.pg4f4qrqp). https://binary.pensoft.net/file/612826This dataset is made available under the Open Database License (http://opendatacommons.org/licenses/odbl/1.0/). The Open Database License (ODbL) is a license agreement intended to allow users to freely share, modify, and use this Dataset while maintaining this same freedom for others, provided that the original source and author(s) are credited.Gabina Calderón-Rosete, Juan Antonio González-Barrios, Celia Piña-Leyva, Hayde Nallely Moreno-Sandoval, Manuel Lara-Lozano, Leonardo Rodríguez-Sosa
