# Supplementary material for: Transcriptional identification of genes light-interacting in the extraretinal photoreceptors of the crayfish Procambarusclarkii
Source: Zookeys. 2021 Nov 19;1072:107–27. doi: 10.3897/zookeys.1072.73075 (PMC8626408; doi:10.3897/zookeys.1072.73075)
Supplement: Supplementary material 1 — Appendix S1, S2 [file zookeys-1072-107-s001.docx]

**APPENDIX S1: Some sequence alignments allow us to appreciate the degree of similarity among *Drosophila melanogaster*, the crayfish (*P. clarkii*) abdominal nerve cord, and the eyestalk.**

Calderón-Rosete et al., 2021.

AAA28902.1 calcium-activated K+ channel subunit, partial [Drosophila melanogaster]

QIA97593.1 calcium-activated potassium channel transcript variant 4, partial [Procambarus clarkii]

Alignment statistics for match #1 Score Expect Method Identities Positives Gaps

513 bits(1320) 8e-179 Compositional matrix adjust. 241/263(92%) 253/263(96%) 0/263(0%)

QIA97593.1 1 MSTVGYGDVYCHTVFGRTFLVFFLLVGLAIFASCIPEIIDLVGTRSKYGGTLKNERGRRH 60

MSTVGYGDVYC TV GRTFLVFFLLVGLA+FAS IPEII+LVG+ +KYGG LK E G+RH

AAA28902.1 283 MSTVGYGDVYCETVLGRTFLVFFLLVGLAMFASSIPEIIELVGSGNKYGGELKREHGKRH 342

QIA97593.1 61 IVVCGHITYESVSHFLKDFLHEDREDVDVEVVFLHRKPPDLELEGLFKRHFTTVEFFQGS 120

IVVCGHITYESVSHFLKDFLHEDREDVDVEVVFLHRKPPDLELEGLFKRHFTTVEFFQG+

AAA28902.1 343 IVVCGHITYESVSHFLKDFLHEDREDVDVEVVFLHRKPPDLELEGLFKRHFTTVEFFQGT 402

QIA97593.1 121 IMSPIDLQRVKVHEADACLVLANKYCQDPDAEDAANIMRVISIKNYSDDIRVIIQLMQYH 180

IM+PIDLQRVKVHEADACLVLANKYCQDPDAEDAANIMRVISIKNYSDDIRVIIQLMQYH

AAA28902.1 403 IMNPIDLQRVKVHEADACLVLANKYCQDPDAEDAANIMRVISIKNYSDDIRVIIQLMQYH 462

QIA97593.1 181 NKAYLLNIPSWDWKRGDDVICLAELKLGFIAQSCLAPGFSTMMANLFAMRSYKTSPDMQA 240

NKAYLLNIPSWDWK+GDDVICLAELKLGFIAQSCLAPGFSTMMANLFAMRS+KTSPDMQ+

AAA28902.1 463 NKAYLLNIPSWDWKQGDDVICLAELKLGFIAQSCLAPGFSTMMANLFAMRSFKTSPDMQS 522

QIA97593.1 241 WQNDYLCGTGCEMYTETLSPSFV 263

W NDYL GTG EMYTETLSP+F+

AAA28902.1 523 WTNDYLRGTGMEMYTETLSPTFI 545

QIA97593.1 calcium-activated potassium channel transcript variant 4, partial [Procambarus clarkii]

Procl_ES_4724_0_eyestalk Full=Calcium-activated potassium channel [Procambarus clarkii]

Q03720.3|SLO_DROME RecName: Full=Calcium-activated potassium channel slowpoke; Short=dSlo; AltName: Full=BK channel; AltName: Full=Maxi K channel; Short=MaxiK

AAA28902.1 calcium-activated K+ channel subunit, partial [Drosophila melanogaster]

QIA97593.1 ------------------------------------------------------------ 0

Procl_ES_4724_0 ----------------------------MSDDSGPAHLSQTECLKVRKWWCFLLSSIFTF 32

sp|Q03720.3|SLO_DROME MASGLIDTNFSSTLANGMSGCDQSTVESLADDPTDSPFDADDCLKVRKYWCFLLSSIFTF 60

AAA28902.1 ----------------GMSGCDQSTVESLADDPTDSPFDADDCLKVRKYWCFLLSSIFTF 44

QIA97593.1 ------------------------------------------------------------ 0

Procl_ES_4724_0 LAGIFIVLIWRVFSFLCCRNRDPSEYQKQQEKDKLLAQQGKPPGQPKPKNLMEGNFVTEA 92

sp|Q03720.3|SLO_DROME LAGLLVVLLWRAFAFVCCRKEPDLGPNDPKQK---------EQKASRNKQEFEGTFMTEA 111

AAA28902.1 LAGLLVVLLWRAFAFVCCRKEPDLGPNDPKQK---------EQKASRNKQEFEGTFMTEA 95

QIA97593.1 ------------------------------------------------------------ 0

Procl_ES_4724_0 KDWAGELISGQTTTGRILVVLVFILSIASLVIYFIDASNIMEDGVEHCQPWSANTTQQID 152

sp|Q03720.3|SLO_DROME KDWAGELISGQTTTGRILVVLVFILSIASLIIYFVDASS---EEVERCQKWSNNITQQID 168

AAA28902.1 KDWAGELISGQTTTGRILVVLVFILSIASLIIYFVDASS---EEVERCQKWSNNITQQID 152

QIA97593.1 ------------------------------------------------------------ 0

Procl_ES_4724_0 LAFNIFFMVYFFIRFIAASDKLWFMLEMYSFVDYFTIPPSFVSIYLDRTWIGLRFLRALR 212

sp|Q03720.3|SLO_DROME LAFNIFFMVYFFIRFIAASDKLWFMLEMYSFVDYFTIPPSFVSIYLDRTWIGLRFLRALR 228

AAA28902.1 LAFNIFFMVYFFIRFIAASDKLWFMLEMYSFVDYFTIPPSFVSIYLDRTWIGLRFLRALR 212

QIA97593.1 ------------------------------------------------------------ 0

Procl_ES_4724_0 LMSVPDILQYLNVLKTSSSIRLAQLCSIFIAVWLTGAGIIHLLENSGDPLDFSNAHPLSY 272

sp|Q03720.3|SLO_DROME LMTVPDILQYLNVLKTSSSIRLAQLVSIFISVWLTAAGIIHLLENSGDPLDFNNAHRLSY 288

AAA28902.1 LMTVPDILQYLNVLKTSSSIRLAQLVSIFISVWLTAAGIIHLLENSGDPLDFDNAHRLSY 272

QIA97593.1 ----------MSTVGYGDVYCHTVFGRTFLVFFLLVGLAIFASCIPEIIDLVGTRSKYGG 50

Procl_ES_4724_0 WTCVYFLIVTMSTVGYGDVYCHTVFGRTFLVFFLLVGLAIFASCIPEIIDLVGTRSKYGG 332

sp|Q03720.3|SLO_DROME WTCVYFLIVTMSTVGYGDVYCETVLGRTFLVFFLLVGLAMFASSIPEIIELVGSGNKYGG 348

AAA28902.1 WTCVYFLIVTMSTVGYGDVYCETVLGRTFLVFFLLVGLAMFASSIPEIIELVGSGNKYGG 332

***********.**:**************:***.*****:***: .****

QIA97593.1 TLKNERGRRHIVVCGHITYESVSHFLKDFLHEDREDVDVEVVFLHRKPPDLELEGLFKRH 110

Procl_ES_4724_0 TLKNERGRRHIVVCGHITYESVSHFLKDFLHEDREDVDVEVVFLHRKPPDLELEGLFKRH 392

sp|Q03720.3|SLO_DROME ELKREHGKRHIVVCGHITYESVSHFLKDFLHEDREDVDVEVVFLHRKPPDLELEGLFKRH 408

AAA28902.1 ELKREHGKRHIVVCGHITYESVSHFLKDFLHEDREDVDVEVVFLHRKPPDLELEGLFKRH 392

**.*:*:****************************************************

QIA97593.1 FTTVEFFQGSIMSPIDLQRVKVHEADACLVLANKYCQDPDAEDAANIMRVISIKNYSDDI 170

Procl_ES_4724_0 FTTVEFFQGSIMSPIDLQRVKVHEADACLVLANKYCQDPDAEDAANIMRVISIKNYSDDI 452

sp|Q03720.3|SLO_DROME FTTVEFFQGTIMNPIDLQRVKVHEADACLVLANKYCQDPDAEDAANIMRVISIKNYSDDI 468

AAA28902.1 FTTVEFFQGTIMNPIDLQRVKVHEADACLVLANKYCQDPDAEDAANIMRVISIKNYSDDI 452

*********:**.***********************************************

QIA97593.1 RVIIQLMQYHNKAYLLNIPSWDWKRGDDVICLAELKLGFIAQSCLAPGFSTMMANLFAMR 230

Procl_ES_4724_0 RVIIQLMQYHNKAYLLNIPSWDWKRGDDVICLAELKLGFIAQSCLAPGFSTMMANLFAMR 512

sp|Q03720.3|SLO_DROME RVIIQLMQYHNKAYLLNIPSWDWKQGDDVICLAELKLGFIAQSCLAPGFSTMMANLFAMR 528

AAA28902.1 RVIIQLMQYHNKAYLLNIPSWDWKQGDDVICLAELKLGFIAQSCLAPGFSTMMANLFAMR 512

************************:***********************************

QIA97593.1 SYKTSPDMQAWQNDYLCGTGCEMYTETLSPSFV--------------------------- 263

Procl_ES_4724_0 SYKTSPDMQAWQNDYLCGTGCEMYTETLSPSFVGMTFPQASELCFSKLKLLLLAIEVKN- 571

sp|Q03720.3|SLO_DROME SFKTSPDMQSWTNDYLRGTGMEMYTETLSPTFIGIPFAQATELCFSKLKLLLLAIEIKGA 588

AAA28902.1 SFKTSPDMQSWTNDYLRGTGMEMYTETLSPTFIGIPFAQATELCFSKLKLLLLAIEIKGA 572

*:*******:* **** *** *********:*:

QIA97593.1 ------------------------------------------------------------ 263

Procl_ES_4724_0 EEGTDSKIAINPKATKIQPNTQGFFIAQSADEVKRAWYYCKACHDDIKDETLIKKCKCKN 631

sp|Q03720.3|SLO_DROME EEGADSKISINPRGAKIQANTQGFFIAQSADEVKRAWFYCKACHEDIKDETLIKKCKCKN 648

AAA28902.1 EEGADSKISINPRGAKIQANTQGFFIAQSADEVKRAWFYCKACHEDIKDETLIKKCKCKN 632

QIA97593.1 ------------------------------------------------------------ 263

Procl_ES_4724_0 YKSDI-------ETELYQVTYTPPELPKRLLNNSRG--DKIPVR-------DGIANQNS- 674

sp|Q03720.3|SLO_DROME LTVQPRSKFDDLDEHHPAPTFTPPELPKRVHVRGSVSGDITRDREDTNLLNRNVRRPNGT 708

AAA28902.1 LTVQPRSKFDDLDEHHPAPTFTPPELPKRVHVRGSVSGDITRDREDTNLLNRNVRRPNGT 692

QIA97593.1 ------------------------------------------------------------ 263

Procl_ES_4724_0 ----SGQPLVNAAKTLAAA------KKNGGRPADALTSPSQGYNRSVPQQDRPTSRSSGG 724

sp|Q03720.3|SLO_DROME GNGTGGMHHMNNTAAAAAAAAAAGKQVNKVKPTVNVSRQVEGQVISPSQYNRPTSRSSGT 768

AAA28902.1 GNGTGGMHHMNNTAAAAAAAAAAGKQVNKVKPTVNVSRQVEGQVISPSQYNRPTSRSSGT 752

QIA97593.1 ------------------------------------------------------------ 263

Procl_ES_4724_0 GNGGNNNG--LTVGIADDQAKDFDFEKTEMKYDSTGMFHWCPARSLEDCILDRNQAAMTV 782

sp|Q03720.3|SLO_DROME GTQNQNGGVSLPAGIADDQSKDFDFEKTEMKYDSTGMFHWSPAKSLEDCILDRNQAAMTV 828

AAA28902.1 GTQNQNGGVSLPAGIADDQSKDFDFEKTEMKYDSTGMFHWSPAKSLEDCILDRNQAAMTV 812

QIA97593.1 ------------------------------------------------------------ 263

Procl_ES_4724_0 LNGHVVVCLFADPDSPLIGLRNLVMPLRASNFHYHELKHVVIVGSVDYIRREWKMLQNLP 842

sp|Q03720.3|SLO_DROME LNGHVVVCLFADPDSPLIGLRNLVMPLRASNFHYHELKHVVIVGSVDYIRREWKMLQNLP 888

AAA28902.1 LNGHVVVCLFADPDSPLIGLRNLVMPLRASNFHYHELKHVVIVGSVDYIRREWKMLQNLP 872

QIA97593.1 ------------------------------------------------------------ 263

Procl_ES_4724_0 KISVLNGSPLSRADLRAVNVNLCDMCVILSAKVPSNDDPTLADKEAILASLNIKAMTFDD 902

sp|Q03720.3|SLO_DROME KISVLNGSPLSRADLRAVNVNLCDMCCILSAKVPSNDDPTLADKEAILASLNIKAMTFDD 948

AAA28902.1 KISVLNGSPLSRADLRAVNVNLCDMCCILSAKVPSNDDPTLADKEAILASLNIKAMTFDD 932

QIA97593.1 ------------------------------------------------------------ 263

Procl_ES_4724_0 TIGVLNQNRTTSDLTCGGHDYGLPDLTLTDPSGGGDTLSPLGSPIVLQRRGSVYGANVPM 962

sp|Q03720.3|SLO_DROME TIGVLSQRGPEFD----------------------NLSATAGSPIVLQRRGSVYGANVPM 986

AAA28902.1 TIGVLSQRGPEFD----------------------NLSATAGSPIVLQRRGSVYGANVPM 970

QIA97593.1 ------------------------------------------------------------ 263

Procl_ES_4724_0 ITELINDSNVQFLDQDDDDDPDTELYLTQPFACGTAFAVSVLDSLMSTTYFNQNALTLIR 1022

sp|Q03720.3|SLO_DROME ITELVNDSNVQFLDQDDDDDPDTELYLTQPFACGTAFAVSVLDSLMSTTYFNQNALTLIR 1046

AAA28902.1 ITELVNDGNVQFLDQDDDDDPDTELYLTQPFACGTAFAVSVLDSLMSTTYFNQNALTLIR 1030

QIA97593.1 ------------------------------------------------------------ 263

Procl_ES_4724_0 SLITGGATPELELILAEGAGLRGGYSTPETLANRDRCQVGQISLYDGPLGQFGEGGKYGD 1082

sp|Q03720.3|SLO_DROME SLITGGATPELELILAEGAGLRGGYSTVESLSNRDRCRVGQISLYDGPLAQFGECGKYGD 1106

AAA28902.1 SLITGGATPELELILAEGAGLRGGYSTVESLSNRDRCRVGQISLYDGPLAQFGECGKYGD 1090

QIA97593.1 ------------------------------------------------------------ 263

Procl_ES_4724_0 LFCAALRNYGMLCIGLYRTLRMVPQSAVGLDCRFRDTSSSCDASSKRYVITNPPDDFTLL 1142

sp|Q03720.3|SLO_DROME LFVAALKSYGMLCIGLYR---------------FRDTSSSCDASSKRYVITNPPDDFSLL 1151

AAA28902.1 LFVAALKSYGMLCIGLYR---------------FRDTSSSCDASSKRYVITNPPDDFSLL 1135

QIA97593.1 ------------------------------------------------- 263

Procl_ES_4724_0 PTDQVFVLMQFDPGLEYKPNRGDMTKEDNS------------------- 1172

sp|Q03720.3|SLO_DROME PTDQVFVLMQFDPGLEYKPPAVRAPAGGRGTNTQGSGVGGGGSNKDDNS 1200

AAA28902.1 PTDQVFVLMQFDPGLEYKPPAVRAPAGGRGTNTQGSGVGGGGSNKDDNS 1184

Q94901.1|LARK_DROME RecName: Full=RNA-binding protein lark

QIA97594.1 RNA-binding protein lark-like protein, partial [Procambarus clarkii]

Procl_ES_2543_0_EYESTALK (LARK)[Procambarus clarkii]

sp|Q94901.1|LARK_DROME -MPGAGTFKLFIGNLDEKTQATELRALFEKYGTVVECDVVKNYGFVHMETEQQGRDAIQN 59

QIA97594.1 MPVRGNTFKIFVGNLSDRATGSDIRELFEAHGTVVEADVVKNYGFVHMEKEDEGQAAIEA 60

Procl_ES_2543_0_EYESTALK MPVRGNTFKIFVGNLSDRATGSDIRELFEAHGTVVEADVVKNYGFVHMEKEDEGQAAIEA 60

..***:*:***.::: .:::* *** :*****.************.*::*: **:

sp|Q94901.1|LARK_DROME LNGYTLNEFAIKVEAAKSRRAPNTPTTKIFVGNLTDKTRAPEVRELFQKYGTVVECDIVR 119

QIA97594.1 LNGHSIHGKPMVVEASTGARKGGNQKTKIFIGNLHKDSKLEELKSLFEVYGSVVEADILT 120

Procl_ES_2543_0_EYESTALK LNGHSIHGKPMVVEASTGARKGGNQKTKIFIGNLHKDSKLEELKSLFEVYGSVVEADILT 120

***:::: : ***:.. * .. .****:*** ..:: *::.**: **:***.**:

sp|Q94901.1|LARK_DROME NYGFVHLDCVGDVQDAIKELNGRVVDGQPLKVQVSTSRVRPKPGMGDPEQCYRCGRSGHW 179

QIA97594.1 NYAFIHMDDEAQAQRAIRELDGYELHGLRLRVQESTSRVRQQAGMGNPDMCYRCGSGGHW 180

Procl_ES_2543_0_EYESTALK NYAFIHMDDEAQAQRAIRELDGYELHGLRLRVQESTSRVRQQAGMGNPDMCYRCGSGGHW 180

**.*:*:* .:.* **:**:* :.* *:** ****** : ***:*: ***** .***

sp|Q94901.1|LARK_DROME SKECPRLYGSAGGGREPPSPLSAGGYRDRMYGRDPYPPPPPPPPFLRDRIMD---GFRDY 236

QIA97594.1 SKECPRDG-RIGGFRYPDRERGG---RSFGSRYDPYPPPP-PPSYARERMLRYRDDFDRY 235

Procl_ES_2543_0_EYESTALK SKECPRDG-RIGGFRYPDRERGG---RSFGSRYDPYPPPP-PPSYARERMLRYRDDFDRY 235

****** ** * * .. *. ******* ** : *:*:: .* *

sp|Q94901.1|LARK_DROME DYYDRRFEDSRDLYERRYQTSRMRDFPPPP--ISR----REPMPLPPTLSGSLRSCSVSR 290

QIA97594.1 DRYDRYYDE--GLYERR------GDHPPPPPPMLDDLYERRLPPLPPHPD---------- 277

Procl_ES_2543_0_EYESTALK DRYDRYYDE--GLYERR------GDHPPPPPPMLDDLYERRLPPLPPHPD---------- 277

* *** ::: .***** *.**** : *. **** .

sp|Q94901.1|LARK_DROME GYDTMFSRRSPPPPR--SSNGMSRYGSPTPHGYEDFSRDAFDERMISSRGMRGPSPPGRR 348

QIA97594.1 -Y-LRYGRRSPPPRYPPPPPPMRGYGPPDRRPY--------------------------- 308

Procl_ES_2543_0_EYESTALK -Y-LRYGRRSPPPRYPPPPPPMRGYGPPDRRPY*-------------------------- 308

* :.****** * ** * : *

sp|Q94901.1|LARK_DROME YAPY 352

QIA97594.1 ---- 308

Procl_ES_2543_0_EYESTALK ---- 308

_______________________________________________________________________________________________________

Procl_ES_659_0_eyestalk Full=Beta,beta-carotene 15,15'-monooxygenase [Procambarus clarkii]

QPM92663.1 carotenoid oxygenase, partial [Procambarus clarkii]

Procl_ES_11203_0_eyestalk RPE65 Full=Retinol isomerase [Procambarus clarkii]

Procl_ES_4243_0_eyestalk Full=Beta,beta-carotene 15,15'-monooxygenase [Procambarus clarkii]

Procl_ES_30934_0_eyestalk Full=Beta,beta-carotene [Procambarus clarkii]

Procl_ES_1244_0_eyestalk Full=Beta,beta-carotene 15,15'-monooxygenase [Procambarus clarkii]

Procl_ES_659_0 NQDGVYISVTGVDVGEPGFEMPVIN-TAHTGKPYRFVYGTGAYDQGYFKNSVCKMDVESG 442

QPM92663.1 ----------------------RIN-PNFIGKPYRYLYAVRAVPGRLFDA-IVKLDAESK 36

Procl_ES_11203_0 GR----IQVQGHIISKQFFDLPRINY-RHNGKEYTYAYGVDVNPRGIDFPKLVKMNVETG 153

Procl_ES_4243_0 EI----VL-QCEKLTRIPIENPCIN-PKIRSSKHQYIWAMGPDPNGGDSGFVIKLDVTSG 441

Procl_ES_30934_0 NL----VYIKPELLVDIGCEVPRIHYDKYNGRHYQYFYAICSDVDHPCPGTLVKADVVNK 651

Procl_ES_1244_0 GS----LLLTPEVITDYAYEIPTLN-PSYVGKRYRYFYGSSGNMTS-TTGKVGKVDLDSR 442

:: . : : :. : * : .

Procl_ES_659_0 RS-WVWRGNEHQYLSEPSFIPAPD--AIDEDDGVILCSVADVRKG-SPDFLLVVDARTMK 498

QPM92663.1 EQVAIWE-HPCTSPSEPIFVPRPSTNSTDEDDGVVLTVILS-QHE-KKSFLLVLDAKNLK 93

Procl_ES_11203_0 DT-YLWR-EEGKLVSEPVFVAAPD--ASAEEHGVVLSTLIDKNEP-KFVALLVVNPKTWR 208

Procl_ES_4243_0 ET-FMFT-EDKIYCAAPEFVAKPN--AVSEDDGVILLQCVNSQDE-KKTYLLVLDAKNMT 496

Procl_ES_30934_0 TH-LEWS-EDNVYPSEPIFVPSPE--AQREDDGVVLSALLRARGLDQQVCVLVLDASTFT 707

Procl_ES_1244_0 ET-KDWS-EDGLYTSVAYFVPRPG--ATSEDDGVVLVTLLHADDK-TKVTLLVLAAGDMT 497

: . : *: * : *:.**:* :**:

Procl_ES_659_0 ELGRAEVD--ARIPTSLHGVFLPERH*------------------------ 522

QPM92663.1 EIARADLP--IHVPLSF---------------------------------- 108

Procl_ES_11203_0 ELARVEFEAEGAVTSTFHGQFAGANESVYRY*------------------- 239

Procl_ES_4243_0 EICRASVTTTSSVPMPLHGHYIPVIGQ*----------------------- 523

Procl_ES_30934_0 ELGRVEFTAPGPVPKCLHGWWVQEGAFTLSSHHNIKPKKVTKTPIYKGAG* 757

Procl_ES_1244_0 EVARVSFTTPSDVPRSLHGIYIPA*-------------------------- 521

*: *... : :

**APPENDIX S2: Nucleotide sequence list referred from Tables 1-7**

**Calderón-Rosete et al., 2021.**

SET 1. Retinoid Pathway vertebrate and invertebrate

>MN110026 Procambarus clarkii retinal-binding protein-like mRNA, partial CDS

CCCTTCCTTCACGAGGTTACCCTCAAGAAAATAAGAATATTTGGACACAGTGGGTGGAAA

GAGGCTCTATTAGAAGATATTGACGCTGACCAACTGCCTCAACACTGGGGAGGAACCAGA

ACTGACCCTGACGGCAACACCAAATGTCCCTCACAGATATGTTTAGGAGGAGAAGTACCC

AAGAAATATTACCTAAGTCTCAGCAAGAGCAATCTATCCAAGATTACAGATGATGATAAT

CTATCTACTATCACTCTGAACAAAGGTGGCAAAAAGAGACTTAAATATGATGTTAAGCAA

CCTGGATCCCACTTAAAGTGGGAATTTCGTACCGAAGATTTTGATGTGGGATTTGGAGTG

TCCCGCAAAGTGAAAAAAGGCGAAGAGGAGATTTTGGTACCAATGCAGAGAGTTAACTCA

CAGTTGGTGACAGAGGAAGGATATTTAGTGTGCACAGAACCTGGCACATATGTGGTG

>MT601680 Procambarus clarkii retinol dehydrogenase 11-like mRNA, partial CDS

GCAGACTCTGCTGCTGGGAGCAACTTCTCGTTCTTCGCTCCGCTGCTGCCACTCTTAAGA

GGAGTGAGGATGTTGGAGGCGGCAGCGCTGGTGGTGCTGGTGCTGGTGGTCACCATCCGT

GTGGTGTACCGATGGCTCTCCGGCCGCTGCAACTCCTCCACCACGCTAGACGGCAAGACT

GTCATCATCACCGGCGCTTCTGCAGGGATAGGGAAGGAGACGGCCAAGGACCTGGCAGGA

CGTGGGGCGAGGGTCATCATGGCCTGCAGGAACGTGGAGAAGGCTGACAAAGTGGCAACT

GAGATCCGGGTAGCGACCAACTACCGCGGGGAGGTTGAGGTCCGCAGACTTGACACTTCC

GACCTCGCCTCCGTCAGAGAGTTCGCTAGGAAGATCCTCGAAAACGAGAAGTCCCTCCAC

ATCTTGATCAACAACGCTGGGATCATGGGTCCACCCAGACGAGAGGTCACCGCGGACGGC

CTGGAGCTCACCATGGCTACCAACCACTACGGCCACTTCCTCCTCACCAACCTCCTGCTG

GGTCTCCTGAAGAAGAGCGCCCCGAGCCGAATCATCAACGTCACCTCCGACAGCCACGAC

TACGTCAGTAGACTCAACCCGGACAGTCTCAACTTCGAGCGGGACGACTACACCTCCATG

ACGGCTTACGGCCAGAGCAAGCTCTGTAACATCCTCTTCACCCTCGACCTCACCAGCAAG

CTCCAGGGCACAGGTGTGACAGCCAACAGTGTCCATCCCGGGTGTGTGTCCACGGAGATA

TTCTACAAAGGTCAAGTGACCTTATTCGCTTGGGTGTGCGGCAAGCTCTTCTATCTGATG

GGCAAGGACGCCAAGCTTGGAGCTCAACCTGTGATTTACCTGGCAGTGTCGGAGGAGGTC

GAGGACGTCTCCGGTCACTACTTCGTTGACTGCAAGGACACCCCGACGACGGAGCTGGCG

CAGCAGAGGAAGCTGGCGCGGCACCTCTGGGAAGCTAGCGAGGTCGACGTCAAGCTTCAG

CCCCACGAGAGGTTCTACTAA

>MT601681 Procambarus clarkii retinol dehydrogenase 13-like mRNA, partial CDS

ACGGCGGTGGTGGTCTTGGCCATAAGGCTCGTCTACAGGTACCAGTCCGGACGATGTTCC

TCCCACAGGAAACTGGTGGGCAAGACGGTCATTGTCACCGGAGCTTCTGCTGGCATCGGG

AAGGAGGCGGCGCGAGACTTGGCCCGACGAGGAGCTCGGGTCATCCTCGCCTGCAGGAAC

ATCAACAAAGCACAAAAGGTTGCAGATGACATCATGAGGACTACAGGGAACAGGAAGGTG

GTAGTACGTAAGCTGGACACGTCCGACCTGGCCTCCGTCAGGAGGTTTGCGCGAGGCATC

CTTGCTACAGAAACTGCTCTCCATGTCCTGGTGAATAATGCAGGAATATATGGCATGTCG

GAGAAGAAACTGACAGCAGATGGTCTGGAGCTGACAATGGCCACCAACCATTTTGGACAC

TTCCTTCTCACCAATATGCTGCTGGGG

>MT601679 Procambarus clarkii dehydrogenase/reductase SDR family member 4-like mRNA, partial CDS

GACACTACACTCCCCGGGAGGATGGTGGTGGGTTACCGTGGCCTGAGCCTGGCCCTCCTG

CGTGCCTGGCCCTCCAGTGTCAGGAACATGTCCACCCAGGCCAAGCACAGTGCCAAGCTC

CAGGATAAGGTGGCCATTGTCACTGCCTCCACCGACGGGATTGGCTTGGCTATTGCTCGT

CGCTTGGGTGAAGATGGTGCTCACGTCGTGGTCAGCAGCAGAAAGCAAGCCAATGTTGAT

TCAGCTGTAGCAGAACTTGAAGGTTTAGGCTGTTCAGTCTTGGGTCTCACCTGCCATGTT

GCCAAAGATCAAGACAGACAGAACTTAATTTCCAAGACATTGGAGAAGTTTGGAAGCATT

GACATACTTGTTTCCAATGCAGCTGTAAATCCAACAATGGGAGGTGTATTGGACTGCCCA

GAAAGTGTTTGGGACAAGATCTTTGAGGTGAATGTGAAAAATGCACTCCAGCTTACACAG

CTAGTTGTACCCCACATGCAGAAGCAAGGAGGAGGTGGAGCTGTAGTTTACATCTCATCA

ATTGCTGGATTTCAGCCTATGAATATGCTGGGAGCATATAGTGTAAGCAAAACAGCCCTC

CTTGGGCTAACAAAAGCTGTGGCACAACAAGTTGCCTTTGACAATATTCGAGTCAACTGT

GTTGCCCCTGGTATTGTGAAAACCAATTTCTCAAGTGTGATAACACAACACCCAGCAGTG

TATGAAAAGATTTTGGAATCAATTCCTCTTGGAAGGGTTGCAGACCCGAAGGAGCTGGGA

GGCATAGTGTCTTTCTTGTGTAGCAGCGATGCATCATACATCACTGGCGAAACGTTTGTG

GTTGCAGGTGGCATGCTTTCACGACTATAA

>MT601682 Procambarus clarkii epidermal retinol dehydrogenase 2-like mRNA, partial CDS

TCAAACAAGGGTGATATTGTCACTATTGCCTCTGTGGCTGGTCATGGTGGTGTTAATAAA

CTGGCCGACTACTGTGCCTCTAAGTTTGCTGCTGTTGGCTTTGATGAGAGTCTTCGTCTT

GAGCTGATGGTGGAAGGCTACACTGGGGTCAAGACCACTGTTATTTGTCCGTACTACATC

AGCACGGGAATGTTTGAAGGAGTTAAGTCGAAAGTGATACCAATATTGCAGCCTGAATTT

GTGGCCTCTGAGATTGTTGACGGCATCCTCCTGAACAGAGTCATCGTTGTTTTGCCATCC

TTCTGCCGGATCCTGATCCTCCTCAAATACATCTTGCCCCAGAAGGCCATATATATTTTT

GGGAGA

>MT601683 Procambarus clarkii cellular retinoic acid-binding protein 1-like mRNA, partial CDS

CATCTTGGCAACGCCAACCATACCAAGACCAACCACGTCACCCACCAGAAAGCGACAGTA

GCAGCAGCAGCAGCCCCAGAGCCTCGCCCTGCAGACAGGAGCAGCAACATGGAGCACTTG

GAGGGGAAATACCAACATGAGAGATCCGAAAACTTCGATGAATTCCTCAAGGCCATTGGA

GTCCCGCTGATTCCCCGGAAGCTGATGTTAACGTCGAAGCCAGATGTGGAAGTTGTTCGA

GACGGCGACCGCTGGACGATAAGGATGCTTACGCTAATCAAGACTATCGAGTACGCCTTC

ACCCCGGGAGAAGTCGTCAAGTCGGTGACTATGGGCGGCCTGGCC

>MT601684 Procambarus clarkii carotenoid oxygenase mRNA, partial CDS

CGCATAAATCCAAACTTTATTGGTAAACCCTATCGTTATCTTTATGCTGTTCGTGCTGTC

CCTGGTCGATTATTCGATGCGATTGTCAAACTTGACGCCGAATCAAAAGAACAAGTCGCT

ATTTGGGAACACCCATGCACATCACCTAGTGAACCCATATTCGTGCCTCGACCTTCAACA

AACTCGACGGACGAAGATGATGGAGTCGTTTTAACAGTTATTCTCAGTCAACATGAGAAA

AAATCATTTTTATTAGTTTTAGACGCTAAAAATTTGAAGGAAATCGCTCGAGCTGATTTG

CCTATTCATGTTCCACTTTCATTT

>MT942649 Procambarus clarkii class B scavenger receptor mRNA, partial CDS

TTTGAAAGAAATATTTCGGTTGGATCTGAAGATGACATAATCACTACACTAAATGTCCCA

ATGTTGAGTGCAGTATCTCAATGGAGATTTGCCCAGAGGTTGGCTAAATTGGCCCTCTCG

TCGATGCTGGAAGTATTGAATGAAAAACCTTTTGTCTCCAAGTCTGTCCGTGACTTGATG

TGGGGCTACGATGATCCACTCCTGAGAATAGCAAAGGATATTATTCCACCAGACCAACGA

ATGCCTTATGACAAGTTTGGGTTCTTCATTGAGAAAAATGGATCCACTGATGGTCTGTTC

AATGTTTTCACTGGAGTAAATGATATGACAAAG

_______________________________________________________________________________________________________

SET 2. Photoreceptor specification and retinal determination network

>KY974273 Procambarus clarkii Tyrosine-protein kinase Fer (FER) mRNA, partial cds

GGGTCGGTGATGGGGTTCAGCGCGTCCCTGCAGGGAGAGCGGAGCCACGCGGCGCTGCTGGCCCGTCAGG

ACGCAGAGCTGCGCCTGCTGGACACCATGCGGCGGGTGCTGGTGGCCAGGGCCAAGTGCGACAGGGACTA

CGCTGCTGCCCTCACCCACCTGGCCCACACCGCCGCCAAGATGGACGCCCCGGACAACGTTCTCGACGAC

TCCCATCTGCATAAGGCATGGCGCATCATGGTGGAGGGGCTGGACGAGTGGAGCAGCATCATGCGCCAGA

ATGCCGACACCCTGGTGCTGGACACGGTGGAGAAGCTGGCCGCCCTCATCACCGAGAAGCGTGCCTCCAG

GAAGGTCTACTATGAGGAGCACCAGAGGATCACCAATGAAGTAACTAGATTACAAGAAGCTGTTGGAAAA

TCCAAGAATAATTACGAACAGGCACTGGAATTTTACAAGACCTCTAAGACGAAATACGAAGATCAATTTC

TAAAAGGAAAACCAGGACGCAAGTTGGATGAGCTGAAGGAACGGTATCAAAAGGCATGTAAAAAGCTTCA

CCAAGTTCACAATGACTATGTCCTGTTGCTGTGTGAAGCTGCAGACTATGAGAGAGACTTCCGAACAGTA

CTTCTCCCGGGTCTACTCGAGTATCAAGAGCGAGTACAGGAAGATATGATTGACAAATGGCGTTCAATAC

TGACGGAAGTGTGCGAGTTGACAGACACAACACAAGGTCGGTATGCCCAGTTACAAACAGAAGTTGCATC

TTCTGTGTCAGCCATTTCGCCTAAGTCTGAATACTCGTCCTTCTCAGAGACCAATAAGAGTTCCCCGCCC

GACCCAGTATCCTTTGAATTCTGCAGTGAGCTTTTAGGAGATGGTGTTGGTTCCCTTCAGGCTGGTCAGC

TGGCCGTTGACTCGCTCACAGTTGACTCCTTACGACTCAGGCTCAGTGACATTGAACACAGGTTGAGAGA

AGTCACAACAGAGCTTCGTGAAAAACAAAACTTATTAAACCAACATGAAACAGAAGTGATTAACATCAAG

AAGAATTCTTTGCAAGATACCAATATTGCATCAAGATTACCGGTGTTGAAGAGAGCAAGTGATGTCCTGC

GCCGTGAACTGAATGAGTTACGGTGCAAGGAGTCGTGGCTTCAACATCAACACAGCCTCATTCACGACCC

ACTTGCAGCACTGGGCTGTGAAGAGGCTCCTCAGCCTTGGGAGGCTGCACAAGGTCAAGTCAATGGTGAC

TCGCACCAACGTTCCGACTCGCTCTCGCTCAAGACGCGCTCAGCTGCTATCAAAGAGCTGCTTCGCAAAC

CCTTCAACCGTAAGACTAGCGCCTCACCTGCATCCACACCACCCACCCCCACCAGAGCCAATACCGAAGA

GAGAACCACAGCACCAGACCAACAGTCTCTCACGGAAATGTCTGCAGAAGGCCAAGCAGAAGTCCATTCA

CAAGCAGTTAATGGTATACCAGAATTGACATATGACCCGGACCGTTGCTTAGAGGATGAACCATGGTTTC

ATGGTGTATTACCACGAGAGGAGGTGGTGCGGTTATTGGTCAATGAGGGCAATTATCTAGTGAGGGAGAC

TACACGTAATGATGAGCAACAAATTGTTCTCTCAGTTTGCTGGGGCACTCACAAGCACTTCATAGTCCAG

ACCACACCTGAGGGTCACTACAGGTTTGAGGGTCCAGCCTTCCCAACCATCCAGGAACTAATCCTTCACC

AGCACAGCTGTGGTCTACCGGTCACAAACAAGTCGGGTGCTATACTTCGCACGCCCATCTTCCGTGAACG

ATGGGAACTAAATAATGATGATGTTGAGCTGAGAGACAAAATAGGCAGAGGTAACTTTGGGGATGTATAC

AAGGCTCGATTAAGGGATTCGGGACTTGAGGTTGCTGTTAAGACATGTAGAGTTACACTTCCAGATGAGC

AAAAGAAGAAATTCTTACAAGAAGGGCGCATCCTCAAGCAATATGACCATCCCAATATAGTCAAATTCAT

TGGTATATGTGTTCAAAAACAGCCAATTATGATTGTGATGGAATTGGTGCCAGGCGGTTCATTACTAAGT

TTCGTGAGGAACCACAAGGGCCAACTGACAGTGAAGCAGATGATGGGAATGTGTTTGGACACTGCCTCTG

GGATGGCTTACCTAGAATCCAAGAATTGCATCCATCGTGACTTGGCTGCTAGAAACTGCCTTGTTGGGCA

CCGCAATATAGTCAAGATCTCTGACTTTGGCATGTCACGGGAGGAAGAGGAATATATAGTCAGTGACGGC

ATGAAACAAATACCCATCAAATGGACTGCGCCAGAAGCTCTCAACTTCGGCAAGTACACTTCCTTGTGTG

ATGTCTGGAGTTATGGTGTTCTGTGCTGGGAGATCTTCTCCTCGGGCGAAGTTCCTTATCATGGCTACTC

CAATACTAAAGCCAGGGAGATGATTGATTCGGGATACCGGATGCTGGCGCCTCCCAGCACCCCAGAAGAG

ATGTATCAGCTGATGCTAAAGTGCTGGCAGTATGATCCAGAGAACAGACCACACTTTCCAGAAATATATG

CATCTGTTGACAATATATACACGTCACTA

>MN110016 Procambarus clarkii putative retinal homeobox protein Rx2-like mRNA, partial CDS

ATGTTGGGTCTCCCTGCTGACTTCATGGTGGCTCCTTCCCTCGCCCCTGCCCACAGGGAC

TACCAGGAGGGCCTCGCTCAGCCCACGGCCCACAACGCGCCCGTGCCCCACACCCACGCC

AACACGCACCCCAACACGCCCACGCCCCCACAGCAATCGCATTACAGCGCGCAAGCACAG

CACGCCCTCACCAATCTCAGTGCGCAGAACGCGCACACGCACGTGCAAAATACGCACACT

CACACGCCCACGCACGCGCAAAACGCCCACACACACGTGCAGAGCAACCACACCCAGGCA

CAGCCAACCCACGCCCATGCCCACTCCCACGCCGGAGGATCCCTGGAGCCGCCCCTACCT

GGCCCAGTGGTGGGAGAGAAAAGGAAGTTGGATGATGGGGGACCGTCCTATCCGGGCCAG

CCTACCTCCTCTCCGACTAGCCAGACCCAGCAACCTCCCAGCTCCACAGGAGAACCGTCA

ACTAAGAAGACCGACTCTAAATCTAAGAAATCTTCAGATACTCCTGGAGTGAAGAAAAAG

AAAACGAGGACGACGTTCACAGCATACCAGCTGGAAGAGTTGGAGCGAGCCTTCGAACGC

GCGCCATACCCCGATGTCTTCGCCAGAGAGGAACTGGCGGTAAAATTGAACCTTTCTGAA

TCCCGCGTTCAGGTCTGGTTTCAGAACCGTCGAGCTAAGTGGAGGAAGAGAGAACCACCC

AGGAAGAATTATATACCACCTGGTGGCATGGGTGGCGGCCTGGGGGGCACCTTCAACTCC

CTCAGCACCCTATCCCCCTTCAACTCTGGAGAAGCTTGGAGCTACTCTTCCTCCTACGAC

CCCACCCATCTCAACCTCCTCGGCCCCGCCTCCTACCCATTTTCAGCCAACCACAATCCG

GGATATAGCTACCCTATGCTCTCTCAGCCGATGGGAATCAACGACTCCCTTTTTACGAAC

CCTATCGGCCAGATGCGGGCGGGAGACTTCCAACCGTCGACAGGGATGCGGGATTTCGGG

AACAGCCCTCTCAAGACTTACGATTACCTACAAGAGGTGAAGACGGAGGATTTTCTCCAC

GAGAAGAGGGACGCCAACATGAACAGCGTCCGCCACCAGCCGGCCCCAAAGGAAAACAAG

GATTCTTCCTACATCACGCTGCCTTCTTTTTTAAGC

>MN110021 Procambarus clarkii Krueppel homolog 1-like mRNA, CDS

ATGGCCATGATGCCAGGCGGCCTCGACGCCGCTCCTTATCCACCAGCTCCCATGATAATG

GATGTGGATCAGGGCTTTTCCCACGTGGCCCACCATCTCCCTGGACCAATGCAGCCGCCT

CCACAGCCCCTGCAGCAGGCCCAGTACCCATCACCACCTATGCTCCAGAACCACAACGTT

GAAAAACAAGGCCCCTCCGAGCAGATGATGGCTGGAGCAGAACCACCTTACCAGTGTAAG

ATTTGTGGTAAAGGTTTCGCCATCCCAGCTAGGCTAGCGCGCCATCACCGCGTTCACACC

GGAGAGAAACCTTTCAAATGCGAGTTCTGTGAGAAGACGTTTAGTGTGAAGGAGAATTTG

AATGTACATCGTCGTATCCACACCAAGGAACGTCCTTACAAGTGCAATATCTGTGACCGA

TCCTTCGAACACTCTGGCAAGCTGCATCGACACATGCGCACTCACACTGGAGAGAGACCT

CACAAGTGCGAGGTGTGTGGTAAGACCTTCGTTCAGTCCGGACAACTTGTGATTCACATG

CGAGCCCACACCGGTGAAAAGCCATACACCTGCGAATACTGCCAAAAGGGATTCACATGC

TCCAAGCAACTCAAAGTACACATCCGTACACACACAGGTGAAAAGCCCTACGAGTGTGAC

GTCTGCGGCAAGACCTTCGGATACAACCATGTACTCAAGATGCACAAAATGTCCCATCTT

GGCGAGAAGCTGTACAAATGTACCTTATGTGAGGAGTTCTTCAACTCTCGCAAGGCTCTC

GATCGTCACATCCGTGATCACGACAACCCAGAGAGGACATCACGCAAGCAACAGCCACAG

CCAACACAACAACAAGATTCACAATGGAAGTTCTCGCCTCTTTCAAGTAATTCAGACGTC

AAGGAAGTCAAGGACACCAACACCTCCAACTCTCCCATCACCAACTCCTCACCAGCCTCC

ATATCATGGGACAGTGATTCTATGGATCAACAGCTGCCATTTCGTGAGTTGCGACGGGCC

TCTGAAGGGCCCAGCGCTTGTGGACCAGAGGTTTACACCTGCTATGGAAGCGACACTGTC

AGGTCTCGATCTCCTGGATACATCAGTGATGACAGCGGTAGAGGCGCTAGCCCCGTCAGT

GACACTCTCTCCCCACCTCGATCTCCAGTCGCTTCTGGACCTCCTCTAATGCTGCCACAT

ATCATGGATGCGCCGCCCATTAGACAGCAATTACCTGTTGGTAGCACTCCCATTGACTAC

AACCTTCTACTGCACCGCATGTACCCAGATCTCCAGGTGCCCAAGCCAGAGTCCTCTCAT

CCTAGTGATACTCCGCGCATGGCAGTCTTCACCACGGAGACTGGCGAACGACTCACTTGT

CCCTATGATCTCCTTTTATGTCTTCAAAGAAAAAAGGAACATAACTTCATGGATGAACAC

AAACTTGTCATGGAGCAGGAAGCCATCCGTCGGCGCCAGCAACAACAACAGTTGGAAGAA

CACCTATTGCGTGAAGAGACGCGTCGTAAGCGTGAGTGTACATTCATCAACACAGTACAG

CGAGTACTGGAAGCACTCATCGGTAATGAACGGCTCGAGCAATTAGGTCATCCACAAACA

TCTGTCGACGAAGTTCTCATGCGGACGCTAAAACTTATGGGCTCACAGCCTTGCAAGGAG

CCTTCACTCTCTGCAATGGACCGGGTCAAGGTCAACCTCAGGCTGCTCCTAGAGTGCAGC

GTACCTGATCAGGACATGTGGACCAAGTTTGGCTGGAGAGGAAAGCCGATTGACGACATT

GTGTCAGAGTTCCTAAATTTCTGCTAG

>MN110023 Procambarus clarkii homeobox protein engrailed-1-like mRNA, partial CDS

TGCTCTACCGGTCTCACCGCTGTAATTCACCGCTCTCTCAAGTTCTCAATCGACTATATC

CTCAAGCCGGATTTCGGCCGCCGGCTGGGCGACGCCGTAGAGACTAGCGATCAACCCGTC

GATCTGTCGCGAGTTACCAACAGAGGGGACCCGAAGAAGGTCTTCGGAGACCCCGCCAGG

AGCCTCGGAGAGGTCGGCCAGGCCCCTAACAGCGTGCTACTGAAGGATCGAGAGGGCGGA

GGCGGCACACTATGGCCCGCTTGGGTTTACTGCACCCGTTACTCCGACCGGCCCTCTGCA

GGGCCGCGGACGAGACGGATTAAGAAGAGAGATAAGAAGGACGAAAAGAGACCAAGAACC

GCCTTTACTTCCGAACAGCTGGCCAGACTTAAGAAGGAATTCCAGGAAAACAGATACCTG

ACGGAGAAGCGACGGCAGGACTTGGCCAGAGACCTCGGACTTAATGAAAGTCAAATCAAA

ATTTGGTTCCAGAACAAACGGGCTAAGATAAAAAAGCAGGCCAAG

>MN110012 Procambarus clarkii neurogenic locus Notch protein-like mRNA, partial CDS

CAGTGCCCTTCAGGTTACTACGATGCCCGATGTCTTTCAAATGTGAATGAATGTGCCAGT

GATCCCTGTCTAAATGGTGGGTCTTGTTATGATGATGTCAACAGATTTAACTGCAAATGC

CGACCTGGGTATACTGGCCACCGTTGTGAGCATGAAATTGACGAATGCCAATCAAATCCT

TGTCAACATGGCGGTACTTGTCGTGATGCTCTTAATGCTTATACTTGTATATGTCCAGCT

GGATACTCTGGACGGAACTGTGAATCGAACATTGATGACTGCCTAAGTCGGCCTTGCAGG

AATGGTGGCACTTGTATTGATCTAGTAAATTCATACAAGTGTGTGTGTGAGCTTCCTCAC

ACCGGGCAAAACTGTGAGGTGCGAATGGATCCATGCTCACCCAACAAGTGTCGGCATGAT

GCTCGCTGTACACCTACAGCTAACTTCCTGGATTTTACCTGTGAGTGTGAACTTGGCTAC

ACGGGCCGTCTTTGTGATGAGGACATAAATGAGTGCAATGTATCACCTTCACCCTGCAAA

AATGGTGCTACTTGCAAAAATGGATATGAAGGCCGGCAGTGCACTATTAACACAAATGAC

TGTGCAATACAGCCCTGCCTTAATGGTGGTACATGCCTTGATGAAATTGGAGAATATAGA

TGTTTGTGTGTTGATGGTTTTGGAGGAATTAATTGTGAAAGTGATCTTAATGAATGCGCA

TCAAACCCATGCCAGAATGGAGCCACTTGCAATGACTATGTAAATTCTTACACCTGTAGT

TGTCCTTTAGGGTTTTCGGGAACTAATTGTGAAATTAATGATGAAGACTGCACTGAAACT

TCATGTATGAACAATGGCAGCTGCATTGATGGTATAAACAATTATACATGCGAGTGTCTC

TCGGGGTTTACTGGTTCTCATTGTCAACATCGAATTAATGAATGCCACTCAAATCCTTGC

AAAAATGGTGGTACTTGCAGTGATCATGTGGGATTCTTTACGTGTCACTGTAATTATGGC

TACACAGGACAACAGTGTGAACGTCTAGTAGATTGGTGTTCTGCCTCACCCTGCTTTAAT

AATGGAGTATGCAATCAAACCGAGAACCGCTACAAGTGTGAATGTCTATCTGGTTGGACT

GGTTTATTATGTGATGTCGAGATGGTGTCTTGTGCCACAGCTGCAGCTAGCAAACGTGTT

ATTCCCTCAAAACTTTGTCTTCATGATGGCAAATGTCAAGACATTGGAAATACCCATCAA

TGTAAGTGTGCTGTTGGCTATACAGGCTCTTATTGTCAGCACGAAATTAAAGAATGCGAC

TCCCAACCATGCATGAATGGTGCAACATGCAATGACCATATAGGTACTTACTCTTGCTCA

TGCCGCCCAGGGTTTCAAGGTCCAAATTGTGAATATAATGTTGATGATTGTAAACCAAAC

AACCCTTGTCAAAATGGAGGTGTGTGTCACGATCAAGTGAATGGATTCCAGTGCTCCTGT

CCTCATGGTACTCTCGGTAAACTGTGTGAGATCAATACCTATGACTGTTATGAAGGTGCA

TGTCATAATGGTGGAAAGTGTATAGATAAGGTTGGTGGATTTGAATGTCACTGCAAACCC

GGTTATGTAGGTGCAAGATGTGAAGGGGATGTAAATGAATGTTTGTCTTTTCCATGTGTG

AGAGAAGGCACTGCAGATTGTGTTCAGCTTGTAAATGACTATCGTTGCAACTGTCGTCCA

GGATTTATGGGACGTCATTGTGAAAGCAAGAGAGACTTTTGTGCTGAATCTCCATGCCAA

AATGGACGCACAGGAGAAACCTCTCTTCACCTTGCAGCAAGGTATGCAAGAGCAGATGCT

GCTAAAAGGCTATTGGATGCAAAGGCTGATGCAAATGCTCAGGATGCAACAGGCCGTACA

CCTTTACATGCTGCTGTTGCTGCTGATGCTCAAGGAGTTTTCCAAATACTGCTGCGAAAC

CGATCAACAGACCTCAATGCCAAAACAAATGACGGGACAACTCCTCTAATTCTTGCTGCA

CGTCTGGCCATTGAAGGCATGGTCGAGGACCTCATAAATGCTGATGCTGACATAAATGCT

GCTGATGATTCTGGCAAAACAGCTCTGCACTGGGCTGCTTCTGTTAATAATGTAGAAGCT

GTCCAGATACTTCTTGCTCATGGTGCCAACCGTGATGCCCAGAACAGCAGAGATGAAACA

TCATTGTTCCTTGCTGCTAGAGAAGGAAGCTATGAAGCTTGTAAAGTGCTGTTGGACCAC

TTTGCAAACAGAGATGTAACAGATAACATGGAGAGGCTTCCTGAAGATGTTGCTAATGAG

CGATTGCATCACGATATTGTCAGACTACTTCGTGAACATCACCCACGCTCTCCACAAATG

GCACAAGTGGTTACTACAACTCACCTTGTGCCACATCAGCATAACCAACAATCTCAAAAT

TCATTAATGTCACAACAGATGACTCCACAACCAGCTCAGCGGACTAATACTCAGCCAAAA

GCTAAAGCTCGACGGCCAAAGTCTTCAGTTATGTCCCTTCCAGGGGGATCCATAAATACT

CCAGTAAGCCCTGATGGTACGACATCGATTAAGCGTAGTTCCAGTGTTAAAAAGAAACGT

GAACCTAATGGGGTGCCCAGTGTGGAATCCCAAACTCAGCCCTCACCTCTCAACTCTTTA

GGTTCACCACATGGAGTATTTGATGCTGCTTCAAGTCCTTTTGAAACAGGCTTATTTACT

GGTGGCATGAGTGGACTGAACCCACATTTTGCAGAGGTGGGAGTAACTCAGCCTCCACCA

TATGAAGAGTGTGTAAAGGGTGCAGTGTCATTGAGTAATCTTCCACACATTGACCATGAT

AATAATCCCTTCAGCTATTCAAATATGATGGGAACTCATCAACACCAGCACCAACAAGGT

CATGTGATTCACCAACGACAGCAGTCGATGCCAGCTTCTTTCTCTCCTCAGAGTCAGACT

ATGTCTCCACCACACCACATGACTCCACCTCACTCATCTCCACAACATGTGCAGTCACCA

CATACATCAAGCCTTGTAACCTCTCCAGGCAAAATGCGGCCTGCTTTACCCGCTTCTCCC

ACACACATGGCTGCATTAAGGCATGCAACAGCTACTTCGGGATTTGAATTCCCA

>MN110017 Procambarus clarkii protein hedgehog-like mRNA, partial CDS

GTCACTCTCGCTGATGGTAAACAGAAAACTATCGCTGATCTTCGATCTGGTGATCAACTC

ATCGCTTTCAATCACAACACAAAACAACTCGTCACCACTGCACTCATCACCATGATGGAC

TTTCAACCACACAACTTCGCTCTTTTCAAACATATCACCACGTCAACAGGTAGACAACTC

TCTCTTACTTCGTCTCATCTTCTTACAACGCCTAACAACGGATATCTTATGGCCAAGAAT

ATCGAAAGTGGCATGAACATCTATGTTGTCAATGAAGAAGGTGCATTGATCGAGGATCGT

GTGTCGAATGTAACTGATGTTGTAAAACAAGGATACATGGCACCTTTGACACAAGAAGGG

ACGTTGATCGTGAATCATGTCGCTGCATCTTGTTATGCAATAATCGATAGTCACGATGTG

GCTCATGCAGTCCTGGCACCGATGCGTTGGTGGTACAATCTCTTTGGTGCAGGATCGAAA

AGAGATGAAACAATGTCTGTTGGTGTCCATTGGTTTCCAAAGATGCTGTTCGATGTCACA

GCATCTGTTTTACCATCGGTGATTCAAAAATAG

>MT942642 Procambarus clarkii homeobox protein Dlx2b-like mRNA, CDS

ATGCCGGATCAAGATCTGGCTTCGAAGTACATGGACCTACCCCAGCAGGGCTTGGCTAGC

ATGGCCCATACCCCGCCCTATGCCCAGCCCTTAGGGTACCAACAGCCTCCCACACCTGGG

TATAACCCACCGGGCTACGGGTTTCCACCCATGTACCCACAGACGTCATACCCAGGCTAC

CCAATGGGTTCATACCTCACTTCGCAATGCCCTTCGCCCTCTGTAGACGAAAAACCTGAG

GACGAAGGTTCTGTGAGAGTAGGCGGAAAAGGCAAGAAAATGAGGAAGCCGAGGACCATC

TATTCATCATTGCAACTACAACAACTCAACAAAATATTCCAGAGAACGCAATACCTCTCC

CTGCCCGAACGTGCTGAACTGGCCGCCAAACTCGGTCTCACACAAACTCAGGTGAAGATC

TGGTTTCAAAACAAGAGGTCCAAGTGCAAGAAGATTATGAAGACAGTTAACTCAGGCGGG

CCGTGTGTGCCTCAGGGTGCCCCGGTAGGGCCCGGGTCTCCTCTCACCTCCTCTCCCATG

TCAGTCACCTCTCCTCTACTCACCTATACAACCCCTACCCCTATCAGTCCCCCACCCCAG

GCGCTGTCCCCGGCTCAGCCCTATACAACTCTAGCTCCTATCCATCACCCTCGATCCCTA

CCATCGCCTGGGGCCTGTCCACCGGGGCATCCTTACTGGCAGCAGCAGCATGACTCGCCC

CAGCTCCCACCATCCCATTCTATCCCACAGGCATCTCAGCCTGGGTCACCTATCCCACAT

GTCCAACAGCAACTCCATCACCATCACTCAGTTTCTTCAGACTCCCTTTCACAGTCTTCT

CTCCCCATCTATCCTCCCCACACGGCCTCTCCTCACTCCCACCCACAGCACCCCCAA

>MT942643 Procambarus clarkii homeobox protein DLX-6-like mRNA, CDS

ATGTTGGACCAGGAGCTACTAGCCAAGGGCGGACTGTCTGACAGCCAGCAACAGCCGCCA

CCGCATCTAACTAACCCATATTCCCAGTTTCAACAGTATCAGCAAAGTATGGCTGGTTAC

AACAATATGGGCTATGGTTTCCCGGCGATGTATGCCCAAAATGGCTATGGGTACCCGCTC

CCTGGCTATCCACATGCCCCAAGTCCGCCCTCAGATGTGACGGAGAAGACAGAGGGAGGA

GAGGTTCGTGTGACAGCCAAGGGCAAGAAGGTTAGGAAGCCTCGCACCATCTACTCCTCC

TTGCAGCTACAGCAACTCAACAAGATGTTCCAGAGGACTCAGTATCTGGCCCTGCCGGAG

CGCGCCGAATTGGCCGCTAAACTTGGTCTTACGCAAACACAGGTTAAGATCTGGTTTCAG

AACCGCAGGTCCAAGTATAAGAAGCTTTACAAGGCGGCTCAGAACGGCCAGCTGCCGGGA

GTCGACGCTGCTGAAATTGCTGCAGATCTGGGAGCCAACTTGTCACAGATGGTGTCGAAC

GGGCCTGAATCACCAAGCTCTCCAGCCACCACAGACCATGGTCACGATCAGCCACCGTCG

TCTGTCGGACCCGACGGTGGCCCACTTTCTCCTCCGCCCGACGACCGCCCACAGTCTCAA

ACTCCAACGGTATCGAGCCACGGGGAGATGACGTCTCCTATGATGTCGGCTCCACGGGAC

ATGATGGTGTCTCCACCAGCTCATCCGTCCAAGGACATGATGGCGATGAATCAGGCAATG

GCCGCTTGCGAACAGCAGCGACGGGATCAAGCTATGATGCCGCCCCATCACCAGTGGGAT

CCGGCCCACTATATGTCATACTGGAACCACTATGGGGACATGGCGGCCCATCAGATGACC

CATCAGATCATGACCTAA

>MT942647 Procambarus clarkii zinc finger E-box-binding homeobox protein zag-1-like mRNA, partial CDS

AAGAAAATGTTAGAAAGTGTCAACACAAGTGTCACAAAACATCAGTTTGAAGAAACAGTA

TCATCAGCTTCTCAGGGCTTTGCACTAGACTCTGCAGCCATTGCTGATGACCTTTCTTGT

AGGCTGTGTGGCAGTACCTTCAGAAATCGTAGTGAAGCTTTTCTACATGCCTGCCGCCAG

TGTCCGCGTCTTCCTGAAGTTACCTCTGCCAAGGGACATCTAATTGAGGGTCTGGCAGCA

AGGTTACACGAGCTAGCAGAAAATCAGAAACCTCAACGCCATCAACAACGGCAGCAACCA

TCTCAACATATTTCTCAGTCCCAATATATGCATAGTCGGGTCATTTTGAGTAGGGATGAG

GAGAGAGATGCAGATTCTGGCCATATTGTAGATGATGAAGAGACCACCAGTGATGGAAAG

AAGGTTCGTGTACGTTCTCACATCCGTGAGGAGCAGCTTATTGTCCTGCGAGCCCACTAT

GCTATTAACCCCCAACCTAAGAAAGAAGAATTGCTAACCATTGCTGAAAAGATTGGTTTT

CCAGTTCGAGCGGTGCAGGTATGGTTCCAGAATGCTCGAGCTCGTGACCGTCGTGAGGGT

CGGTCAATTACC

>MT942648 Procambarus clarkii zinc finger homeobox protein 3-like mRNA, partial CDS

GAGGAGGCGCGCGAGATTGTGGCGATCAGCAACGCCCGGGAGGAAGAGAGAGTTGCAGAA

CGCATCCCAAAGGAGCAGGAAGAGCCAGCCCCTGAACGCGCCGCCGCCATGTCTCCGCTC

AACAAAGTTCCTAGTCTTAACCAAGACAATAGCCGAGTACAAGAAAAGAGCGGGGACGGC

TTAGGTGGATCAGGTGGCCTGAATAGTCAGCAACAACTCCAGCAACATCAGTCACGGGAA

CAACAACATCAACAACAACTGTTGGAACAACAACAACAACAACGGGAGCAGCAGTCACAA

CCATTGCTTCCTCCACATCACCAACAACAAGTAAGCAGTGGTCCCCAACTGGTGTCGTCA

GCGTCGTCGTCCCCTCTGGGGCTGCCGGCAACCTCATCGTTCACCTCCCTGGCCTCACCT

CAGTCATCAATGTCACTCACCTCACTCATTACCTCCCAGCTTGAGACCAACCCCATGCTG

GCTCACAAGCTGCCTCACACACCACCCACAGTTCCTAGTTCTGGCCTTATTCCTCCAAGC

TCAGGTGTGAGTCCCACGCCGCCCCATTTGTCCTTCCCATCAACTCCACTCTCTGCCCCT

CACACCCCTACCTCTTCCTGCTCCAGCACGGGCAAGCGAGCCAACCGCACCCGCTTCACT

GACTACCAGATTAAGGTGCTACAAGAGTTCTTTGAGAACAACGCCTACCCTAAGGACGAT

GATCTGGAGTATTTAACCAAGCTGCTCAACCTATCACCACGTGTCATTGTGGTGTGGTTT

CAGAACGCCCGACAAAAGGCACGCAAAGTGTACGAGAACCAGCCACCCATTGACCCAAAC

GACGAAGGCGCGGGCAGGTTCACGCGGACGCCGGGCCTCAATTACCAGTGTAAGAAGTGC

TTGCTTGTCTTCCAGCGGTACTATGAGCTCATCCGACACCAGAAGACACATTGCTTTAAG

GAAGAAGATGCCAAGCGCTCAGCTCAAGCGCAAGCAGCTGCAGCCCAAGCAGCAGCTCTG

TACAATGACGAGAACTCCAACCACTCCAGTGTCACTGATTCGTCACAGCAGTCTGGAGGA

CTAGACTGCAAGTCCAGTGAGGGTACTTTTCAGTGTGATAAGTGCAGTCTGATATTTAAT

AGGTTTGAACAGTGGCGGGAACACCAAATTGTTCACCTTATGAACCCTGCACTCTTTTTA

AACAAGGGTGCTGATTCTCCCTTTACCAGCATACAGCAGCAGCAACAGCAGTCTCAACCT

CCACAGCAGCCACAACAGGCGCCCACATCCATACCTCAGCCCCAGCCCAGCCCCCTCTTG

CCCCCTGCCTCGCCTCTGAAGCGTAAAGCTGACGAGAGTGAGGATGAGAGAGAGAGCATG

ATCGGGGCCAGTGAGGCCCAGAGGGACAAACGGCTCCGCACCACCATCCTTCCGGAGCAG

CTGGACTACCTATACCAGAAGTACCAGATGGAGGCCAACCCGTCGCGGAAGATGCTCGAG

ACCATTGCTCAGGAAGTGGGTCTCAAGAAGAGGGTCGTCCAAGTCTGGTTCCAGAATACA

CGAGCGCGAGAACGGAAAGGTCAGTTTAGAGCTCATGCACAAGTGATTAACAAAAAATGT

CCCTTCTGTCCTGCTATCTTTAAAGTCAAGTCTGCTCTTGAGTCGCACCTTTCCACTAAG

CACGCGGAGCATTACTCTAAGGGAGATGTGGACATTGATGCACTACCTGATGTAGAAGAC

TCTGGTGTTGGGAACTTCGGCCTGTCTTCAACTCCTTCTGCAACTCAAGCTTCTCAAGTG

ATGCCCTCTTCTCTCTTCTCCTCAGAAGTTCCGGAAGACTCGATAGCCATGTACCACGAG

GAGGCAATCCGGCGGTACCTGAACGATGTCAACTTGTCATCAGATGGTACCCGACGAGAA

GGTGAGAGTCCCCTTGATCTCAGTAAACCCTTAGACATTGTGCGGCCGTTGGGGTTTGAC

TCATCTATACTGGACAACACCAACGAGCAGCTGGACGACCACTCTGACGAGGAGAGCTAC

CACCTGGACATGTGCGAGCACGACGAGGGCGACACCGGCTTGAACTCTCACGAGAGCAAC

CCGACCTCGCCCGCCTCCTCCACCACCAGCTCCGCCAAGCAGTCCGGGTTCATGCATGGG

GCTCCCAACAAACGGTTCCGCACCCAAATGTCGGCAACGCAGGTCAAAATCATGAAAAGT

GTGTTTCAGGATTATAAGACGCCTACAATGGCGGAGTGTGAGTTGCTGGGGAGGGAGATA

GGGCTAGCCAAGCGTGTGGTGCAG

______________________________________________________________________________________________________

SET 3. Phtototransduction, Rhabdomeric

>MN110024 Procambarus clarkii serine/threonine protein phosphatase 1 mRNA, partial CDS

ATGGCTGAGACCGATAAGCTTAATATAGACACCATAATTGCCAGATTATTAGAAGTGCGA

GGATCTCGGCCTGGAAAGAATGTCCAGTTGACAGAAAATGAAATTCGTGGTCTCTGCTTA

AAATCCAGAGAAATCTTTCTCTCTCAACCTATCCTATTGGAATTGGAAGCGCCTCTTAAA

ATTTGTGGTGACATTCACGGACAGTATTATGATTTGTTGCGACTATTTGAATATGGAGGG

TTCCCACCAGAGTCAAACTACCTTTTCTTGGGAGACTACGTGGACCGTGGTAAACAGTCA

CTTGAAACGATATGTCTGCTACTGGCCTACAAAATAAAGTATCCTGAGAACTTCTTCCTT

CTCAGAGGCAACCATGAGTGTGCTTCAATCAACAGAATCTATGGCTTCTATGATGAGTGC

AAACGACGGTACAACATCAAATTGTGGAAAACCTTCACAGACTGTTTCAATTGCTTACCA

GTTGCAGCGATTGTTGATGAAAAGATCTTTTGTTGTCATGGTGGGCTGAGCCCCGACTTA

CAAAGTATGGAACAGATTCGTCGCATTATGAGGCCCACTGACGTCCCCGACCAAGGGTTA

CTCTGTGATCTCTTGTGGTCTGACCCAGACAAGGACACTATGGGATGGGGGGAAAATGAT

CGAGGGGTCTCTTTCACATTTGGCGCAGAAGTTGTTGCCAAATTCCTTCACAAACACGAC

TTTGACCTCATTTGTCGTGCTCATCAGGTTGTTGAAGATGGCTACGAGTTCTTCGCAAAG

AGACAGTTAGTGACACTCTTCTCGGCACCAAACTATTGTGGAGAGTTTGATAATGCTGGA

GCAATGATGTCTGTAGATGAAACACTCATGTGTTCTTTCCAGATACTTAAGCCTGCTGAC

AAAAAGAAGTTTCCTTATGGAGGTTTGAATACCGGCCGACCTGTTACGCCACCAAGAGGA

GCTGCCAATCAGAAAAACAAAAAGAAA

>MN110029 Procambarus clarkii serine/threonine-protein phosphatase 2A catalytic subunit beta mRNA, partial CDS

ATGGATGACAAAACACAAATGAAGGAGTTAGACCAATGGATAGACCAGCTGATGGAGTGT

AAACAGTTGGCGGAGAATCAAGTGAAAACACTATGTGAGAAGGCTAAAGAAGTTCTGGCA

AAGGAGAGCAACGTTCAGGAAGTAAAGTCTCCTGTCACAGTTTGTGGAGATGTTCACGGA

CAGTTCCATGATCTTATGGAACTATTCAAGATTGGAGGACGGTCTCCAGACACAAATTAC

CTCTTCATGGGAGATTATGTTGATAGAGGCTATTATTCTGTAGAGACTGTTACATTGTTA

GTTTGTTTAAAGGTGAGGTTTCGTGAACGGATTACAATCCTCCGTGGCAACCACGAATCC

CGACAGATCACGCAAGTTTATGGTTTTTATGATGAATGCTTGCGAAAATACGGAAATGCA

AATGTTTGGAAGTATTTTACGGATTTATTTGACTATCTTCCCTTAACGGCGTTAGTAGAT

AGTCAGATATTCTGCCTGCATGGTGGACTTTCACCATCTATAGACACACTTGACCACATA

AGAGCACTTGACCGGTTGCAGGAAGTACCTCATGAGGGTCCAATGTGCGATCTTCTATGG

TCTGATCCAGATGATCGTGGTGGTTGGGGTATTTCTCCACGAGGCGCTGGTTACACTTTT

GGCCAAGATATATCGGAGACCTTTAATCACTCCAATGGTTTGACTTTGGTCTCGCGTGCA

CACCAACTGGTTATGGAGGGTTACAACTGGTGCCACGATAGAAACGTAGTCACAATCTTC

TCCGCACCTAACTACTGTTATCGTTGTGGCAATCAAGCAGCTATTATGGAGCTTGACGAC

TCCTTGAAATATTCATTCCTGCAGTTTGACCCAGCACCTAGGAGAGGGGAGCCTCATGTT

ACACGCCGAACACCAGACTATTTCTTG

>MF279133 Procambarus clarkii strain Pc02122016 guanine nucleotide-binding protein G(q) subunit alpha (Gnaq) mRNA, partial cds

ATGGCGTGCTGCTTAAGCGAAGAAGCCAAGGAACAGAAGAGGATAAACCAAGAGATAGAGCGACAATTAC

GCAAGGATAAGAGAGATGCTCGAAGAGAACTTAAACTACTGTTATTGGGCACTGGAGAATCTGGCAAATC

AACATTTATCAAACAAATGCGAATTATCCATGGTGCTGGTTACAGCGATGAAGATAAGAGAGGGTTCATC

AAGCTGGTCTTCCAGAATATTTTCATGGCCATGCAGTCTATGATCAGGGCTATGGATCTCCTACAGATAT

CGTATGGAGATTCAGCCAACATTGAACATGCAGATTTGGTAAGATCAGTGGACTATGAGTCGGTAACTAC

ATTTGAGGAACCATATGTGACTGCTATGAAATCATTATGGCAAGATACAGGCATCCAACATTGCTATGAC

CGACGCAGAGAGTACCAGCTTACAGATTCCGCAAAATACTATTTAACAGATTTAGACCGCATAGCTGCCA

AGGACTATGTTTCCACACTACAAGATATTCTAAGAGTGAGAGCACCCACAACAGGCATTATAGAGTATCC

CTTTGACCTAGAAGAAATCAGATTTAGAATGGTAGACGTGGGTGGTCAGCGATCTGAGCGGCGGAAGTGG

ATCCATTGCTTTGAGAACGTCACTTCTATCATCTTCCTTGTTGCTCTCTCGGAATATGATCAGATTCTCT

TTGAATCTGACAATGAGAACCGAATGGAGGAATCTAAGGCCCTTTTTAAGACTATCATCACCTACCCCTG

GTTCCAGCACTCTTCTGTTATCCTTTTCCTTAACAAGAAGGATCTGTTAGAAGAGAAGATCATGTACTCA

CATCTTGTTGACTACTTTCCAGAATATGATGGCCCACGGAAGGATGCCATTGCAGCCCGAGAGTTCATCC

TACGGATGTTTGTAGAATTAAATCCTGACCCTGAGAAGATTATCTATTCTCATTTCACATGCGCGACAGA

CACTGAGAACATAAGGTTCGTCTTCGCTGCTGTCAAAGATACGATCCTGCAGCTAAATCTAAAGGAATAC

AACTTGGTG

>MN110031 Procambarus clarkii Guanine nucleotide-binding protein G(s) subunit alpha mRNA, CDS

ATGGGTTGTTTTGGTAGCGCTGGGGCGAAAGGTGACGCCGAGGAAAACAAAAGGCGGAAA

GAAGCAAACAAGAAGATAAACAAGCAAATCCAGCAAGACAAGCAGGTGTACCGAGCGACG

CACAGATTGTTATTACTAGGAGCCGGAGAATCAGGGAAAAGCACCATTGTGAAGCAGATG

AGAATTCTACATGTCGATGGATTCAGTGAAGAAGAAAAAAGAGAAAAGATCCATGCCATC

AGGTGCAATATCCGCGATGCCATCTTGACCATCACCGGCAATATGTCTACCTTAACGCCC

CCAATAGCGCTCGAAAACCCAGCCCACCAGTTCCGCGTCGACTACATCCAGGACGTGGCC

TCGCAGAAAGACTTTGACTACCCGGAGGAATTCTACGAACACACCGAGATGTTGTGGAAG

GACAAGGGAGTACAGGCCTGCTACGAGCGTGCTAACGAGTACCAGCTCATAGACTGTGCC

AAGTATTTCCTTGACCGGGTCCACATTGTCCGACAGCCAGACTACACTCCCACTGAGCAA

GATATCCTACGCTGCCGAGTCCTTACACTAGGAATTTTTGAGACCAGATTTCAAGTAGAT

AAAGTTAATTTCCATATGTTTGATGTGGGTGGACAGCGAGATGAAAGGAGGAAATGGATC

CAATGCTTCAATGATGTCACCGCCATTATATTTGTCACCGCTTGCTCGTCTTACAACATG

GTTCTACGAGAAGATCCCAGTCAAAACAGGCTACGGGAATCTTTAGATCTCTTCAAAAGT

ATATGGAATAACAGATGGCTACGCACAATAAGTGTTATCTTGTTTTTAAATAAGCAAGAC

CTGCTGGCAGAAAAGATCCGGGCAGGCAAGAGTAAGCTTGAAGAATATTTCCCCGATTTT

GCCCGGTACCAGACCCCACTAGATGCCACTGTTGAACCTGAAGAGGTACCGGAAGTGGTA

CGCGCAAAGTACTTCATCAGGGACGAATTTCTAAGGATAAGCACAGCCAGTGGTGATGGG

AAGCACTACTGCTATCCTCACTTCACATGCGCCGTGGACACTGAAAACATCCGCAGAGTG

TTCAATGACTGCAGGGACATAATACAAAGGATGCATCTCAGACAATATGAACTTTTG

>MN110025 Procambarus clarkii Guanine nucleotide-binding protein G(i) subunit alpha mRNA, partial CDS

ATGGGGTGTGCGATGAGTACAGCTGCTGACAAAGAAGCAGCAGAAAGAAGCAAAAAAATT

GACAGGGACCTGAGACTTGCTGGAGAACGTGCTGCCAGAGAAGTGAAGCTTCTGCTCTTG

GGTGCTGGTGAATCTGGCAAAAGTACAATTGTAAAACAGATGAAGATTATACACGAGACA

GGATATTCCCGGGACGAATGTGAACAGTACCGACCGGTCGTGTATTCCAATACAATCCAG

TCACTCATGGCAATAATCAGAGCCATGGGGCAACTAAAGATTGACTTTAAAGATTCTAGT

CGAGCGGATGATGCCCGGCACTTTTTCACATTAGCAAGTGCAGCAGATGAAGGTGAATTG

ACTCCTGAATTAGCAAACATAATGAAGCGATTGTGGAATGAGAGCGGTGTCCAGCACTGT

TTTAGCCGGTCGAGGGAGTATCAGCTAAATGATTCCGCTGCCTATTACCTAAATGCCCTG

GACCGGATTGCTCGGCCTGGCTATGTTCCCACACAGCAAGATGTCCTCCGCACCAGAGTT

AAAACAACAGGCATTGTGGAGACAAAATTTTCTTTCAAGAACCTAAACTTCAAGCTGTTT

GATGTAGGCGGACAGAGATCCGAGAGAAAAAAGTGGATCCACTGCTTTGAGGGAGTGACG

GCCATCATCTTTGTTGTTGCTTTATCAGGGTATGACTTGGTACTAGCAGAGGATGAAGAG

ATGAACAGGATGATTGAAAGTATGAAGCTCTTTGACTCCATTTGCAACAACAAATGGTTT

GTGGAGACGTCAATAATTCTCTTCCTAAACAAGAAAGATTTGTTTCAAGAAAAGATTACA

AAATCGCCATTAACGATCTGTTTCCCAGAGTACCAAGGCAACAACACGAACGAAGATTCC

GCCAACTACATCCGTATGAAGTTTGAGAACCTCAATAAGCGGAAGGATCAGAAAGAGATC

TACACGCACTTCACCTGTGCTACCGACACTAGTAACATTCAGTTTGTGTTTGATGCTGTA

ACAGATGTAATCATCAAGAATAACCTCAAGGATTGTGGTCTTTTT

>MN110018 Procambarus clarkii guanine nucleotide-binding protein G(o) subunit alpha mRNA, partial CDS

ATGGGCTGTGCCATGTCTGCGGAGGAGCGCGCCGCGCAGGCCCGCAGCAAACAGATCGAG

AAGAACTTGAAGGAGGACGGCATCCAGGCAGCGAAAGACATCAAACTGCTGCTGCTGGGC

GCGGGAGAATCCGGCAAGAGCACCATCGTAAAACAGATGAAGATCATCCACGAGTCCGGG

TTCACGAGCGAGGACTTCAAGCAGTACCGGCCGGTGGTGTACTCCAACACCATACAATCC

CTGGTGGCCATCCTGCGGGCCATGCCCAACCTGGGCATCTCCTTCGGCAACAATGAGAGG

GAGCCGGATGCCAAGATGGTCTTTGACGTAATCTCTCGGATGGAAGACACGGAGCCCTTC

TCTGAGGAGCTCCTGTCGGCGATGAAGCGGCTGTGGGCCGACACGGGCGTCCAGGAGTGC

TTCGGCCGCTCAAACGAGTACCAGTTGAACGACTCTGCTAAGTACTTCCTTGACGACCTT

GACCGCCTGGGGGCCAAGGAATACCAACCCACCGAACAAGACATTCTCCGGACCCGTGTC

AAGACCACGGGCATCGTCGAGGTTCACTTCTCCTTCAAAAATCTCAACTTCAAGTTGTTT

GATGTAGGTGGGCAGCGATCAGAACGCAAAAAGTGGATACATTGTTTTGAAGATGTAACT

GCTATCATCTTTTGCGTTGCCATGTCTGAATATGACCAAGTTCTTCATGAAGATGAAACC

ACGAATCGCATGCAAGAGTCATTAAAACTATTTGATTCCATCTGCAATAATAAGTGGTTC

ACCGAA

>MN110034 Procambarus clarkii guanine nucleotide-binding protein subunit beta-5-like mRNA, partial CDS

CCTACTGGAACCATGGTTGCTTGCGGAGGCCTGGACAACAAGGTCACGGTCTACCCTCTC

AGTTTTGACGAAGATGTCACTCAAAAGAAGAAGGCTGTCGGCACCCACACGTCTTACATG

TCCTGCTGCACCTTCCCTTATTCCGACCAGCAGATCCTGACGGGCTCTGGGGACTCTACG

TGTGCTCTGTGGGACGTGGAATCCGGGATGATGTTGCAGAGCTTCCACGGCCACCAGGGG

GATGTAATGGCCCTAGACTTGGCCCCTTCAGAGACAGGAAACACATTCGTCTCCGGTGGA

TGCGACAAGATGGCACTCATCTGGGACATGAGAACTGGTCAGTGTGTACAGACGTTCGAA

GGTCACGAATCTGACATCAACACCGTCAAGTTCTACCCGTCGGGAGACGCCATTGCCACG

GGTTCAGATGACGCCACGTGTCGGTTGTTTGACCTGCGGGCGGACCGAGAGATCGCCGTC

TACACGAAGGAAAGCATAATTTTCGGTGTCAATTCCGTCGACTTCTCCGTCAGAGGACGA

CTGCTGTTTGCTGGCTACAACGACTAC

>KY974308 Procambarus clarkii Guanine nucleotide-binding protein G(I)/G(S)/G(T) subunit beta-1 (GNB1) mRNA, partial cds

ATGAATGATTTGGATAGTTTACGACAAGAAGCAGAAAGACTAAAGAACACAATACGAGATGCACGCAAAA

ATGCACTTGACACGACACTGGTCCAGGCCACAGCTGGCATGGACCCTATTGGCCGAATTCAGATGCGAAC

CCGGAGAACGCTTAGGGGACACTTAGCCAAAATATACGCCATGCACTGGGGATCCGATTCTAGGAATTTG

GTGTCGGCATCTCAAGATGGCAAGCTTATAGTGTGGGACAGTTACACAACAAACAAGGTGCATGCCATCC

CCCTTCGGTCCAGCTGGGTCATGACCTGTGCCTATGCTCCCTCAGGCAGTTACGTTGCCTGTGGTGGCCT

TGATAATATCTGTTCCATATACAGCCTAAAGACAAGAGAAGGCAATGTGAGAGTGAGTAGGGAGTTGCCT

GGTCACACTGGTTACCTAAGTTGCTGTCGGTTCGTAGATGACAACCAAATAGTCACAAGCTCTGGAGATA

TGACTTGTGCTCTTTGGGATATTGAAACTGGTCAGCAGTGCACACAATTTACAGGACATACTGGGGATGT

GATGTCCCTATCTTTGTCGCCAAACATGAGGACCTTCACATCGGGTGCCTGTGATGCCTCGGCAAAGTTG

TGGGATATCCGTGATGGCATGTGCCGCCAGACTTTCCCAGGACACGAATCAGATATCAACGCAGTAACAT

TCTTCCCTAATGGGCATGCATTTGCTACGGGCTCAGACGATGCCACCTGCCGCTTATTCGACATTCGTGC

AGATCAAGAACTGGCCATGTACTCTCATGATAACATAATCTGTGGCATCACATCAGTGGCATTCAGCAAG

TCTGGCAGACTTCTGCTGGCTGGTTACGATGATTTTAACTGCAATGTTTGGGACTCCATGAGGACAGAAA

GAGCTGGAGTTTTGGCGGGCCATGACAATCGCGTCAGTTGCCTGGGTGTTACAGAAGATGGCATGGCAGT

GGCCACTGGCTCGTGGGACAGTTTCCTCAAGATCTGGAAC

>MT601685 Procambarus clarkii Guanine nucleotide-binding protein subunit gamma-1 mRNA, partial CDS

AGCAAAGGCACTACAAATCACTTTGGCAAAGCCCGTTCCTCTGTCGCCAGCAAGATTGTG

CGTACCGCAGTGGAATACACACTCAACAACATCATGATGGATCAGATGTCGAGTCATCAG

CAGTTACGCGTTCTCGTAGAACAGCTGCAGCGAGAAGCTAATATAGATCGCATGAAAACC

TCGGAGGCAATCAACCATCTCAAGAGGTACATCTCGGAACACGAGGCAGAGGACTATCTT

CTGGTCGGCTTCACCTCTCAGAAGGCCAACCCATTCCGCGAGAAGAGCTCCTGCACCGTC

CTGTAA

>MN110015 Procambarus clarkii protein no-on-transient A-like mRNA, partial CDS

ATGGAGAACGGTTCCCCGCAGAAGAGTCGTGATGGAGGAGGCAACATGGGAGGCCGTGGA

GGTGGTAACTACCAGGGAGGCCACGGAGGCCACGGAGGAGGAGGTGGAGGCGGCGGCGGC

GGCCAGGGAGGCCAGAAGCGTGGAGGGCCGCCGCCCTATGGCCGCCACCCTCAGTATGGC

TACGACAAGATAAAGGAGAGAGTGAACACAGAACTGAGTGGTCCACAGCTTGATTTGCCT

CCCCTTGACCTAGCAGAGAAGAAGTTTAATGCTAGTAGCCGCTTGTTCGTGGGTAATCTA

CCCCGGGACTTGCCTTATGAAGAACTCAAGGAAGTCTTTTCTCAGTATGGAGAGTTGGGT

CAAGTGTATTTCAACAAAGATGGAGCATATGCCTTCATTAATTTTGATTACCGAGCTAAT

GCCGAGAAGGCTAAGCGCGAATTGCAAGGAAAGAATTGTCGCAATCGTCCCATGAAGATC

AGATACGCATCAATATCTACGGGTGTGAGAGTCAAGAATCTAACCCCTTGCGTGTCGAAT

GAGTTGCTGGAAAAAGCCTTCAATGTATTTGGTCAAATCGAGGCGTGCCGTGTGATTGTT

GACGACCGGGGAAAAACCACAGGTGATGGCATCGTGATATTTGCTGACAAGAAAGGTGCC

ACACTTGCCATCAAGAAGTGTCAGGAGGAGTGCTTTTTCCTCACCAGTGCTCTGCGTCCT

GTAATTGTCGAGCCCCTTGAGGCTCGGGATGAGGAAGAAGGCCAACCTGAGAGTTCCATG

CATAAGAATGAGCAGTACAAGCAGGAACGCAAGGAGGGCCCCAGATTTGCTGATCGAAAC

TCCTTCGAATATGAATATGGAATTCGTTGGAAGCGTCTGTATGAAATGTACAAGGAAAAG

AAATTGTTGCTAGAGTCTGACCTGCAAGCAGAGATGGAACAGCTGGAAAGACGACTGGCT

ATTGTGAAGCACGAGCATGAGACGGAGAAGTTGAGAAGAGAGTTGATGGCACGTGAACAA

GAGGCAATGCAGCTCGGGAGATCGCTGTACGGGCGCCAGGGAGGCTATGGAATGGATCGT

TATCAGGGATCACAGGATGAACGCTATGGAGAACGCTACGATGCTTCACGGGATGAACGA

TATCAGCAAGCCATGCAGGAAGAACGTTATCAGAGAATGCAAGAGGAACGGTATCAGCAA

GCCTTACAGGAGGAGCGGTATCAAGCTATGCGCAATGCTGGCTATCAACAAGGTGGTAAC

GCACAAGAGGAACAGTTCCAGCAACAGGGAACTAAGAGAGAGGCTCCAGCAGCATCGGCA

CAAGTAGCTGCCGGAGGGCCAGCGGCAGCAGCAGGACCCGGGGCAACATCTGACGAATCT

GAAATGAAGCGAGGGAGATAC

>MF279134 Procambarus clarkii strain Pc02122016 eye-specific diacylglycerol kinase isoform X3 (rdgA) mRNA, partial cds

TCTAATGCCGCTCAACCTCTGAAAATTGAGCCAAGGAAAGCGATCAACTCTGAGGTGGAGATCCAGTGTG

AGAATGGGGAGCGGCGCGTCCTGCGGTCCACCTGCGACTGGAATGAGGGAGCTATCAATGGGGACCACCT

GTGGTGCCCGACCTCCGCCTCCGGCGACTTCTGCTACGTCGGCGAGGACTTCTGCTGTAAGTCGGGGGCC

CGGATGAAGTGTTCCGCCTGTAAGATCATCGCCCACACGGGTTGCATCGCGGCGCTGGTGGAGCGGGTCA

AGTTCCCCTGCAAGCCCACTTTCCGCGACGTCGGTCCGCGCCAGTACAGGGAGCAGACGGCGACGCACCA

TCACTGGGTCCACAGACGAAGCCAGAAGGGCAAATGCAAGCAATGTGGCAAGGGATTCCAGTCCAAACTG

CTCTTCGGCTCCAAGGAGATCGTGGCAATCAGCTGTTCCTGGTGCAAGTCTGCTTACCACAACAAGGAAA

GCTGCTTCAACATCAGCAAGATTGAGGAAACCTGCTCTCTGGGCCTCCACACAAGCATTATCGTGCCGCC

ATCTTGGATCGTTAAGCTACCTCGCCGCGGCTCCTTCAAGTCGTCTCTCCGGAAGTCTCCCAAGAAGAGA

GCCTCAAACAAGCGTAAAAGCCGAGATAAGCAAAACGATAAAGAGACGCGGCCCTTCGCCATCAAGCCAA

TCCCCAACGCCAACATCAAACCGCTGATAGTGTTCATCAACCCCAAGAGTGGAGGCAACCAGGGCGCCAA

GCTTATGCAGAAGTTCCAATGGCTCCTCAACCCAAGACAGGTGTTCGACCTCACCCAAGGAGGGCCCAAA

GCAGGACTAGAGATGTTCCGGAAAGTGAGCAACCTGCGGGTGCTGGCGTGTGGCGGGGACGGCACTGTGG

GCTGGGTGCTCTCCGTGCTCGACCAGCTCAACTTCCAGCCTCCCCCGGCCGTGGCTGTCCTGCCCCTGGG

CACGGGCAACGACCTCGCCCGCTCCCTCGGCTGGGGAGGAGGGTACACAGACGAGCCCATCAGCAAGATC

CTTTGCAATATCGCTGATGGAGAGGTGGTTCATCTGGACCGGTGGCGTGTGGATGTTGTCAAAAACGAAG

AGTACGAACCTACTGAAGAAGGCAGAGACACTCTCCCCCTTAGTGTTGTCAACAATTATTTTTCCTTCGG

TGTCGATGCCCACATTGCTCTCGAATTCCACGAGGCAAGAGAGGCCAACCCACAGAAGTTCAACTCTCGG

CTTCGCAACAAGATGTTCTATGGGCAAGCTGGAGGCAAGGACCTGCTGCAGCGTAAATGGAAGGACCTCT

CCGATAACTGCACACTAGAGTGTGATGGCAAGGACATGACCCCTATACTGAAGGAAAATAAGGTCCATGC

TGTGGTTTTCCTCAATATACCCAGCTATGGCTCTGGCACCCATCCTTGGAATCGTAGTAGTGGAGTGGAA

CAGCACACAGATGATGGCATCATTGAAGTTATTGGGCTCACCACATACCAGATGCCACTGCTACAGGCCG

GGGGTCATGGGACAACAATCTGCCAGTGCAAGAGAGCCAGGATTATCACCAGAAAGACTATTCCCATGCA

GGTGGATGGTGAGGCAGCGAGGGTCAATCCTAGCATCATTGAGCTTACTCACCTCAACAAGGCTTCCATG

GTGACTAAGAAGAAAGCTAAGTCTGTCACGATGCCTCATCTGGAACAGCTTCGTTTGCAAGTCTCAAGGA

TCACCATGTATGACTACGAACAGCATCATTATGACAAGGAGAAGTTGCGAAGTGCTTCTTCTTCCATTGG

TGTCATTATGAGTGACCAGGATGCTGACCTTGAGCAGGTTCGGAAGCACATAAATCGTATGATGGAGGAC

GCGTCAAATAAGGGCAGGTCTGCTCGGCCCTTGAGTGAGAGTTGGTGCTTCCTTGATTCATGCACAGCTG

AAAGGTTCTTCCGTATTGATCGGGCCCAGGAACACCTCCACTACGTGACGGACATTTGCACCGAGGACCT

CTTCGTGCTGGACCCAGAACTCACCGACCCCACCAGCGTCAGCAGTGGCGCCATTGTGAGAGCAGATGTT

AGCGTGGACAACGACATAGATCATAGCGAGAGGATGGCCATGGTAAGCGACAGTGACTCTGGCGTGGGTG

GAACTGATGCGGAGCAGCGAAACCCTACCTCTGCCCTCACCGCCAGCCAAGACTCCTCCGACACCGGGGA

GGAGGAGGCCCTGGAGGCCCGGGTACCCAGCCACCTACTTGAGAAGAACTCTGATGGAATCATAAAGGCT

GCCAAACAAGGTGATTTGAAGATGCTAAAGGACCTCCATTCTCAAGGATACTCATTGCTGTCAATTGATG

GACATGGACAGACTGCACTTCATTTTGGTGCACGCCATGGACACAAAGACATTGTGCGCTACCTAATTGC

ATCTGCCCCACCATCCATCCTCGACATGGTGGACACAGAGAGAGGCCAGACAGCACTTCACCAGGCTGCA

CATAACCGACGGCGGACAGTATGTTGCATGCTGGTGGCAGCGGGTGCATCCCTAACCATCAGGGATCTGC

AGGGCAATACGCCGAGGGCTCTTGCCCAGCGTGCAGATGACCAGGAGCTGGCTGCCTATCTGGAGAGCCA

GGAGCAGTTTCAGACCATGGTTCATGAGGACTTGGAGACAACCGTA

>MN110020 Procambarus clarkii 1-phosphatidylinositol 4,5-bisphosphate phosphodiesterase delta-4-like mRNA, CDS

ATGGAAGACGTAGTACAGAAAATCCACGAAGGTAGCACACTATGGAAGGTTCGTGGTGTC

AACAAATGGTTCCATCGTCATTACAAAGTTGACATTGACAACATGAGCCTTGTTGGGGAA

TCTAAGAAATGGTGGTCCCCTGGTGGGGGTACAGGCAGCGATGGACCAGAAAATGCAGTT

CCTTTGATAGAGATCACAGAGGTCCGTGAAGGCTGGAAGACGGACACATTCAACAAAGTT

AGTAGCCACTCTGAGAAGACCAAAGATAAAGCGGCAGCTGGCGATGAACCGTCATTAGAA

GAAAACAAGTGCTTTTCAATCATCCACGGACCAGGTGGGAGGGAGGTGTTGGACCTGGTT

GCAGCTACTGAAGATGAGAGGGATGCTTGGGTGTCGGGTCTGACACACCTTGTTCAGTCT

GTCAAGGCTCTTCATGAAGAGAAACAGTATGATGTGTGGTTACGGCAACAGTTCGAAGAA

GCTGACAAAGACAATAATGGATCTCTTAATTTTAATGAGTGTTGCACTTTGCTCAAGCAG

TTAAACATCAAAATGGACAAAACACATGCCAAGAAGCTATTCAATCAAGCTAACACCAGT

AAGCAGAAGAGAGATGGCGACCAAGTTCTGGAAGTTGGAGAATTTGTTAACTTCTATCAT

GCACTACTGAAAATGCCAGAAATTGAAAAGTTGTTCAAAAAACATGCAAGTGAGAAAACT

CAGCTAATGAGTGCAGACCAGCTGTGTGCCTTCCTCCATAAAGAACAGGGTCTCATTGAC

TTGGATGAGAGCCAAGCAACCAAGCTCATTCATGAATTTGAAATCAGTGACCTTAAAGAC

CAAGGATATATGACGCAAGATGGCTTCTACCATATGCTCCTCTCGGATATGTTTGATATA

TTCAACCACGAACACAAGAGCAAAGTTCATCAAGACATGACCCAGCCACTTGCACATTAC

TACATCTCTTCTTCCCATAATACGTACCTGACTGGTCACCAACTTGCTGGAGAAAGTAGC

GTTGAGGGCTACATAAGTGCACTTAAACGAGGATGTCGGCTTCTGGAATTGGATTTGTGG

GATGGAGACGAAGGAGAGCCAATAATCTACCATGGTCATACCTTGACCACTAAAATAACT

CTTGCTGATGTTCTGAGAGATGGTATCAAGGCCTATGCTTTCGAGGCCTCGCCTTACCCA

GTCATTCTCTCTATTGAGAATCATTTAAGTGTGGAACAGCAGAAAGTCATGGTGCAACTG

CTGAAGGATATCCTTGGAGATATGCTTGCAACTGATCCAGTGTCAGAAGACATTACAGCT

GTCCCCTCTCCAGAGGCGCTAAAAAACAAAATAATCATCAGGGGCAAAAAACCTCAAGGG

TCTGAAGTGGAGTCGGATGATGACGATGATGACCAGGATGCCATTGATTATATTGATAGT

GATGATGGTGCTGCACAAACTAAAAAGAAGCCACACAAGCCTCTCGCTCCAGAATTAAGA

GAGATTATCAATGTATGTGAAGGGAAAAAGTTTACTACTTTCCATGATGCCTTTGAAAAT

TATAAATGTGTTCACACTCCATCGCTAGGAGAGACGAAAGCAAAAGGATTAATTGAAAGC

AGTCCAGATGACTTCATTAGGTTCACTGAGAGGCACGTTACCAAGGTGTACCCCCTTGGT

ACCCGGACAGACTCTTCTAATCTGAAGCCGTACCCCTTCTGGAGTGTAGGATGTCAGATA

GTGGCGCTGAATATGCAGACAGAAGACAAGGCCAACTTCTATGGTGATGCTCTCTTTACC

ACCAATGCAAATTGTGGCTATGTTCTCAAGCCAGATATTATCCTTAAAAAGGTACCCTAT

GATCCGACAGACCTCTCAGACAGATACACCAAAATTGTTCGTGTTACGGTGTTGAGTGGC

CAGCATCTACCAAATTCGGCCAAAAAGGGTGACATTGTTGATCCTTATGTACAAGTAAAA

GTTCGAGGTCACCCGCTTGACAAACAGAAACAGCGTACTAAAGTTATTAAAAACAATGGT

TTCAATCCAGTTTGGAATGAGGCATTAGAGCTGTCCATCAAGGTA

>MN110019 Procambarus clarkii cAMP-dependent protein kinase catalytic subunit 1 mRNA, CDS

ATGGGAAATGCGGCCACAGCCAAGAAAGGCGACCCTGCAGAGAATGTCAAAGAGTTCCTC

GAAAAGGCGAAAGAAGAATTCGAAGAAAAATGGAAAAGTCCCACAAAGAACACGGCGTGC

CTGGACGACTTCGAGCGTCTCAAGACGCTGGGGACGGGTTCCTTCGGGCGGGTCATGCTG

GTCCAGCACAAGGCCACCAAGGAGTACTATGCTATGAAGATACTCGACAAACAGAAGGTG

GTTAAGTTGAAACAGGTGGAACACACGCTGAACGAGAAGCGCATATTGCAAGCCATCACC

TTCCCCTTCCTCGTCTCCTTAGAGTTCCATTTCAAGGATAATTCCAATTTGTACATGGTG

TTGGAGTACGTGCCGGGTGGCGAGATGTTCTCTCATCTCAGAAAGATTGGCAGGTTTAGT

GAGCCTCACTCTCGGTTCTATGCGGCCCAGATCGTGCTAGCATTTGAATACCTCCACTAC

CTTGATCTCATATACAGAGATCTCAAACCAGAGAACCTACTTATAGACAGCCAAGGATAC

CTCAAGGTGACAGACTTTGGGTTTGCCAAGCGAGTCAAGGGACGAACGTGGACGCTGTGT

GGAACCCCAGAGTACCTAGCCCCAGAGATAATTCTTTCTAAGGGCTACAATAAGGCAGTG

GACTGGTGGGCCTTGGGTGTCTTGGTCTATGAAATGGCTGCTGGCTACCCTCCGTTCTTT

GCTGACCAACCCATCCAGATATATGAAAAGATTGTCTCGGGAAAGGTGCGGTTCCCAGGT

CATTTCTCTTCTGATTTGAAGGACCTACTAAGGAACCTCCTACAAGTTGATCTAACTAAG

CGGTATGGAAACTTGAAGAATGCTGTAAACGACATTAAGAATCACAAGTGGTTCGCATCG

ACAGATTGGATCGCTGTATATCAGAGGAAGGTTGAAGCTCCATTTATACCTAAATGCAAA

GGACCTGGAGATACTAGCAACTTTGACGACTATGAAGAAGAAGCCCTTCGTATCTCCTCC

ACAGAAAAATGTGCCAAGGAGTTTGCCGAGTTTTAG

>MN110035 Procambarus clarkii protein kinase C-like mRNA, partial CDS

ATGTTCACGGGCAACATTAGAGTGAAAATATGCGAAGCTGCAGATCTGCGCCTTACCGAC

TGTATGACTCGTTATGTCGGAGTGGCAGGGGTTGGAAAGGGCCCACAGGACCAAACCCTG

GATCCCTATGTTAGCTTAGATCTTGACGAAGTGCACTGGAACAAGACCCAGCCGCGGCAG

AAGACGTTGACGCCCGTGTGGAACGAGTGGTTCGAGCATGAGGTGGTCGGGGCCGTCCAA

CTCGGCCTCAAGATCTTCCATGATTCTCCGGTCGGCAACGACGACTTCGTAGCCGATGCC

ACCGTGCCCTTCGAGGAGATATGTGCCGATAATCAGACCCACGCCGATATATGGGTGGAC

CTAGAACCTCAGGGGAAGCTACATGTTGTAATTGAATTAAAATGGGCCGAACCAGAAGAT

GGAAGTATCCGCCCACGAGAATTCAGGGAACGGCAGGGTTTCAACCGCCGACGTGGAGCG

ATGAGGCGGCGAGTTCATCAGGTCAATGGTCACAAGTTCATGGCTACTTTCCTACGACAG

CCAACCTTTTGTTCGCACTGCAGAGATTTTATATGGGGTTTAGGGAAGCAGGGTTACCAA

TGTCAAGTATGCACCTGCGTTATTCACAAACGTTGTCACCAGTCTGTCGTCACACGATGT

CCAGGAAGCAAGAGTATAGACCAAATCTCAGAGGAGCCATCTGTAAAGGGAAGTGGTCAG

CAACGGTTCAATGTCAATGTGCCGCACCGGTTTTCTGTTCACTCATACAAGCGGTTTACG

TTCTGCGACCATTGTGGCTCATTGCTTTATGGCCTTATTCGTCAGGGTCTCCAGTGTGAA

GTATGCAATATGAATGTTCACAAGAGATGCCAGAAGAATGTAGCCAACAATTGTGGAATA

GATGTAAAACAATTATCTGAAATCCTCACAGCCATGGGAATACGTCCTCAAGATGAGTCA

GGGAAACGTAAAAAGAAGTCTATCAGTGAGCCATCGAAATTACCTGCATCAGCCACAGCC

TCTGTGAGTGTTCCAGGAAGTATCAGTGAGATAGAGGAGCTAGGAACAATGAAGGAGGAA

GAACTAAGACTACGGATTGAAGCTCAAAGATTTATGGAAGAGAAAATGAAAGATCGATGT

CCAGAGGAAGAACAGGATTTAAAGCCGAGAACACTAGACACTGACTTAGACATGGTGCTC

GGTGGGAAGAAGCTCGGGTTAGACGACTTCAACTTCATCAAAGTACTGGGCAAGGGAAGC

TTTGGCAAAGTTATGTTAGCTGAACTAAAAGGAACGGATGAAGTTTATGCCGTCAAGGTG

CTGAAAAAAGATGTCATCCTTCAAGATGATGATGTAGAATGTACAATGACTGAGCGACGC

ATCTTAGCCCTGGCCGCCCACCATCCTTTTCTCACCGCCCTCCACTCTTGCTTCCAAACT

AAGGACCGG

>MN110013 Procambarus clarkii beta-arrestin-1-like mRNA, CDS

ATGGACGACAACAGCAAGCGGCAGGGGACCAGAGTATTTAAGAAAAGTTCTCCGAATGGA

AAAATCACCGTCTACTTAGGGAAGAGAGACTTTGTTGACCACATCACACACGTAGACCCA

ATAGATGGCGTAGTGTTGATCGACCCCGAGTATCTGAAGGACAGAAAGGTATATGGACAT

GTCTTATCTGCCTTTCGGTATGGCCGCGAAGACCTGGATGTGTTGGGCCTCACATTTCGC

AAGGACCTGTACCTAGCGTCAGAGCAGATCTTTCCCCTTGACACTGCCAGCAAGAGGGCC

CTCACTAGGTTACAGGAACGCCTCATCAAGAAATTAGGACCAAATGCCTTTCCGTTCTTC

TTTGAGCTTCCCCCTCACTGCCCAGCGTCAGTCACGCTGCAGCCTGCACCGGGGGACATG

GGCAAACCCTGTGGGGTTGACTATGAACTTAAGGCATATGTTGGGGACAGTGTCGATGAC

AAACCTCATAAAAGGAATTCAGTGCGGCTTGCAATTAGGAAGGTTATGTATGCTCCCATG

AAGCAGGGAGACCAGCCCAGTGTGGAGGTCAGCAAAGAGTTCATGATGTCTCCCAACAAA

CTTCATCTTGAAGCATCTTTAGACAAAGAGTTATATTATCATGGAGAAACAGTAGCCGTT

AATGTCCATATACAGAATAATTCTAATAAGAGCGTCAAGAAAATTAAAGTATCAATTCGG

CAGTTTGCGGACATATGCCTATTTTCAACGGCTCAGTATAAATGTACAGTGGCTGAAACG

GAGAGCGAG

>MN110014 Procambarus clarkii phosphatidylinositol transfer protein beta-like mRNA, partial CDS

ATGCTCGTCAAGGAATTTCGAGTCATCCTTCCAGTCACAGTTGAAGAGTATCAAGTGGCA

CAGTTGTACAGTGTAGCTGAAGCGAGTAAAAATGAAACAGGTGGAGGAGAAGGCATTGAA

GTTCTCAAGAATGAGCCCTTTGACAATTATCCATTACTTGGGGACAAGTACAGCAAGGGA

CAGTACACCTACAAAATCTACCACCTTAAAAGCAAAGTACCATCTTTCATCCGGATCCTA

GCACCAGAAGGGTCACTAGAGATTCATGAAGAAGCATGGAATGCCTACCCCTACTGTAGA

ACCATCATCTCGAATCCAGGCTACATGAAGGAGAGTTTTTATATTACTATAGAGACACTT

CATGCAGCTGACGATGGGGAGTCCGAGAATGCTCACAAACTGACGGGAGAAAAGCTGAAA

ATACGCGACGTAGTGACAATTGACATTGCCAATGATCCAGTTAAATCTTCAGATTATAAA

CCTAAAGAGGACCCAACAAAGTTCAAGTCTGAGAAAACTGGCCGTGGTCCTCTGAAAGGT

CCACAGTGGTGGAAGAAGTGTGATCCCGTGATGACATGCTACAAGTTGGTGACATGTGAA

TTCAAGTGGTTTGGGCTGCAGGCACGCGTAGAGAAGTTCATCCAGGATGCTGAGCGCAGA

CTGTTTACCAACTTTCATCGACAAGTGTTTTGTTGGATGGACGAGTGGCATGGCATGACC

ATGGATGATATCCGGCGGTTAGAAGATAAAACAAAGGAAGAACTGGATCAGCAGAGACAG

ATAGGAGAAGTTCGAGGCACAATGGATGAG

________________________________________________________________________________________________

SET 4. Opsins

>MT601688 Procambarus clarkii G-protein coupled receptor moody-like mRNA, partial CDS

GCTAACTTGGCGAACGTCACCCATGGCAGGGAGCCGCTCTTCGCAGACTATCCTCCACCT

CTCTTAGACTTCGCGGTGTTCTGCTGCGTTCTGTTCATCGTTCTGGGTGTCCCCGGTAAC

CTGATCACCATCATTGCCCTCGTCAAGTGTAAAAAGGTCCACAACGCTACCGCTGTCTTC

ATCATCAACCTGACGCTCTCCGATCTGATGTTCGGGGTGTTCAACCTGCCTCTTGCTGCC

TCGTTGTTTGGGCATAGAGCCTGGGTACATACGGGCTTCCTGTGCCTACTCTTCCCTATC

CTGCGCTATGGGCTGGTGGCAGTATCTGTCTTCACCGTTCTGGCTATCACTATCAATCGT

TATGTTATGATTGCTCATCCCAGACTGTACCACAGGCTGTACACTCGGACGTGGTTGGCT

ATCATGGTCGTGGCGACGTGGATAGGTGCCTTTGGAGCCCTGGTGCCTACGCTGCTAGAA

TTCTGGGGCACCTTTGGCCTGGACCCGGCGATTGGCTCCTGCACCATCTTACCCGATCGG

GACGGAAACTCGCCCAAGGAGTTTCTCTTCGTCTTCGCCTTTGTGCTGCCCTGTGTGGCC

ATCTGCGTCTGCTACGCCCGCATCTTCTGCATCGTGCATAGAGCGGATAAGAAGAGCCAC

AGCCACAGTCAGCATTTTCTCAAGGTTAACGGCAAGCGTCACAAGAATGTTGCTTCGCCT

TGTACGGCTGATCCACCGACGTTGCAGACCCTTCATGCAACTCCGCTACACGATCGTCTC

CAGCTTGAGACGCTCGTCAAGCTCTCCACCAGCAGTAATTCCGTTAAGGTGGAGATAGTT

GAACCACAGGAGGAGTCTCAGAAAAATGAGGAGAGCTCGTTAGACGGTAATGGAAGGGGG

GGCTGTGGGAGCAAGGAAGGCACGCGGACGCCATCTCCTGAAACCACTCCTGCAGGAATG

ACCACTGACGCCAGTTCGTTGCTGAAGACGGAAGCCAATGGCTTTTCCGGTCATTCTTCA

AACGAGGGAGTTGGCGCCTCGTCAGCGGCCTGTTCTCCTATCCGGATAAGTCCTCCAACG

GCGCCTGAATCTCCCGCTTCAGTGAAGTCGGATAGAAAAATAAGTACCAACATCGAGAGA

CGGCCGTCCACTATATCCGGCCGTGGTGGCACTTTCAGCCACCTCCGTGGCACTTTCCGA

AGGACACGCGCCGGTTCCTTCATTGCCAGACAACCTACATTGAGTGCCAAGGACCGGCGT

CTCCTGAAGATGATCCTAGTGATCTTCTTGTCATTCGTAGCGTGTTATCTACCCATCACG

CTCGTCAAGACCTTCAGTAAGGATGACAACCCCGTGCTAAACATCCTGGGTCTCCTCCTT

ATCTATCTAACCACCTGTATCAATCCTATCATCTACGTGGTGATGTCTTCGGAGTACCGT

CAAGCCTACGTCAGCCTCCTGACCTGCAAGCGTGACCACGATCCCGCTAATCGCTCCGTC

ACTAAAATTTCCTGA

>MT601689 Procambarus clarkii G-protein coupled receptor moody-like isoform X2 mRNA, CDS

ATGGAGACCCTCAGGTCGTCTGCAGACTACCCCGACTATCCTCATTTCTTCAACGACAGC

TGGGACGAGCCCCCTTGGGACGAGGCGGCCGAGTCAGAGATTGCCAAGATGTCGCGGGGC

GTGGCCAGGGTCATCGCTGTGCTCTTCATAGGCTACATGGTGTTGGGGCTGACTGGCAAC

TTCCTCACTATCCTGGCCCTCCTGCGCTGTCCTCGGGTCAGGAACGTCACCGCTGCTTTC

ATTATCAGTCTGTGTGTGGCCGACTTCCTGTTCTGCGTGTTAGTACTGCCTTGGGAGGTG

TCAAGATTCCTTGCTGGCAAGTGGGTCTGGGGAGAAGGCTGGATCTGCACCCTTTTCCCG

CTTCTCAGGTACTGGAACGTTGCCGTCTCTCTACTGTCCATCGCCATGATCACTATCAAC

AGGTACATCATGATAGCCCACTTCAGCGTGTACAAACTGGTGTACAGGAAGGGTTGGATC

GCCCTTATGATCGCCTTCTGCTGGGTCTTCGCCTTCGTCATGCTTCTCCCTACCCTTCTC

AGCAAGTGGGGACGCTTTGGCTTGGACCGACGGCTACAGACCTGCTCAATCCTGGACGAC

AGCAACAAGTCGCCCAAGCAGGTGCTCTTCGGGTTGGGCTTCTGTGTTCCCGCCATAGTC

ATCGTCATCTGCTACTCCCTGATATTCTTCGTCATCCACAAGTCTGAGAAGCGGATGCGT

CAGCACAGCACCCGAGGCATGAACGGGGCGGCGCCCCCCTCCGGACCTACCCTCCAGCCA

CAGTCCAGAGTCACCACCAAGGTGGAGCGAGAGGCCCGGCGCCGCAGGAATGAGTGGAGG

ATCACCAAGATGGTGCTCATCATTTTCATCGCTTTTCTCATCACCTACCTGCCCATCACC

CTCGTTAAGAACCTGGACAAGAAGGTGGATTATCCAGGGCTCCACGTGCTCGGCTACGTA

CTTATCTACATCTCGGCGTGCATCAATCCTGTCATCTACGTCATCATGAACAGACAGTAT

CGACAGGCCTACAAGACGGTGCTGTTGTGTCGACGTCCCCGTCTCCCGTCTCTCACCTCC

TCCCACACAGAGCGCGGGAAGGGTCGGAACAAGATGGTGATGGAGGAGCTGAATGATAAG

ACCATGATGTCTCAAGTCTCTCTCTCCGACGCCGGCCCCTTGCCCGAGTGCCACGAGCTT

CCTGAGGTCTTCCTCGACAAGTGA

__________________________________________________________________________________________________

SET 5. Phototransduction, Ciliary

>MN110027 Procambarus clarkii neurocalcin homolog mRNA, partial CDS

ATGGGCAAGCAGAACAGCAAACTCAAACCAGAAGTGTTGGAAGATCTTCGTTCCAATACA

GAGTTCACAGACGCTGAGATCCAAGAATGGTACAAAGGTTTCCTTAAAGATTGTCCAACA

GGCCACCTTTCAGTTGAAGAATTCAAGAAGATATATGGGAACTTCTTTCCATATGGCGAC

GCATCAAAGTTTGCGGAGCATGTGTTCCGGACCTTCGATGCAAATGGGGACGGCACCATA

GACTTTCGAGAGTTCTTATGTGCCCTTTCTGTCACATCAAGAGGAAAGCTGGAACAGAAG

TTGAGGTGGGCCTTCTCAATGTATGACCTCGATGGAAATGGATACATCAGTCGACAGGAG

ATGCTGGAGATCGTTACAGCAATTTACAAGATGGTTGGCTCTGTGATGAAGATGCCAGAA

GATGAAAGTACCCCTGAAAAACGTACAGACAAAATCTTTCGTCAGATGGATAAGAATAAG

GATGGCAAGCTAAGTCTTGAAGAATTCATCGAAGGGGCTAAGAGTGACCCATCCATCGTA

CGCCTGCTGCAGTGTGACCCTCAATCATCTCAG

>MN110022 Procambarus clarkii neuronal calcium sensor 2-like mRNA, partial CDS

GGGTGTTTCGGGAGCAAGGAGAAGCTCTCTAAGGAGGACCTGGAATTCCTCAAGACGCAC

ACCAGATACGACGAACAGATGATCAAGGAATGGTACAAAGGCTTCAGGCAAGACTGTCCG

AGCGGCCGTCTTACCCCAGACAAGTTCGTCGACATGTACAAAATGTTCTTCCCATCCGGT

AATGCGGAGGAGTTTTGCGACCACGTCTTCAGAACCTTCGACATGGACAAGAACGGTTAC

ATAGACTTCAAGGAGTTCCTGCTGGCCATCGACGTGACGAGCGCCGGCACCCCGGAGGAG

AAGCTCAAGTGGGCCTTCCGCATGTACGATGTTGACGGCAACGGTGTTATCGACGTCTCC

GAGATGACCAAGATTGTTCAGGCCATCTATGACATGCTTGGGACCAACTCTGCTAGTCGT

CCAGCGGACAGTGCTGAAGAGCGAGCAAAAGCCATTTTCTCAAAGATGGACGAAAATAAT

GATGGCAACCTAACTCAGGAAGAGTTCCTGAAAGGGTGCTTGCTAGATGAGGAGCTCTCT

AAGATGCTCACACCGGGAGCT

>MN110033 Procambarus clarkii regulator of G-protein signaling 9-like mRNA, partial CDS

GAGCGTCTGGAGATCGAGGACTCAGCGGAGGCCATCCACCTGGCCAACCTGCTCTGTCAG

TACGGCTACTTCTTCCCAGTGGGGGAGTCTCGTTCCCTCATCGTCAAGGATGACTCCTCC

CTATACAGGTTCCAGACGCCGTACTACTGGCCCTCTCAGAACCACAGCGCCGACACCACC

GACTACGCCATCTACCTCACCAAACGACTCTCCAGGAACAAGCAGAAACATGGCCTGGAG

GACTACGAGGTGGACGCGTACAACAAGCTGAAGAAGGCGCTCTCCCACAAGTGGGACTTT

ATCTCCATGCAGGCGGAGGAACAGGTAAAGCTAGCGAAGGACAGGAAGAAGGGTGACAAG

ATAGTGACGGACTCCCAGGAGCGGGCCTACTGGCGGGTCTACCGTCCTCCTCCGGGCTTC

ACCAACTGCCTAGAGACCGCCCCCGTCCCGGACAAAACAAATATGGCCAACAGAGTCCGC

AAGAAAACGGTCGACGATCTCAAGAAA

>MN110036 [organism=Procambarus clarkii] regulator of G-protein signaling 7-like mRNA, partial CDS

GAACAATATCAAGAATATGATCCTTTCATCCCTGCAACTGACCCGCCTAATCCCTGGGTT

TCAGACTGTCCTGATTTCTGGGAAGCTGAGAAAATTGCGAAGGAAATCCCATCTAAACGA

GTAAGAAGATGGGGCTTCAGTGTACAAGAACTGCTCAAAGACCCACTTGGACGTGAGCAG

TTTGTCAGATTCCTTGAGAAAGAGTTCAGTGGAGAAAACCTAATGTTCCTTACTGCCGTT

CAGGAACTGAAGTGCCTCCCACAAAAGGACGTCCATGATAAGGTCCAAGCTATCTGGGAT

GAATACCTCGCCCCTAGTGCCCCTGTGCCCGTAAACATCGACTCTAAGTCCATGAATATT

ACAAAGAAAAACATG

>MN110028 Procambarus clarkii putative regulator of G protein signaling mRNA, partial CDS

TTCAGGAAGGACAAAAGACTCAGTATGATTATGGCAGCTGGTTTAGATCCAGGCAAGCCT

CCAGCAGCCACTAGCAGGTCCTTGGAGCATCTAGCGGTTGATGGTGGTGTTGAAAAGAAG

TGGACTCGAAGCACCTCTCTCCGCCGTCACTTCCACAGTTCAAGACACAATGGTAGCCAC

CAACAAGATCCTTCAGCTCACTCTGATACTGAAATTGGCAAGATATCGGCACCACACAGC

CTCCTAGATGGTGAATGTGGTTCTGTAGGAGGAAGCGTATCCTCTGAGAGAGCTATTGAC

GTTGGAAGAGTGGGTGCTTGGGCTACATCATTTGAGAAGCTCTTAGAAGATCCCGCAGGG

CTGCATACCTTTGCTGAATTTTTGAAAAAAGAATATAGTCATGAAAACATTTACTTTTGG

ACGGCTTGTGAACGATACAAGCGAGTCTCCAACCCTGATGAGCTTCGAGCCATGGCCAAG

GAGATTTTTGAGAGACACTTGTACAGTGGCGCCCCTGAACCTGTCAACGTTGACTCGCAA

GCAAGACAGGATGCTGAAGAAGGCCTTCATACTCCAAATCAATTTTTATTTGATCAGGCC

CAGAAACAGATTTTCAATCTCATGAAGTTTGACAGTTATTCTCGATTCTTGAAGTCAAGT

TTATACCAAGATTGTGTATCTCGGGATATGAGAGGTCAAACTCTGCCCTACCCTGGGGAT

GATAACCTTGACCCGGATCTAAGAATTGCACAAGAAGATTCACATGTAAAGTTGAAAAAA

AGCAGATCAGATGCAGAGGAACGTCGTCGGAAGTCATTATTACCGTGGAATCGCAAGGAT

CGTAGTAAGAGCAAAGACAGA

_______________________________________________________________________________________________________

SET 6. Melanin Synthesis

>MN110038 Procambarus clarkii cysteine sulfinic acid decarboxylase-like mRNA, partial CDS

ATGAACGGTGGACCCACTGCGCTTAACAGCCACAAGAACAATGAATCTACTACGCTTAAA

CAACCCACCACCAACGGCTACGTGACATCAGAAACCAGTATACAGGAGGAGGACGGACTA

GACGAGCGAGGGGCCCTGCTAAAAATAGTGCTAGACATCGTACTGAAGGCCAACCTCGTT

ACCGGCATCAACCCCACGGAAAAAGTTGTCGAGTTCAGACACCCCAAGGAACTAAAGGAA

GTGCTGCAAGTTGGGGTGGGGGTGAACGGGTGTTCCCAGGAGGAGATGGAGGCCGCGCTG

GAGCAGGTTGTGCACTACAGCGTCAAGACTCAACATCCTCACTTCTACAACCAGTTATAT

GGAGGCATCGACGAGGTGGCGCTCACTGGAGCCTGGATGACAGAGGCGCTCAACACTAAC

CAGTACACGTTCGAGGTGGCCCCGGTGTTTATGTTGGTGGAAGATTACGTCATCTCCAAG

CTGGCCAACCTTTACGGATGGCCCAATGGTGACGGTATCTTCGCTCCTGGTGGAAGCTTG

AGTAACATGTACGGTATGGTGATGGCCAGATACAAGAAGTATCCAGACGTGAAGAGGACC

GGAGTGTTTGGCCTCAAACCCCTCGTGGCGTTCACCTCAGATCAGGGACATTATTCAGTG

AGCAAGGGCGCCTCGTGGCTGGGTGTTGGGATGGACAATGTTGTAAGTGTTGCTTCAGAC

AGTCAGGGGAGGATGAAGGCGGAAGAGCTGTCTGCGCGGGCGACGTATCTGTTCCAGCAA

GACAAACACTACGACGTGAGTTACGACACTGGCGACAAGAGCGTCCAGTGTGGGCGCAAG

GTCGACGCCTTCAAGCTATGGGTCTTCCTAAAGTTCCACGGCTTGGACGCCCTCGAGAAG

AGGGTTGATGCTGCCTTCAGCGCCTCCAGGTACTTGAGTAAGAAGGTAGCCGGCCGTCCT

GGCTTCAGGCTGGTACAAGAGCCACAGTGTACCAACGTCTGCTTCTGGTACATCCCAGCG

AGCCTCAGGGGCCTCCCAGAGACCCCAGAGTGGTGGGTCAAACTCTCAAAGGTTGCTCCA

GAACTGAAGGCGCGGATGGTGAAGCAGGGAACTATGATGGTCGGGTACCAGCCCATCGCC

TGCAAGAATCTAGTCAACTTCATCCGGATGGTCACCACCTGCACGCCTACGCCCACACAC

GAGCACATGGACTTCGTAATTAGTGAGATTGAGAGACTGGGAGCCGATCTG

>MH156427 Procambarus clarkii prophenoloxidase (proPo) mRNA, partial cds

ATGGCTAACGTTCAGGCGCAAATGCTTAAACTGTTCGAGCGGCCGTACGACCCTATGAACTTGCGGCGAA

GTGATGTTCCCACAGGATCAGCAGGCACCGTGGGGACCAGGTTCGGTGGTGCGACGGTCCCATCACTTTC

AGATGCTGATAAGAACCAACTGGGAAAGGCTCTCTCCGTCCCTCGCGGCAGCGTTTCTCCTTCTTCATA

AGGTCCCATCGCGAGGCCGCCAAGGACCTCTGTGCTTTCCTGATGAAAAGTACAAACGCCAGTGAACTGA

TGCAGTCGGCGGCCAAAGTGAGGGAAGAGGTCAATCAGTCTCTCTACAATTACGCCCTCTCCTTCACCAT

ACTTCACAAGCAGGATTTGCGAAATGTTCGCCTGCCGGCAGTCGTGGAGGTCTTCCCTCACAAATTCATA

CCACAGGAAGAACTGACAAAAATGCAGATTGAAGTGAATCGGACTCCATCTACTGCGACAACACCGCTGG

TGATTGAACACGGAGCAGACTTTGCCAACACCACCCTGAAGCCAGAGCACCGAGTGTCATACTGGAGGGA

GGACTACGGCATCAACTCCCACCACTGGCACTGGCATCTTGTTTACCCCGCTGGCATGAATGTCAACCGG

GACCGTAAAGGAGAACTTTTCTATTATATGCATCAGCAGATGGTTGCCAGGTACGACATGGAGCGGCTCA

GCGTTAACCTCAAACGTGTGGAGAAGCTGGAGAACTGGCGCGAGCCCATCCCAGATGGTTACTTCTCCAA

GCTTACTGTTAACAACTCCGGTCGGCCCTGGGGCATCCGTCAGGACGGTACCTTCCTGAAGGATTTGAGA

CGTAACGATGCCGGGATTGATTTTTTGGACATTAGTGATATGGAGCTCTGGCGGTCCCGACTGATGGACG

CCATCCACCAAGGATATATGCTAAATCCGAATGGTGAGCGCATCCAACTCTCTGATAACGTCACGACAGG

AAAGCGAGGGATTGACATCTTAGGAGACGCATTTGAGGCAGATGATAGGCTGAGCCCACATTACCTCTTC

TATGGCGACCTTCACAACATGGGCCACGTGATGCTGTCATTCTGTCATGACTTCGACAACGCACATAGGG

AGGAGATGGGAGTAATGGGAGACTCGGCGACTGCCTTGAGAGACCCAGTCTTCTACCGCTGGCACAAGTT

TGTGGACGACGTCTTCCAGGAGTACAAGCTGACGCAGCCGCCCTACACCATGGAGGACCTGACTCTGCCG

GGCGTGGTGCTTGACAAGGTGGGTGTTGTGAGGGACAACCAGCTCAACACTCTCACAACTGGC

__________________________________________________________________________________________

SET 7. Pterin synthesis

>MN110003 Procambarus clarkii Aldehyde oxidase mRNA, partial CDS

ATGGAGGCTCATGCTGGTCGGGTTGTGCCAACAGAAGATGGATATAATGTGTTCAGTACA

TCTCAGTGGCCAACAGAAACACAGGCAACTGCGGCTCAGGCACTGGGCATATCGACCAAT

AGCATCAATGTGTCTGTGCGTCGAATTGGTGGTGGTTACGGATCCAAGATTAGCCGTCAG

CATATAGTTTCGACAGCAGCAGCAGTTGCAGCCAGAAAACTGAAACAGCCAGTGCGTGTT

GTGATGGACCTCACTGCTAATATGACTTATGCAGGCTGGAGAGAACCCTATTACTCAAAG

TATGAGGTTGGCTTTGATGGTAAGGGGAAAATTGAAGCTCTGAAGATTGAATTATATGGT

GATGCTGGCCATATCTCCAATGAATCTGCTGTAGGGTTCTTATTTGCTGCCATCCAGAAT

TCCTACTATATTCCCAATTTCAATTTTACACCTGCCAATGTGAATACTGATACTGCTGCC

AATACTTACTGCAGGACACCTGGCCATGTGGAGGCTGTTGCTACAATAGAAAACATCATG

GAGCATATAGCAAATTATTTAAAGCTTGACAAGTTGGAAGTGCGACTTATTAACATGGTC

CCGCCTCAAGTCCCTCGCATGAAAATTCCACCACACGAGAGGAATGTTGTAAAGGAAGAT

ATTCTTCCATTGCTGATGAAGAAGGCTTCAGTCGTGCAGCGGCAACAGGAGGTGGATACA

TTTAACATGAACAACAGATGGAAGAAACGAGGACTGTCTGTTCTTCCATTGTGGTATGGG

TTTGACTACCCATCAATGTTCCGCTACGGTATGCAAGTCACCATTTATGAACATGATGGT

ACAGTAGCCATCTCTCATGGAGGGATTGATATGGGACAAGGAATTAACACAAAGGTGGCT

CAAGTTGCAGCGTACGTCCTGGGCATACCTCTGGAGCAGGTTGTGATCAAAGCCTCCGAT

ACAATGGTGGGGGCCAACTCCATAGTGACTGGGGGCTCCTTTGGATCAGATCTCTGTGCT

CATGGTGTGAAAATTGCCAGTACAGCCCTTCGTCAGCGTATGGATGTCATAAAGGAAAAA

ATGAAATCAGAGACGGGAAAAGATCCGTCTTGGCTGGAGCTGGTTAAAAAGTGTCACGCT

GAAGATGTGGACCTGTCAGAGCGTTACTGGACTGCTGGCAAGGAGCACCCAAAGCGTTAC

GACATCTGGGCCGCTTGCTGTCTTGAGGTTGAAGTTGATGTTCTGACTGGGGTATACATG

ATTAGACGTGCTGATCTGATAGAAGACAGTGGCCGAAGCATGTGC

>MN110004 Procambarus clarkii Indole-3-acetaldehyde oxidase-like mRNA, CDS

ATGACCTTAGTGGGCTGGCGAGAGCCATACATGTCTAAGTATGAGGTTGGGTTTGACGAC

GCGGGCAGACTGGAGGCGGTGAAGGTGGACATGATCTCCGACGTGGGCCACGTGGGCAAC

GAGGCCTCCGTGGGCTCCCTAGCCAGTCGGCTCCGCAACACCTACTACCTTCCCAACGTC

TTGTTTCGCCCCGTCATTGTCCGCACCAACACGGCCGCCAACACCTGGTGCAGGACGCCA

GGTACTGTGGAGGCTATTGCGACCATGGAGAATATCATTGAACACGTGGCTAGCTATCTG

AAGAAGGACCCACTTGAAGTGCGACTTGTCAACATGGTGGCTCCAAATGTGGCTCGACTT

ATGACTCCACCTCATGTGAGAAATGTGGTCAAGGATGATATCTTGCCACAGTTGATGCAG

AAGTCTATGTACGAGCAGAGGCAGGAGGAGATCCAGATATTCAACCAGGAAAACAAGTGG

AGGAAGAGAGGCATGTCTATTGTCCCTCTGTGGTACGCCCTCAACTATCCTTCAGTGTTC

CGGTACGGCATCCAAGTGTCCATCTACGAGCACGATGGCACCGTTGCTGTCTCTCACGGA

GGCATCGAGATGGGGCAGGGCATCAATACTAAGGTGGCCCAAGTTGTTGCGTATGAACTT

GGCATCCCACTGAGCTCTGTTATACTGAAGGCATCTGACACCATGATTGGCTCCAACTCT

ATTGTCACGGGCGGGTCTTTTGGCTCAGACTTGTGTGCTCATGGAGCGAAAGTAGCGTGT

GAGGCCTTACGTAAACGTATAGACGTGGTCAGGGAGAAGATGAAGAAGGACACTGGTGAT

GACCCGTCTTGGGTGGAGCTCATTAAGAACTGTCACGCTGAAGACATTGACATCTCTGAA

CGGTACTGGACTGCCGGACGGGAGCACCCCGAGCGCTACGACATCTGGGCCGCCTGTTGT

CTCGAGGTGGAGATTGACGTCTTAACCGGACAGTATCTGTTCCGCCGTGCAGATATAATT

GAAGACTGCGGGAGGAGTCTGAATCCGTATGTGGACATTGGTCAAGTGGAAGGAGCCTTT

GTCATGGGCATGGGGCTCTACACATCAGAAATGGTGAAATACGATAGCAGTACCGGCCAA

AAGCTGTCAAATAGCACTTGGGAATACAAACCACCAACAGCTCTGGACATCCCGGTTGAC

ATGAGGATTAGTCTCTTATCTAATACGTCAAACGCTCACGGCGTCCTTGGCTCTAAAGCA

ACTGGGGAGCCGGCGCTGTGCCTCTCCTACGCTACAGTCACGGCTCTCCGCGCCGCCATA

ACTGCATTCAGGGCTGCAAATGGAGACGATGAATGGTTTGACATGGACACGCCAATAACC

GTGGAGAAGGTGCACCAACTGTGTGGAGTGAGACCAGAGCAGTTCACACTCGTCAGCTCC

CTGCACGAGTCCTTCGATGACTTCTGTCTTGTCACCAAGGACCAACTTCCAACAGAAGCT

GCCACCTTCTGCCCTATTAAT

>MN110006 Procambarus clarkii pyrimidodiazepine synthase-like mRNA, CDS

ATGACCACCAAGCATCTCAGTACAGGATCAGCATGTCCTCCACTGGGAGATGGAGTTATG

CGATGCTATAACATGACATATTGCCCATATGCCCAGCGAACACGCCTTGTTCTCGCTGCC

AAAAACGTTACACATGAGATTGTTAATGTTAACTTGATCACCAAACCAGAGTGGCTATTT

GAGAAGAATCCCTTTGGTAAGGTACCAATTCTAGAGCAAAATGGGCGGTGCATATATGAG

TCACTGATAACATGTGATTATTTAGATGAGGTCTATCCTGAGCCACCACTCTACCCAGCT

GATCCTTTGAAAAAAGCAGAGGATCGAATGTTCATCGAGCGCATCTCACAGATAACTACA

CCTTTATACAAGTTATACTATTCAAAGGAGGACGAGCAGACTCAAAAATCCTGCGATGAT

ATCAAATCTGGACTTGATGTGTTTGAGAATGAGCTTGTAAAAAGAGGCTCTGAATTCTTT

TGTGGTGGTAGGCCTGGTATGCTAGATTACATGATATGGCCATGGATGGAGAGGCTTCCT

ATGGTTCAAATGTTTGCTGGAAATGCTGGAATCATCATCCAGGATCGTTTTCCAAAACTG

CTGTCTTGGATGGATACCATGAAGAAAGATGCAGCTGTAAAGGTCTCATTCATATCACCA

GAGACTCACTTCAAGTTTATCAAGACACATTTGAATGGAAGTCCAGATTATGACATGGAG

CAA

>MN110005 Procambarus clarkii Dihydropteridine reductase-like mRNA, CDS

ATGTCTGCTGGTAAGGTTGTGGTCTACGGTGGCCGGGGCGCCCTCGGGGCCGCTGTCGTC

AAGCACTTCAAGCAGAAGGGTTTTTGGGTTGGAACTGTTGACCTGGTAGCCAATGATGAA

GCAGATAAGAGTATCATAGTCTCCAAGGATGGAAATTGGTCAGCACAAGCAGCAGAAGTT

ATTTCTGGATTGGAGGCTGCATTAGGTGAAAGCAAGGTAGATGCTATCATCAATGTTGCT

GGTGGTTGGGCTGGTGGCAATGCTAGCAGTAAGGATTTTCTGAAGAACTGCGAATTAATG

TGGTCCCAGAGTGTGTGGTCATCTACCATCACAGCCCAGGCTGCTTCACGCTTTCTCAAA

GAAGGGGGATTGGTGTCTCTCCCTGGTGCACAACCTGCAATCAATGGAACGCCAGGCATG

ATTGGATATGGTATGGCCAAGGCTGCTGTGCATCAGCTAACAAAGAGTCTTGGTGAGGAA

AAGTCTGGACTACCACAGGGGGCCACAGCAGTTGCCTTGCTTCCAGTCACCTTGGATACA

CCTATGAATAGGAAGTGGATGTCCAGTGCAGACTTCTCCACTTGGACCTCACTTGAGTTT

GTTGCCGAGCTCCTCCACAAGTGGACAACTGGGAGTGACCGACCAGCATCTGGCAGTCTT

GTGCAGCTCATCACCAAGGATAACAAGACAGACTTGGTTGTGGCT

>MN110007 Procambarus clarkii Pterin-4-alpha-carbinolamine dehydratase-like mRNA, CDS

ATGGCACAAAAACTAACAGCAGGGGAGCGAGAGTCAAAACTAAAGCCTCTACTGGACGAC

AGCTGGACAATGGTAGATGGTCGTGATGCTGTGAAGAAAACATTCCTCTTTAAGGACTTC

AATGAGGCCTGGGGATGGATGGGTCGTGTTGCACTGAGAAGCGAGAAGATGGACCACCAC

CCAGAATGGTTCAACGTATACAACAAGGTGGAAGTTACTTGGTCGACACATGATTGTGGT

GGATTGTCTTCCAAAGATATCAATATGGCCACCTTTTGTGATGACACCTTCAAGCTGTCC

AGCAAG

>MN110009 Procambarus clarkii Sepiapterin reductase-like mRNA, partial CDS

CTTGGTACCGCTAGTAAGGAAAATCTAGAGAAGGCCCTGGATAAATTGCTGATAGTAGGACAGGATCCTC

CTCCAACTCGATGTGTCATATTCCACAATGCTGGGTCCCTTGGAGATCTTGTATACCTGCGGAATCTCAA

GGACCTTGACCATGTCAACAGTTATTTCCAAATGAATGTTAGCTCTGTGGTGATGCTGAATGCAGTGGTC

ATAGAAATTATGTCAAAACAGCCTAATGTGGCACTAGAGATTGTGAACATCTCGTCCTTGTGTGCTGTAC

AGCCCTTTAAATCCTGGGGACTTTATTGTGCTGGAAAAGCTGCTAGGGATATGCTCTTTAAGGTTTTGGC

AGATGAGGAACCCAACATTCTGGTGCTTAATTATGCACCAGGGCCCTTGGATAATGAAATGCAGGAGATT

GCTCGTAGAGCAACAACAGATAAAGAACTAAGGTCAACTTTTGCATCGTTAAAGGAGGAAGGCAAACTTC

TTCCCTGTAATGTGTCTGCCAACAAACTCTTGGATATATTGAAGCAACGTAAATTCAAGTCTGGAG

_____________________________________________________________________________________________________

SET 8. Ommochrome synthesis

>MN110008 Procambarus clarkii ATP-binding cassette subfamily G member 1-like mRNA, CDS

GGCAGACATAGAGGTTACAAGACCATTCTGAAGGGTGTAAGTGGGAAGTTCAAATCTGGT

CGACTGACGGCTATCATGGGTCCTTCTGGTGCGGGAAAGTCCACTCTTATGAATATTACT

GCTGGATACAGGATTAGTAATGTTGTTGGGACAATCACTGTTAATGGCAGAGAACGAAAT

CTGCGAAAGTTCCGCAAGATGTCTTGTTACATAATGCAAGATGATCACCTCCATCCACAT

CTCACTGTAATGGAGTCGATGAATGTTTCTGCCAACCTTAGACTAGGTGACAGAATGAAA

AGACAGCAAAAGGAAGAAGTGATCAATGAAATACTGGAAACCCTGAGCTTAACAGAATGC

AGAGATACAAGAGCAATTAATCTTTCCGGTGGCCAGCGCAAAAGGCTGTCCATTGCCCTG

GAACTTGTTAACAACCCGCCTGTTATGTTTTTTGATGAGCCAACGAGT

>MT942646 Procambarus clarkii ABC transporter, subfamily ABCB/MDR mRNA, partial CDS

TCACACATATTTACTATGCTTGAACGCCAACCTGCAATTACAGCATCGCCTAGTGTGGGT

CTACGACTTAACACTCCCGTAACCAGCATAGAGCTAAATGGTGTGCATTTTTCTTACCCG

ACAAGATCTGATGTGCCCATATTATCAGGACTGAATGTTAAAGTGGATAGAGGACAAACT

CTAGCCCTGGTTGGAAGCTCAGGGTGTGGCAAGTCCACCATCATTGGCCTACTGGAGAGA

TTTTATGATGCAAGCAAAGGGAAAGTTTGCATTAGTGGTAAAGATGTACAAGCCCTAAAT

GTTGGCTGGGTGAGAAACCAGCTAGGTCTTGTGTCTCAGGAACCTGTGCTCTTTGATCTC

ACTATTGCCGAAAATATTGCATATGGTGAAAATTGCCGGGAAGTGGGGCATGACGAAATT

GTTAATGCTGCCAAACAAGCCAACATTCACTCATTTGTAGAGTCTCTACCAAATGGCTAT

AATACAAGAGTCGGTGCAAAGGGCACACAATTGTCTGGTGGTCAAAAGCAACGTATAGCA

ATAGCACGAGCACTAATAAGAAACCCAAGTGTGTTGCTACTTGACGAAGCTACCTCTGCT

CTGGACACCGAAAGCGAAAAGGTTGTCCAAGAGGCCTTGGAACAAGCCCAGAAGGGCCGT

ACTAGTATTGTCATCGCTCACCGTCTGTCAACTGTCCAAAATGCCGACACTATTGCAGTA

GTTCAAGGCGGCCGGGTGGTAGAATTTGGTACTCACAAGCAGCTCATCGAGAAGAAGGGA

CACTATTTCTCTCTCTACCAGACTAACAAATAA

______________________________________________________________________________________________________

SET 9. Heme synthesis

>MN110039 Procambarus clarkii delta-aminolevulinic acid dehydratase-like mRNA, partial CDS

ATGACATCTTATGAACCCCCTGCAAAACGCATCCTTCACAGTGGTTACTTTAGCCCTACC

ATGAGACAATGGCAGACATCAAACACGGAAATCCATCCTTGGAATTTGATGTATCCTATA

TTTATTGTGGATGAAGCAGATGCAGAGCAACCCGTGGAAAGTCTACCAGGGGTGACCCGA

TATGGTGTCAACAAACTTGAAGCTGCCCTTAAGCCACTAGTGAAGAATGGTCTTTCCTCT

ATTTTGTTATTTGGCGTTCCCTCAAATATGCCAAAAGATGAACGTGGTTCCAGTGCCGAT

TCACCCAATACGCCAGTTATTGTAGCAGTGAAGATCATTCGTAAAGCATTTCCTGATCTT

CTTGTGGCATGTGATGTGTGTTTGTGCGCCTACACTAGTCATGGTCACTGTGGGATTCTG

AAGAAGGATGGAACTATTGATAACATCCCGAGCATCAAACGGTTGGCTGAACAAGCTGCC

CCTTCGGATATGATGGATGGCAGAGTTGGTGCAATAAAGGCTGCACTTCAGGAAGCTGGT

TTATCAAATAGCGTTTCAGTGCTCTCTTATGCCGTCAAGTTTGCATCTTCTTTCTATGGA

CCATTCAGGGACGCGGCAAAATCTGCTCCAACATTTGGTGATCGGCGATGCTATCAACTG

CCTCCAGGATCATCAGGACTTGCAGCAAGAGCAGCAGATCGTGATGTGGAGGAAGGTGCC

GATATGTTGATGGTGAAACCAGGCATGGCATATCTTGACATTGTGCGTCAGACTAAGGAC

AGATACCCACACTATCCTCTCTTCATCTACCAGGTTTCGGGAGAATATGCTATGTTACAT

>MT942644 Procambarus clarkii 5-aminolevulinate synthase, erythroid-specific, mitochondrial-like isoform X5 mRNA, CDS

ATGCCGTGCCCATTCATGTCACGCCTGTCGGCACAGTTTGTACGTCACTACAGCTCGGCA

TTGGTACGCCAGTATGGGGAGATGTGCCCTGTCATCAGCTCTATGACCTCTACCCGAGCA

TTCAACTCCCTCTCCTCCAACAAGGACCCTGAAGGAGAGAAGAAGTGCCCCTTTTTGAAC

GGCAAGAATTTGGTGAAGCAAGCTAGCCGAGAAGTGCAAGAGGATGTCATTGATCTTTCT

GCTAGGGAACAAGAATTGAGTCTATTCCCATACAATGAATTTTTCCAAGAGCAAATTGCA

CAGAAAAAGGCTGACCATTCCTACCGTGTGTTCAAGAAGGTTGCCCGCAGTGCTAGTGAA

TTCCCCCGAGCTAAGGAGTATTCATGGGGGTACCCGACGGTACCCAGAGGCCATGAAAAG

CTTCGTATAGCTCCTACGCCCCAGCATACACCAGCCATGATAGATTGCTTTGTTGCTGAC

CTAATAACTGTGTGGAAAGATCTCGGATTACCATTATGGAATAGCAGCTGCCCTGAGGAA

TGCACCTTTTGCAAGAAGCCCCTTCTCTTTAATGCATTGGAAGCACGAGAGCGATGCAAC

GCAGACTGTGACAAGCCATACTGTCCCTTGTTGGTGGAATGCCTGTAA

>MH156441 Procambarus clarkii uroporphyrinogen-III synthase-like protein (uros) mRNA, partial cds

ATGAGCAACGTGTGGCTCTTTAAATCAGAAGATAAAAATGACACGCAATATACTGACAAACTTTCTCGGT

CTGGGTTCTCTCCTTTTCATATCCCTGCCCTTTGCTTCAAATTCTGCAACCAAGAATCTCTGAAGAGCTC

ACTGCAGAGTCCCCAAGACCATAGTGGCATTATCTTTACAAGTCAGAGAGCAGTAGAAGCAGTTGCTGAG

ATATATATAAAGTTGCCCCTTAGTTGCCATGCTGGGTGGATTGAGAAAAAGATATTTGTTGTCGGAGATG

CTACTGGAAGAGCTGTTCAAAGTTTATTGAAGCTTACATGTATAGGACATGAATCGGGAAATGCACAACA

ACTCGTTCCAATCATAATTAAGGAGACAGTAGCATTTGACAAACCTCTCTTATACCCTTGTGGCAGTTTA

GCGAAGGATGAATTGCCAAGGCTGCTTGTTAATAATGATCGAGATTTTAAAGCTCTTGTGGTGTATGAAA

CATCACAGCACCCACAACTGAAGTACACTATACAGAAACTGATCTCTGGTGGTCAGAGGCCTACACACAT

GGTGTTTTTCAGTCCATCTGGTGTCAATTTTGCTTTGCCTGTTCTTCAGTCTTTGAGTGTTGATATAACA

GGAGTCAAAATGATTGCAATTGGACCAACAACAAATATTGCTCTGGTTCAACACAAAATTCCGGTGTTAG

GAGTGTGTCCGTCTCCAACTGCAGACAGTCTTGTGCATCTTCTAAATTGTCCACCG

>MN110037 Procambarus clarkii uroporphyrinogen decarboxylase-like mRNA, partial CDS

ATGACAGTGTTTCCCAAGGGTGGCCACTATGCACTCAAGGACCTTGCTGCTCTGAAGTAC

GAGGTCATTGGCATTGATTGGACGGTGGATCCTGTCTTAGCGCGGAAAATTGTGGGCCCA

AATAAAACACTTCAAGGAAACTTGGATCCATGTGCTCTTTATGCAGATAAGAAACACATT

GATACAGCCGTGAAAGAAATGGTGGAGAAGTTTGGAAGGGAGCGCTACATTGCTAACCTG

GGCCATGGGATGTATCCTGATATGGATCCAGAACAATTGGCTGCATTTGTTGAAGCTGTT

CATAAATACTCAAAGAAA

__________________________________________________________________________

SET 10. Diurnal Clock

>QIA97593 Procambarus clarkii calcium-activated potassium channel transcript variant 4 mRNA, CDS

ATGTCCACTGTGGGTTATGGTGATGTCTACTGTCATACTGTCTTTGGAAGAACATTTCTC

GTCTTCTTCCTCCTCGTCGGTTTGGCAATATTCGCAAGCTGTATCCCTGAAATTATAGAC

CTGGTTGGGACTAGATCCAAGTACGGCGGAACACTCAAGAACGAGAGGGGAAGAAGACAC

ATCGTGGTGTGTGGCCATATTACCTACGAGTCTGTCAGCCATTTCCTGAAAGACTTTTTA

CACGAAGATCGTGAGGACGTGGATGTAGAAGTTGTGTTTCTTCACAGAAAGCCGCCGGAT

CTGGAGCTAGAAGGATTATTTAAGCGACACTTCACTACTGTAGAGTTCTTCCAAGGATCC

ATTATGAGTCCCATTGACCTACAACGTGTTAAGGTACATGAAGCTGATGCGTGCCTTGTG

CTGGCTAATAAGTACTGTCAAGATCCTGATGCTGAGGATGCTGCCAATATTATGCGTGTT

ATCTCCATCAAGAATTACTCAGATGATATTAGAGTTATTATTCAGCTAATGCAATATCAC

AATAAGGCCTATCTTTTAAACATTCCCTCTTGGGACTGGAAACGTGGTGATGATGTTATC

TGCTTAGCAGAGCTCAAATTAGGCTTCATTGCACAGTCATGTCTAGCTCCAGGATTTTCT

ACAATGATGGCCAACCTTTTTGCCATGAGATCCTATAAAACTTCTCCAGACATGCAGGCA

TGGCAGAATGACTACCTCTGTGGCACTGGCTGCGAAATGTATACGGAGACACTGTCACCA

AGTTTTGTG

>QIA97594 Procambarus clarkii RNA-binding protein lark-like mRNA, partial CDS

ATGCCTGTGCGGGGAAATACTTTTAAGATATTTGTTGGAAACCTAAGTGACCGTGCAACT

GGCTCGGATATCAGAGAACTCTTTGAAGCTCATGGTACTGTAGTTGAAGCTGATGTTGTG

AAAAATTATGGTTTTGTTCACATGGAAAAGGAAGATGAGGGTCAGGCAGCCATAGAAGCA

CTGAATGGTCACTCTATTCATGGAAAGCCAATGGTAGTTGAGGCCTCCACTGGTGCTAGG

AAGGGTGGAAATCAGAAGACAAAAATATTTATTGGCAATCTTCATAAGGATTCCAAACTT

GAGGAACTGAAAAGCCTGTTTGAAGTATATGGCAGTGTAGTAGAGGCTGACATTTTGACC

AACTATGCCTTTATTCACATGGATGATGAGGCTCAGGCACAACGAGCCATTCGGGAACTA

GATGGATATGAGCTTCATGGTCTGCGCCTTAGGGTCCAAGAATCTACTTCTCGGGTCAGG

CAGCAGGCTGGGATGGGAAATCCAGACATGTGTTACCGCTGTGGGTCAGGTGGTCATTGG

TCCAAGGAATGTCCCAGGGATGGACGCATAGGAGGCTTTCGTTATCCTGATCGAGAGCGA

GGAGGCCGTAGCTTCGGTAGCAGATATGATCCTTATCCACCACCACCACCACCCAGTTAC

GCTAGAGAGCGCATGTTGCGGTATAGGGATGATTTTGACAGATATGATCGTTACGATCGC

TATTATGATGAAGGCTTGTATGAACGTCGTGGTGATCATCCTCCACCACCACCACCCATG

CTAGATGATTTATACGAACGACGGTTGCCACCGCTACCTCCACATCCTGATTATCTGCGA

TATGGTAGACGCTCTCCACCACCTCGTTACCCTCCTCCCCCTCCACCAATGCGTGGTTAC

GGCCCACCGGACCGCCGGCCATAC

_______________________________________________________________________________________________________

SET 11. Crystallin

>MH156430.1 Procambarus clarkii glutathione S-transferase theta (gst-theta) mRNA, partial cds

ATGACTGCTACTTTGACACTGCATGTGGACTACATATCACAGCCTGCCAGAGCTCTCTTGCTCTTGTGCA

GGGCAATCAACGCGCCACACCAGGAAAAATACATGGAGCTATTGCAAGGAGATCATCTGAAGAAGCCGTT

CACCGACCTGAACCCCTTCAGGAAGGTCCCTGTGGTGCAGGACGGAGACGTCCTTATCCTGGAGAGCTGT

ACGGGGTTGCGGTACATCGCGAGCAAGTACGACTCTTCAGGAAAGTGGTACCCGAAGGAGCTGAAGGCGA

GGTGTAAGGTGGACGAATACCTGGACTGGCAGCACCTCAACACCAGGGCCCATGGCGTGGGGTACTTCTA

TAACAAGATTATAGTGCCCATCCTGAAGAAGAGTGAGCCTGACATGAATGTGGTCAACGAACACGAGCTC

AAATTAGGACAAGTTGAAACACAATTTGCAAGTTACTTTCTGGGCTCTAAACCATTCATTACTGGGCAGA

ACATAACTATTGCTGATCTGCTGGCCGCCTGTGAATTCGAGCAGCCCTCGGCAGGTGGCTACCAGCTGTC

CCAACCCATCCTGGAGTACCTCAGCAGAGTCAAGGAGGCGGTAGGCCCGGACTACGATGAACTCCATACT

GCTTCGAGGCAGCTTGCTCAGAAACGGCTCGCT

>MN110030 Procambarus clarkii aldehyde dehydrogenase mRNA, CDS

ATGTTACGGGCCTTCCTCCGTCACTCCTCCCTGCTGAGAGCAGCAGCCACACCCAACCTG

CCCTCAGCGGCAGCGTACTCAGCTCCTGCCATCCCTCAGCCACTGACAACCCCTGATATT

CCCTACACTGGGATCTTCATCAATAATGAGTTCCATAAATCCTTGAATGGGAAGCAGTTC

CCAACTGTCAATCCAACTACAGGAGAGGTCATCACTATGGTGGAGGAGGGTACCAAAGAT

GATGTCGACAAAGCAGTGAAAGCAGCTCGTCAAGCCTTTGAACTCAACTCAGAATGGCGG

CAGATGGATGCAAGCGATCGTGGTCGCCTCCTATACCGTTTGGCTGATCTTATTGAACGT

GACAAAGTTTACCTAGCAAGTCTTGAGACCCTTGATAATGGAAAGCCCTATACGAATTCT

TTTGCGGTCGATGTTGAACTCACCGTTAAAAATCTTCGCTACTTTGCGGGTTGGGCCGAC

AAAATTCATGGTCAGACCATTTCTACTGATGGTCCACACTTTGCATATACAAGGCATGAA

CCAATTGGAGTGTGTGGTCAGATCATCCCCTGGAACTTCCCTCTTCTTATGCAGGCTTGG

AAGTTTGGACCTGCTCTTGCTACAGGCAACACCATTGTTATGAAACTGGCTGAACAGACC

CCACTGACTGGCTTGTATGTGGCTAAACTGGTAGCAGAGGCTGGCTTCCCAGCAGGTGTA

GTTAATGTCATCCCTGGCTATGGTCCAAGCGCTGGAGCGGCCATTGCTTCACATATGGAT

GTTGACAAGGTTGCTTTCACGGGGTCAACAGAGATTGGACATCTGATCCAGCAGGCAGCT

GGAGCTAGCAACCTAAAGCGTGTGACACTGGAACTTGGAGGGAAGAGTCCAAACATTGTT

TTCAAGGATGCTGATTTGGACTATGCTGTTGAACAGGCTCATTTTGGACTATTTTTCAAT

CAGGGTCAGTGCTGCTGTGCTGGATCAAGGATCTTTGTCGAGGATGGCATTTATGATGAG

TTTGTGGAGCGCAGTGTTGAACGTGCCAAGACTCGCAGTGTGGGAGACCCATTTGATTTC

AAGACTGAACAAGGACCACAGGTGGATGGAGAGCAGATGAAGAAGATCTTATCCCTTATT

GAATCTGGCAAGAAGGAAGGAGCCAAAATGTGTACTGGGGGTAAACGTGTCGGCGAGAAA

GGCTTCTTCATTGAACCTACTGTTTTTGCCGATGTGAAAGATAACATGCGTATTGCTAAG

GAGGAAATTTTTGGGCCAGTCCAACAGATCTTTAAGTTTAGTGATATTAATGATGTGATA

AAACGTGCCAACTCCTCGGAGTATGGTCTAGCAGCAGCAGTCTTTACGAAGGATTTGGAC

AAGGCTAACGTGTTCGTGCAAGGCCTTCGTGCTGGCACTGTCTGGATTAACTGCTATGAT

GTACTGAATGCTCAAACTCCTTTTGGAGGCTACAAAATGTCTGGACAAGGACGAGAGAAC

TCCGAGTATGCCTTGCGTAACTACTATGAAGTCAAGGCTGTCATAACCAAATTACCTGTG

AAAAATGCT

>MT601686 Procambarus clarkii alpha-crystallin A chain-like mRNA, partial CDS

CCAATTCGTCGATATCGTCTCTATGATGATCCATTCGATCGCTTCTTTGGTGATCAATTG

GATCTCTTCGATCCATGGAATGATTTCGATGTCTTTCCAACAGCATTAACCATGCGACCC

AATGCTTTTCGATGGGTCAATCAACCCCAACGATTGACTCATTCGTCTTGCAGTGGACAA

AATGGCCATGCTCTGCAATCATCATCACCAGCACCCCATGCTGAAAAATTCCGTGTTCAA

CTCAATGTGGCTGGTTTCAATCCTGAAACCGTCAAAACACATGTCGAGGGTCGCAAAGTC

ATCGTCGAAGCCAAACAGGAAGATCGACAAGGTGAAGGTGATTACAGCATTCGTGAAATT

CGTAAAACATACGATTTACCTGAACATGCTGATGCATCACAATTGGCTTCGTATGTT

>MG910470 Procambarus clarkii small heat shock protein (ibpB) mRNA, partial cds

ATGGAAGGTTTCAAGCACATTCCCGTGAAACTCGGAGACTTCAGCGTCATTGATCAGGAATTCAACTCTA

TTCGCGAAAGATTCGATTCTGAAATGAAGAAAATGGAAGATGAGATGGCTCGCTTCCGAAATGAACTGAT

GAATCGAGAATCGTCTCTCTTCCAGCGCTCCATGCTCACATCTTCCAGCCAACAAGATCAAGCCAGTTCC

AACCAGGGTACTGGGTCTTGGCTGGAAGGGATGAATTCACCTCTTATCCAGCAAGATGGTGACTGTAAGC

AGCTAAAGCTACGGTTTGATGTAAGCCAGTACAAGCCAGAAGAGATCGTTGTTAAGACTGTGGATAATAA

ACTCTTGGTCCATGCCAAACATGAAGAGAAGACAGATAGTCGCTCTGTTTACCGTGAATATAATAGAGAG

TTCCTGCTTCCCAAGGGTACAAACCCAGAACTGATCAAATCTTCACTCTCTAAAGATGGAGTGTTGACTG

TGGAATCGCCACTTCCAGCAATCGTTGGAGGTGATGAAAAAGTCATTCCCATTGCACAGAAC

>MW981273 Procambarus clarkii hypoxia inducible factor 1 alpha gene

ATGTGCTTGAGGAATAACTTGGAGGGTCTTGAGCCCGCTCCTCGGCCCTTCTTAGGACCT

GGCACCTCCAAGACCCAGAAGAACAGTGAGAAGCGGAAGGAGAAGTCTCGGGATGCAGCA

AGATGTCGACGCGGGAAGGAGAGCGAGATCTTCACGGAGCTGGCCAGCGCCCTCCCACTC

CCGCCCCAGACGGTCGCCCAGCTGGACAAGGCCTCCGTCATGAGGCTCACCCTCGCCTTC

CTCAAGACCCGCGCCCTCTGTCAGGCAGGGTTCAGCAAGACGGGTGAGGGCGGAGGCAGC

AAGCTGGATATCGAGATGGACGGACTGCTGCTCAAGGCGCTGGACGGGTTCCTCCTCGTC

CTCTCCACCGACGGCGACATTGTCTTCACCTCCGAGAACATTGTGGCCTTCCTCGGCCTC

CCTCAGGTGGACGTGATGGGCCAGTCACTGTACGAGTACACACATCCCTGCGACCACGAG

GAGGTGCGGGAGCTCATGTCTGTCAAGGAGCACCACGAACCTCGTCACGCCTTCCTTAGG

CTCAAGTGTACCCTTACTGCCAAGGGTCGCTCCGTCAACCTCAAGAGTGCCTCCTACAAG

GTGGTGCAGGTGAGCGGGGAGCTAGTGGGAGGAGAGGAGCAGGCATGGCTGGTGGCTCTG

GGCACTCCTGTACCTCACCCATCCAACATAGAGTTCCCGCTGGACAAGCAAACCTTCGTC

AGCAAACACTCCCTCGACATGAAGTTTACCTACGTCGATGATAACGTGGGAGAGTACTGC

GGGTACACAACGGAGGAGCTGATGGGCCGCTCCCTCTACGAGATGCACCACGCCCTCGAC

TCTGACCTGGTCAAGGACGCGTACAAAACATTGAGAAGCAAAGGCCAGGTGGAGACCGGC

CGCTACAGGTTCCTGGCCCGGGCCGGCGGCTACGTCTGGCTTGTCACTCAGGCCACACTC

ATCCACGGACCCAAGGACAATAAGCCCCAGTACGTCGTGTGTCTCAACTACGTCGTCAGT

GGTGTAGAGTCTGCTGGTGAAATTCTGTCAGAGCTGCAACTGTTGTGTAATAGCAGCAGT

AACATTGACACTAAACACGACGATGGCAGCAGCACCACTAATAACAACAACAACAGCAGC

AGCAGTGTTTCCAACAAGCCAGCTGCACCTGCAGCAGTTGCTGCTCCTCTAGTGTTGAAC

ACCACACCAGCCGTACCACTTCCCAAGCTGGACACACAACCGAAGGTTGAGGAACGAAAG

AGTAGCACAGGTGTAAGAGTGAACCCAGCACCACCTCCAGTTGCAGCCACCTTCAAGATC

TTTACTCCTCGTACAGATGACATGACCAAAGGATACCTTATGTTCTCTGATAATGACCCA

CATTACACAGTGCTGAAAGAAGAGCCCGAGGACCTCACCCACTTGGCACCCTCAGTTGGT

GATACATGTGTGCCTCTTCTAGAGGTTCCTTCCCTAATTCCCGACCACGACCACACCTGT

ATGCTACAGGAGGTCTCTACACTAATCCCTGAACTGGACGATATGTTCACCCTTGACTAT

CACATGCCCATTACTAGTTCTGATGTATTAATCACAACAAGTCCTGATAGCAGTGAAGAT

CGAGAAGAAGCTCAAAAATACCTGTATGATGAAAGTAAACTAATCAGTGGTATCAAGTGC

ATGAACAGTTTAAGTGGAGGAAAGATACTGATAGACAAGAGTGGTTGCTGTACGCCATCG

TCGGACTGTGGAGTGAGCTCCCCAGAACCCCCAAAACCTCTTCTAAGTCAAGCTGCATTA

TCCCCAATACGGGAAAGGAAACAAAATACGGTTTTGTGTGGGGGCAGCCATCCCAGAACA

TCAACAGAGAGCCTCTTCACACACTTGGATGAGATCCGCACACCGGGCTCATCAGAGTCA

TTTGGCAAACTGGATCTCAAACTTGAAGAACGGAACATGGACTCAGATGAGTTTGAGATG

AGAGCTCCATACATTCCTCTCAGTAATGAGATGTTGATGCTGAGCCCTGATGACTTTCTG

TGGGGAGCAGAGCCTGAGCCACTTATATCGCCGAAACACTCTGCATCATCCAGCCGAGAT

GCAAAATGTTGTCATAGTATTATTGACAAGGATGATTCCAACTTGGCTCAGCTTCTGCGT

GACACAGATCCTCACATAACTGGTAGTGGCCCAGGAAGGAACTTGCACATCGAGAATTCC

ACAGGTACTCGAAGTCAGTACCAGCAGAGTAAATTTCTCGATGGTGGAGGGAATTTTGTA

GATCCAAACAAAGTTCTACCAGGACACTCGGGAAGTAAAGATGATTTGGAAGGATCACCA

GGTAGTACATTACTAGTTGATCCGCCACCTGTGATGGTGCAAGAGACTGTTGACCCCCCA

CCTCCATTACTTACTGTTGATACTCACCTTGTAACTTTACCCGTCAAACGGGGACACTCC

CCAAATTCATCTCCCCTCCTCAATCATAAGAAACTGTGTTCACCCTTGTGCCAGCGACAG

CATCCGACCTCGCAACACCATCCTCAAGATGGAGCTGTGCCTCGCCAACTACAACCTGGA

GGCGTGCGCCTCCTAGAAACGCCCAATGCTCCAACTATGCAACAATTATTGATTAGCAAG

GAGCCAATCACAGTTCGAGGAGGACGTCCAGGCGGAGGCATTTCAGCCTCCCAGAATTTC

ATCACAAATAAGAGTCATTCAGTACTTCGCAATCTTCTGAATGTGAATGGCGATGGCAGT

ATAGTCATTGGAGAACCCCAAGCAGGCAGCAGTGGCCTTGCTTCTGCAACATTACGCATC

CCTAGGGACAAGATGACTATGTTGTTAGCTGGTAATGGGGGAATAAATGCTGATGGCCAG

TTGATGTGCTCCAAACTGAGATTAGTGACTGGAAACCACAGTGCCCTCATGCAAGCTGGC

CATTTTGCATTCAAACTGGCTACTAACCCAAATGGGCAGTCTGGCCAAGGCTTGGTGAGG

CGAGGACGGCGACAGGACCCACTTTTGCTGATGGATCCCGAGACTACTATTCCCAACCTG

CTGGATCTGACACAGCAAGACTATGAGGTTAATGCTCCAGCCAGTAATTGTACTTTGCTG

CAGGGGGCAGACTTACTCATGGCACTTGACCAGAGCCTTTAA

_____________________________________________________________________________________________________
